# Incorporation of Chiral Frustrated Lewis Pair into Metal–Organic Framework with Tailored Microenvironment for Heterogeneous Enantio- and Chemoselective Hydrogenation

**DOI:** 10.1021/acscentsci.3c00637

**Published:** 2023-07-27

**Authors:** Yin Zhang, Yao Jiang, Ayman Nafady, Zhiyong Tang, Abdullah M. Al-Enizi, Kui Tan, Shengqian Ma

**Affiliations:** † Department of Chemistry, University of North Texas, Denton, Texas 76201, United States; ‡ School of Chemistry and Chemical Engineering, Hefei University of Technology, Hefei 230009, People’s Republic of China; § Department of Chemistry, College of Science, King Saud University, Riyadh 11451, Saudi Arabia; ∥ National Center for Nanoscience and Nanotechnology, No. 11 ZhongGuanCun BeiYiTiao, 100190 Beijing, People’s Republic of China

## Abstract

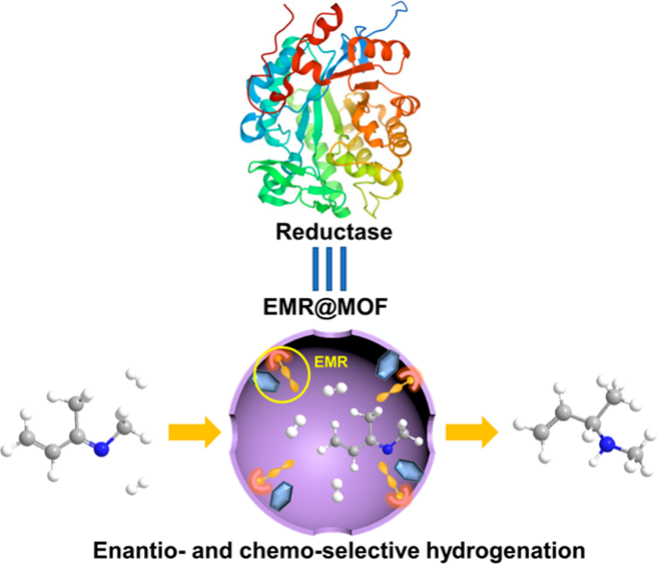

The development of efficient heterogeneous catalysts with multiselectivity (e.g., enantio- and chemoselectivity) has long been sought after but with limited progress being made so far. To achieve enantio- and chemoselectivity in a heterogeneous system, as inspired by enzymes, we illustrate herein an approach of creating an enzyme-mimic region (EMR) within the nanospace of a metal–organic framework (MOF) as exemplified in the context of incorporating a chiral frustrated Lewis pair (CFLP) into a MOF with a tailored pore environment. Due to the high density of the EMR featuring the active site of CFLP and auxiliary sites of the hydroxyl group/open metal site within the vicinity of CFLP, the resultant EMR@MOF demonstrated excellent catalysis performance in heterogeneous hydrogenation of α,β-unsaturated imines to afford chiral β-unsaturated amines with high yields and high enantio- and chemoselectivity. The role of the hydroxyl group/open metal site in regulating chemoselectivity was proved by the observation of a catalyst–substrate interaction experimentally, which was also rationalized by computational results. This work not only contributes a MOF as a new platform for multiselective catalysis but also opens a promising avenue to develop heterogeneous catalysts with multiselectivity for challenging yet important transformations.

## Abstract 2

The incorporation of a chiral frustrated Lewis pair into a metal−organic framework with a tailored microenvironment creates enzyme-mimic regions that realize enantio- and chemoselective hydrogenation.

## Introduction

Selective catalysis has long been a topic in the chemistry research field.^1,2^ Continuous efforts have been devoted to mimic enzymes, the most sophisticated catalyst created by Nature that features high specificity, high selectivity, and high efficiency.^3−10^ The intrinsic strong chirality of enzymes bestows them with high enantioselectivity in chemical transformations, and the auxiliary groups in the vicinity of its active center can regulate other selectivity (e.g., chemoselectivity) through second-sphere interactions.^11−14^ Great progress has been made in mimicking enzymes for selective catalysis in homogeneous systems.^3−6^ However, achieving multiselectivity simultaneously (e.g., enantio- and chemoselectivity) remains very challenging for heterogeneous catalysis.^7−10^

**Scheme 1:**
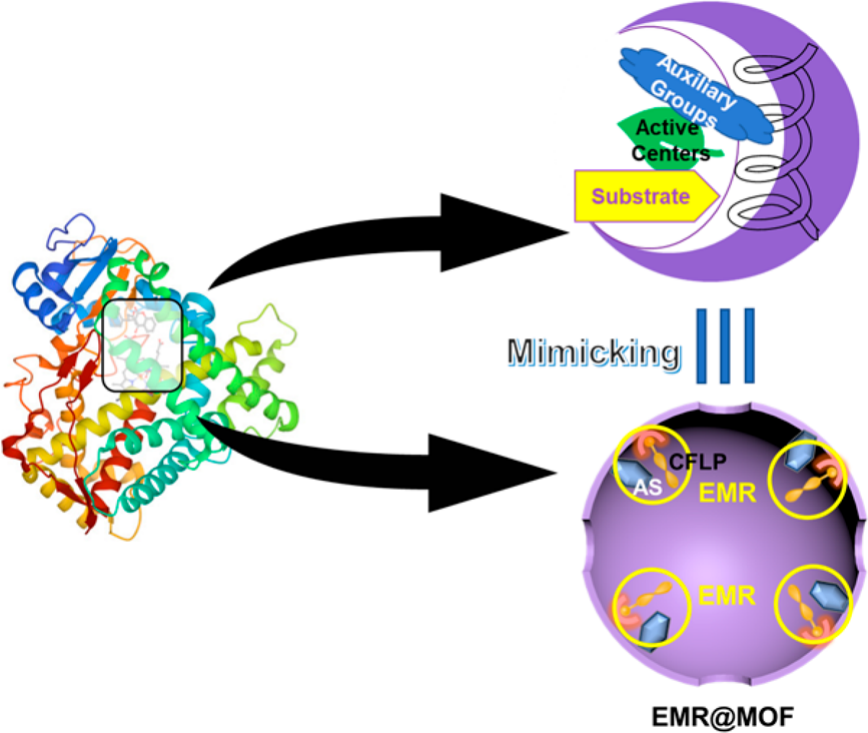
Schematic Illustration of Creating an Enzyme-Mimic Region in the Nanospace of MOF

Metal–organic frameworks (MOFs) have recently been extensively explored as a new class of heterogeneous catalyst;^15−24^ their tunable structures and tailorable pore environments^25,26^ also provide new opportunities for selective catalysis.^27−30^ Although rapid development has been witnessed in the construction of chiral MOFs for enantioselective catalysis,^31−40^ the combination of enantioselectivity with other selectivity (chemoselectivity) is still elusive, particularly for some important reactions in industry like hydrogenation. To realize heterogeneous enantio- and chemoselective hydrogenation, as inspired by enzymes, we propose to create an enzyme-mimic region (EMR) within the nanospace of a MOF (EMR@MOF) by grafting a chiral frustrated Lewis pair (CFLP) onto the MOF pore wall and tailoring the vicinity of CFLP with some auxiliary sites (ASs) (Scheme 1); the chirality nature of CFLP can impart enantioselectivity, and the ASs may regulate chemoselectivity via preferable binding of certain bonds in the substrate reminiscent of enzymes. With a high density of EMR within the confined pore space of a MOF, high yields can be anticipated for hydrogenation reactions in addition to high enantio- and chemoselectivity. Indeed, such an EMR@MOF demonstrated excellent performance in the heterogeneous enantio- and chemoselective hydrogenation of α,β-unsaturated imines to afford chiral β-unsaturated amines under mild conditions.

## Results and Discussion

**Figure 1:**
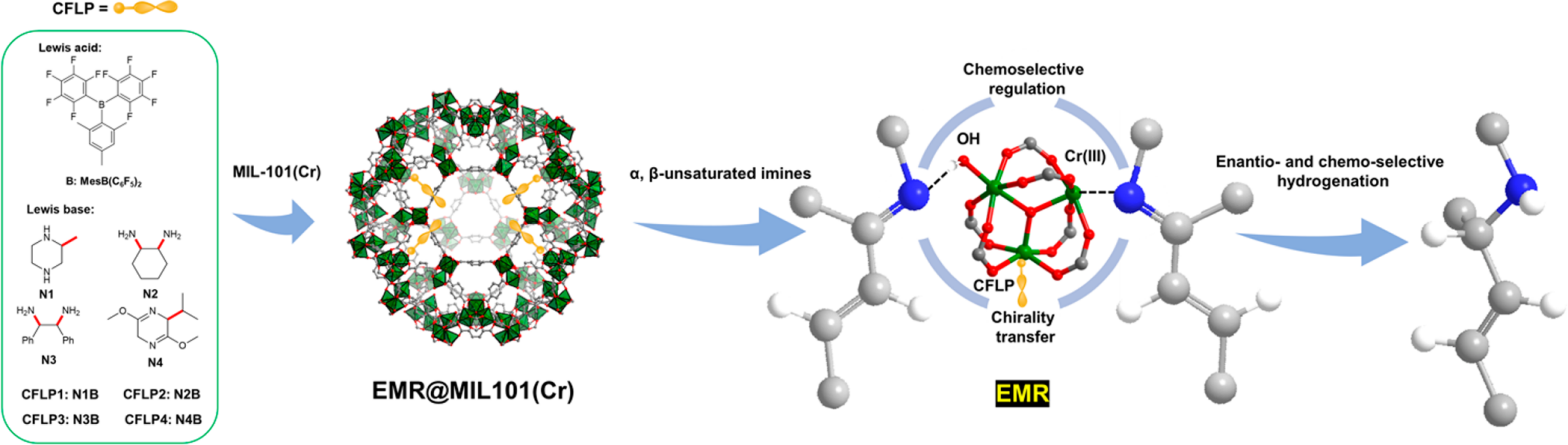
Schematic illustration of preparing EMR@MIL-101 for enantio- and chemoselective hydrogenation of α,β-unsaturated imine. Color code in the structures: gray, C; blue, N; red, O; white, H; green, Cr.

Chiral β-unsaturated amines are the key intermediates for synthesizing chiral β- and γ-amino compounds that account for a substantial proportion of agrochemicals and active pharmaceutical ingredients (Scheme S1).^41−46^ Currently, chiral β-unsaturated amines are obtained in homogeneous systems by asymmetric amination of functional alkenes,^47^ allenes,^48^ alkynes,^49,50^ and internal dienes,^51,52^ in which regio- and enantioselective C–N bond establishment is driven by chiral noble-/transition-metal (Ru, Pd, Au, etc.) complex catalysts or via building a new C–C bond adjacent to the amino group with the assistance of highly active butyllithium reagents for dehydrogenation.^53,54^ Some issues associated with those homogeneous catalysts such as the high cost of noble-metal complexes, difficulty in catalyst/product separation, and inability to recycle catalysts spur the development of a new and more efficient approach for the synthesis of chiral β-unsaturated amines, which we envision can be achieved via enantio- and chemoselective hydrogenation of α,β-unsaturated imines by an EMR@MOF based on chiral frustrated Lewis pair@MOF in a heterogeneous manner. Frustrated Lewis pairs (FLPs) have been widely investigated as a new class of hydrogenation catalyst,^55−61^ and asymmetric hydrogenation has also been successfully implemented with chiral FLP (CFLP) catalysts,^62−64^ but enantio- and chemoselective hydrogenation of α,β-unsaturated imines has not been reported for FLP-based systems. To create EMR in an MOF for heterogeneous enantio- and chemoselective hydrogenation of α,β-unsaturated imines, we anchor the CFLP to the open Cr(III) sites in the secondary building unit Cr_3_(μ_3_-O)(COO)_6_(OH) of the dehydrated MOF MIL-101 (Cr);^65^ during hydrogenation, the CFLP can transfer chirality to the substrate while the hydroxyl on the second Cr(III) site or the remaining third open Cr(III) site can preferably interact with the imine bond (i.e., lower activation energy) over a carbon–carbon double bond in the substrate, thus realizing both enantio- and chemoselectivity similar to those of an enzyme (Figure 1).

Before incorporating CFLPs into MIL-101 (Cr), we first assess the ability of four newly designed CFLPs for H_2_ activation via computational studies to estimate the thermodynamic and kinetic Gibbs free energy of this process at room temperature using the equation Δ*G* = Δ*G*([NH]^+^[BH]^−^) – Δ*G*(N···B) – Δ*G*(H–H) (Figure S1). The obtained positive kinetic Δ*G* values indicate the feasibility of H_2_ activation by CFLPs; meanwhile, the resulting thermodynamic Δ*G* values follow an order of CFLP4 < CFLP3 < CFLP2 < CFLP1, suggesting the H_2_ activation capability order of CFLP4 > CFLP3 > CFLP2 > CFLP1. As a note, the single-point energy calculation was conducted with a solvation correction (based on the gas-phase optimized geometries) to closely simulate the solvent effect of a real reaction using the M06 method in conjunction with the SMD solvation model in solvent (toluene). CFLPs were incorporated into MIL-101(Cr) at different amounts (0.5, 0.75, and 1.0 mmol/g), and the afforded catalysts were labeled in general as CFLP*x*-*y*@MIL-101(Cr) (*x* and *y* are the category and loading amount of CFLPs, respectively).

The computational calculations suggested CFLP4 to be the most active for H_2_ activation among the four newly designed CFLPs, and subsequent characterizations and catalysis investigations were focused on CFLP4 and CFLP4-0.75@MIL-101(Cr). The loading amount of CFLP4 in CFLP4-0.75@MIL-101(Cr) was confirmed by elemental analysis (Table S1) and nuclear magnetic resonance (NMR) (Figure S2). Powder X-ray diffraction (PXRD) analysis suggested that the prepared CFLP4-0.75@MIL-101(Cr) catalyst retained the crystallinity of pristine MIL-101(Cr) (Figure S3), confirming the preservation of the framework integrity. Fourier transform infrared spectroscopy (FTIR) spectra of CFLP4-0.75@MIL-101(Cr) showed the existence of both CFLPs and MIL-101(Cr) signals (Figures S4–S8), indicating the successful integration of these two components. Further analysis of the C–H stretching vibration of CFLP4-0.75@MIL-101(Cr) revealed the red shift around 3000 cm^–1^ as compared with CFLPs, suggesting the formation of FLPs.^27^ N_2_ isotherms at 77 K measured for CFLP4-0.75@MIL-101(Cr) and MIL-101(Cr) indicated a decrease in the Brunauer–Emmett–Teller (BET) surface area after the introduction of CFLP4 into MIL-101(Cr) from 3268 m^2^/g for MIL-101(Cr) to 1382 m^2^/g for CFLP4-0.75@MIL-101(Cr), whereas the pore size distributions remained mainly distributed in the range of 1–3 nm, indicative of well-preserved porosity and minimal pore blockage after CFLPs introduction (Figures S9 and S10).

**Figure 2:**
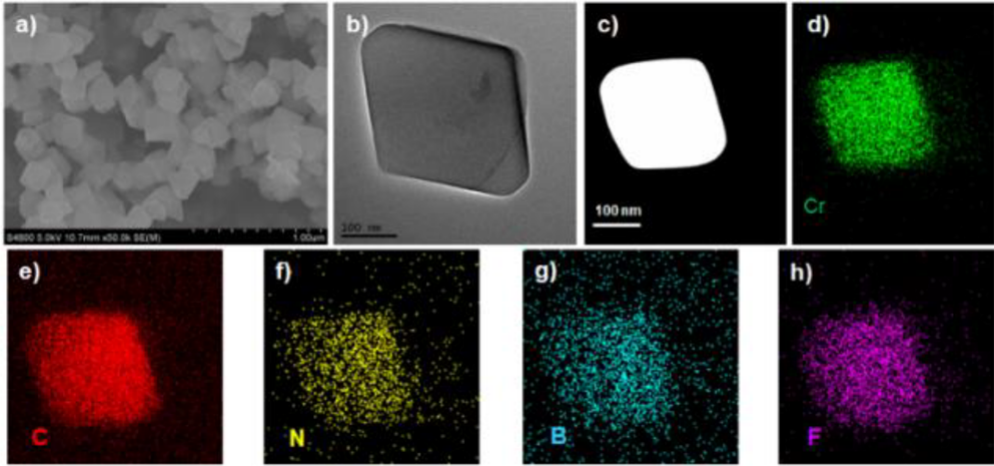
Scanning electronic microscope image (a), transmission electronic microscope image (b), high-angle annular dark-field scanning transmission electron microscopy image (c), and elemental mapping images (d–h) of CFLP4-0.75@ MIL-101(Cr).

CFLP4-0.75@MIL-101(Cr) is thermally stable up to 200 °C, as suggested by thermogravimetric analysis (TGA) (Figures S11–S14). The ^1^H NMR spectra of digested CFLP4-0.75@MIL-101(Cr) attest to the existence of a chiral Lewis base (Figure S15). Scanning electronic microscopy and transmission electronic microscopy images showed the octahedral morphology of CFLP4-0.75@MIL-101(Cr) with a particle size of ca. 200 nm (Figure 2a,b). The high-angle annular dark-field scanning transmission electron microscopy and energy dispersive spectrometer elemental mapping images clearly displayed the existence and distribution of Cr, C, N, B, and F elements in CFLP4-0.75@MIL-101(Cr) (Figure 2c–h), which further verified the presence and dispersion of CFLPs within MOFs. Likewise, the X-ray photoelectron spectroscopy (XPS) data confirmed the signals of Cr, C, N, O, B, and F elements (Figure S16) in CFLP4-0.75@MIL-101(Cr). Closer inspection of the Cr 2p_3_ binding energy in CFLP4-0.75@MIL-101(Cr) revealed an entire 0.5 eV shift compared to MIL-101(Cr) due to the coordination of amines to Cr open sites (Figure S17), which is consistent with the FTIR results.

**Figure 3:**
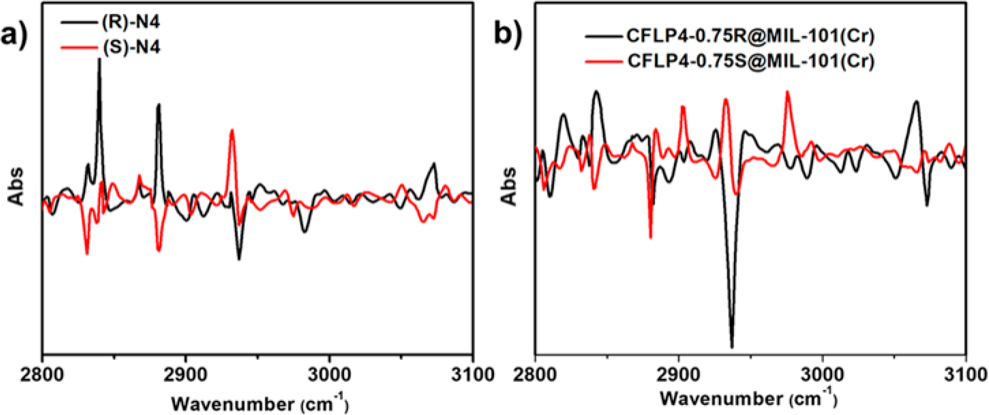
VCD spectra of chiral N4 (2,5-dihydro-3,6-dimethoxy-2-isopropylpyrazine) (a) and CFLP4-0.75@MIL-101(Cr) (b).

For the optical property of chiral (*R* and *S*)-2,5-dihydro-3,6-dimethoxy-2-isopropylpyrazine (N4) and CFLP4-0.75@MIL-101(Cr), they displayed reversal signals in the range of 2800–3000 cm^–1^ (Figure 3), as examined by vibrational circular dichroism (VCD), because of their C–H vibration in the investigated range and different discrimination capacity toward circularly polarized light.

All the prepared catalysts were then examined for catalytic performance in enantio- and chemoselective hydrogenation of α,β-unsaturated imines with isolated yields and enantiomeric excess (ee) values as summarized in Tables 1 and 2 and Tables S2–S4. Unless otherwise mentioned, all of the chiral catalysts were in an *R* configuration. In the context of catalysis, pristine MIL-101(Cr) could not induce the reaction because it is catalytically inert to H_2_ (Table 1, entry 1). On the contrary, CFLP4 itself could realize the hydrogenation of the substrate in a nearly full conversion, validating it as a good catalyst for hydrogenation, but the resultant product was a mixture owing to simultaneous C=C and/or C=N hydrogenation, with a yield of 50% and an ee value of 65% for the target product (Table 1, entry 2). To our delight, CFLP4-0.75@MIL-101(Cr) afforded a substantially higher yield (95%) and ee value (88%) (Table 1, entry 3) compared with those of MIL-101(Cr) and the molecular CFLP4 catalyst. Mechanically mixing MIL-101(Cr) and CFLP4 only led to catalysis performance similar to that of CFLP4 (Table 1, entry 4), underscoring the crucial role of the tailored local environment in MIL-101(Cr) in promoting both enantio- and chemoselectivity. Loading CFLP4 of the *S* configuration into MIL-101(Cr) afforded a comparable yield (94%) but reversal of enantioselectivity (−87% ee), which indicates the general chiral induction of CFLP4-0.75@MIL-101(Cr) (Table 1, entry 5). The observed substantial enhancement of catalysis performance for CFLP4-0.75@MIL-101(Cr) as compared with the molecular CFLP4 catalyst can be ascribed to the high density of EMRs created within MIL-101(Cr), where the high local concentration of CFPL4 molecules facilitates chiral induction, thereby boosting enantioselectivity, and the open Cr(III) sites and the hydroxyl groups preferably interact with the imine bond, thus promoting the C=N chemoselectivity during hydrogenation. In the investigation of how the Lewis acidity of boron compounds influences the catalysis performance, CFLP4-0.75(BCF)@MIL-101(Cr) with tris(pentafluorophenyl)borane (BCF)^64^ instead of MesB(C_6_F_5_)_2_ (Table 1, entry 6), showed a much lower yield of 50% and ee value of 78%, because the BCF with a stronger Lewis acidity than MesB(C_6_F_5_)_2_ can afford higher activity^66^ of the resultant CFLP which leads to the hydrogenation of C=C and C=N simultaneously and thereby a much lower selectivity.

**Table 1:**
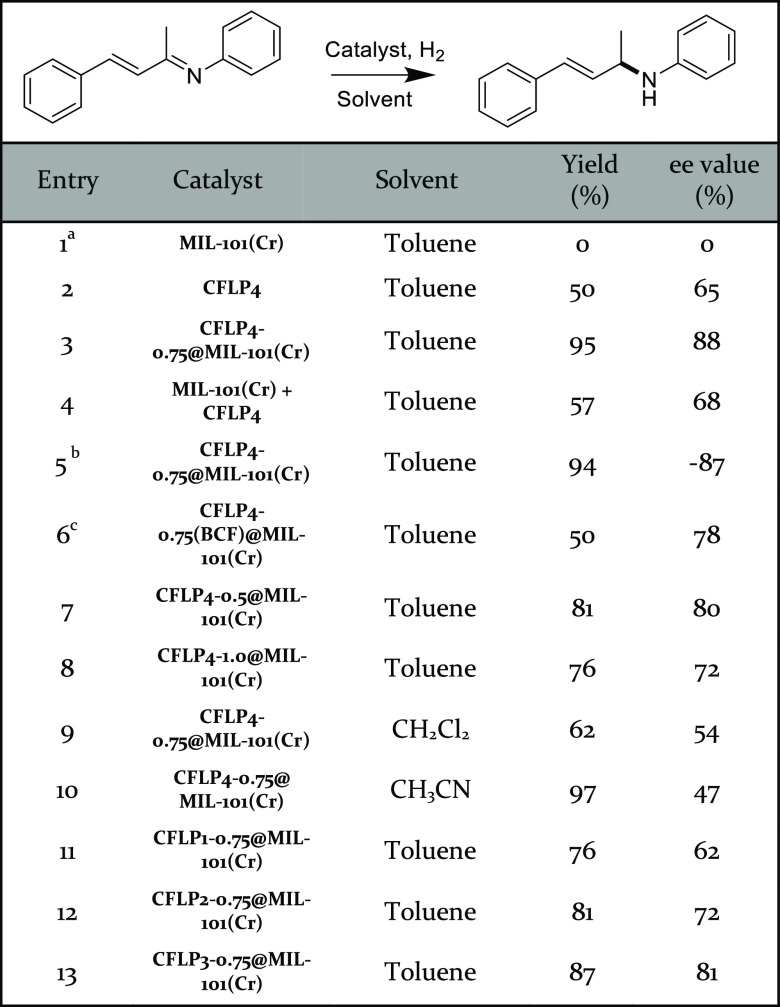
Optimization of Enantio- and Chemoselective Hydrogenation with CFLP*x*-*y*@MIL-101(Cr)

a Unless otherwise stated, the reaction proceeded under the following conditions: 3 mL dry solvent, 20 mg catalyst (7.5 mol %), 0.2 mmol substrate, and 40 bar H_2_, 48 h at room temperature. The yield was determined by the weight of the isolated product, and the ee value was determined by HPLC. The corresponding NMR spectroscopy and HPLC results of products are provided in the Supporting Information.

b CFLP4-0.75@MIL-101(Cr) of *S* configuration.

c MesB(C_6_F_5_)_2_ was replaced by BCF.

**Table 2:**
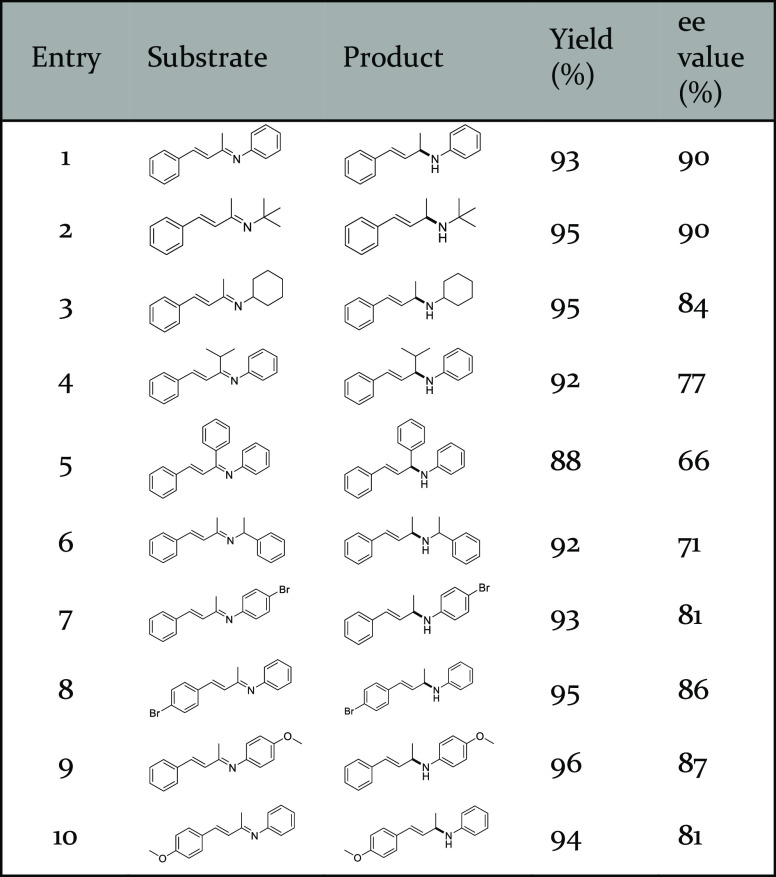
Catalytic Performance of CFLP4-0.75@MIL-101(Cr) toward Enantio- and Chemoselective Hydrogenation with Different Substrates

The effect of EMR density on the catalysis performance was investigated by adjusting the loading amount of CFLP4 into MIL-101(Cr). Reducing the CFPL4 loading amount from 0.75 to 0.5 mmol/g led to a notable decrease in catalysis performance with a smaller yield of 81% and lower ee value of 80% (Table 1, entry 7), which can be attributed to fewer CFLP4 hydrogenation active sites within the MOF. Increasing the CFLP4 loading amount to 1.0 mmol/g resulted in an even more significant decline in the yield (76%) and ee value (72%) (Table 1, entry 8), which can be presumably due to the fact that the excess loading sacrifices the needed free space for mass transfer and adjustment of the optimal molecular configuration. The influence of the solvent on the catalysis performance of CFLP4-0.75@MIL-101(Cr) was also examined. Among the three commonly used solvents of CH_2_Cl_2_, toluene, and CH_3_CN, toluene afforded the best performance. When using CH_2_Cl_2_ as the solvent, substantially poorer performance was obtained with only a 62% yield and 54% ee value (Table 1, entry 9), which can be ascribed to the poor solubility of substrates in CH_2_Cl_2_. A slightly higher yield (97%) was achieved when CH_3_CN was used as the solvent (Table 1, entry 10), but a much lower ee value (47%) was observed stemming from the formation of achiral FLPs between CH_3_CN and BCF.^67^ The heterogeneous nature of CFLP4-0.75@MIL-101(Cr) was verified by a leaching test (Figure S18) and the fact that it exhibited no notable decrease in catalysis performance (Figure S19) in a 5-cycle reuse test with well maintained crystallinity (Figure S20), porosity (Figure S21), and composition (Figures S22 and S23), highlighting its superior reusability and regenerability compared with its homogeneous counterpart. The catalysis performances of EMRs based on the other three newly designed CFLPs with the same optimal loading amount of 0.75 mmol/g in MIL-101(Cr) were also assessed (Table 1, entries 11–13). Compared with CFLP4-0.75@MIL-101(Cr), CFLP1-0.75@MIL-101(Cr), CFLP2-0.75@MIL-101(Cr), and CFLP3-0.75@MIL-101(Cr) exhibited moderate to good catalytic performance with respective yields of 76%, 81%, and 87% and ee values of 62%, 72%, and 81%, which were in line with the computational calculation results.

The above results clearly illustrated that enantio- and chemoselective hydrogenation can be achieved simultaneously in the heterogeneous system via creating EMR within an MOF, and the catalysis performance of EMR@MOF can be readily tuned by simply changing the density and type of EMR.

Given the superior catalysis performance of CFLP4-0.75@MIL-101(Cr), the scope of substrates in chemo- and enantioselective hydrogenation was expanded. Substrates with different N-substitution from the phenyl group to *tert*-butyl or cyclohexyl could be converted to target products in comparable yields and ee values (Table 2, entries 1–3). On changing the α-substitution of α,β-unsaturated imine from methyl to isopropyl or phenyl, a slight decrease in catalysis performance (Table 2, entries 4 and 5) was observed, which could be attributed to the increasing steric hindrance at C=N that makes it less efficient to approach active sites. Similarly, the introduction of a methyl group on both sides of α-N positions led to reduced catalysis performance (Table 2, entry 6). In addition, bromo-substituted substrates exhibited compatible yields (93%, 95%) and ee values (81%, 86%) (Table 2, entries 7 and 8). Furthermore, substrates with an electron-donating group (−OCH_3_) could also be transformed to the target products in good yields and ee values (Table 2, entries 9 and 10). All these results showcased CFLP4-0.75@MIL-101(Cr) as an excellent heterogeneous catalyst for enantio- and chemoselective hydrogenation.

CFLP@MOF catalysts thus had the capacity of enzymes to achieve enantio- and chemoselectivity, although the reaction rate in our system is lower than that in enzyme systems considering the elongated reaction time. Additionally, CFLP@MOF catalysts exhibit selectivity to substrates of specific functional groups and size (Table S3). This characteristic is similar to the substrate selectivity observed in enzymes. Specifically, α,β-unsaturated imines with replacement of C=N by C=O or C=C could not be converted into the corresponding chiral product due to the inactivity of CFLP for C=O reduction and unsuccessful establishment of a second-sphere interaction between C=C and auxiliary sites of a hydroxyl group/open metal site. The substrate with a larger size (∼1.4 nm) than the window size of porous MIL-101(Cr) (∼1.0 nm) showed no conversion, as it could not pass through the MOF window, suggesting that the reaction proceeds inside pores of CFLP@MOF catalysts with size selectivity on substrates.

To gain some understanding of the mechanism of enantio- and chemoselective hydrogenation catalyzed by CFLP4-0.75@MIL-101(Cr), complementary supports from both experiments and computations have been obtained. The theme of our work is to mimic an enzyme’s capability to achieve multiselectivity (i.e., enantio- and chemoselectivity herein) by incorporating a chiral frustrated Lewis pair into a MOF to mimic the intrinsic chiral environment in an enzyme system to impart enantioselectivity by tailoring the vicinity of a chiral frustrated Lewis pair with some auxiliary sites of a hydroxyl group/open metal site to mimic the second-sphere interactions in an enzyme system to regulate chemoselectivity and thus to afford enantioselectivity and chemoselectivity simultaneously during hydrogenation reactions. To validate the hypothesis of the second-sphere interaction, it is necessary to ascertain interactions between the substrate and the auxiliary sites. Previously, porous heterogeneous catalysts built on Cr trimers have been extensively investigated for important transformations. Of particular interest to chemists is the discovery of unconventional chemoselectivity in relation to Cr trimers, which was supported by reliable theoretical calculations.^27,28^ However, the origin of selectivity, particularly the interaction between Cr trimers and the substrate, remains unclear, owing to the lack of direct experimental proof.

**Figure 4:**
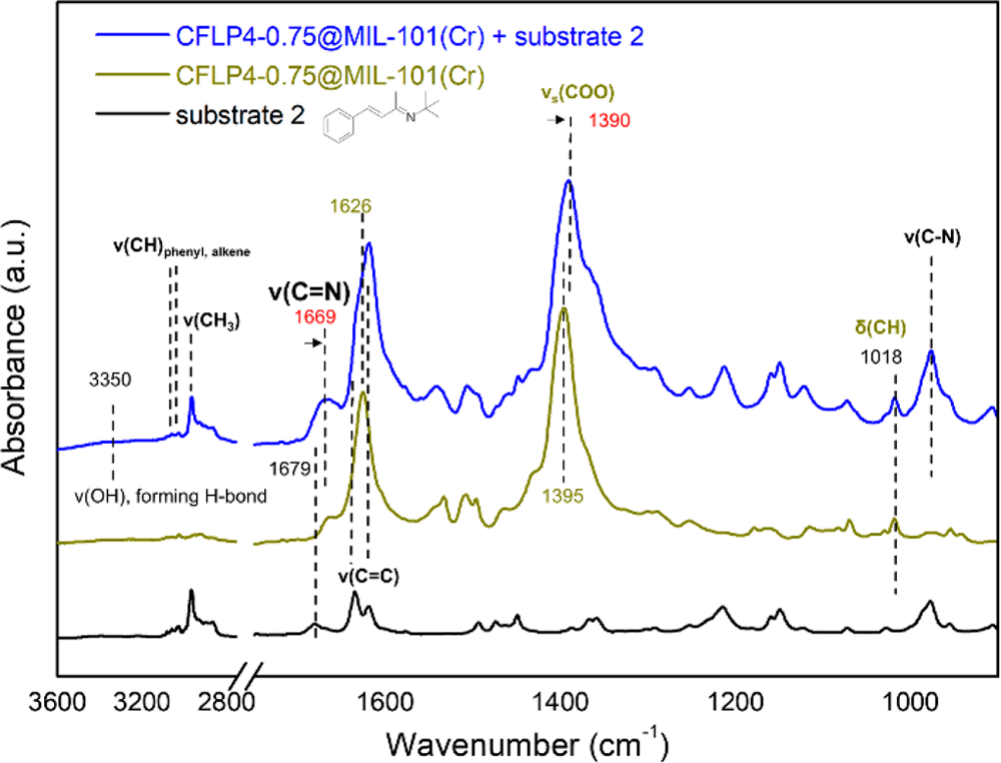
IR spectra of CFLP4-0.75@MIL-101(Cr) + imine (top), CFLP4-0.75@MIL-101(Cr) (middle), and liquid imine (bottom). Notations and acronyms: ν, stretch; δ, in-plane deformation; ph, phenyl; s, symmetric.

In our system, to probe the interaction between the imine substrate and MOF structure, we measured IR spectra of the CFLP4-0.75@MIL-101(Cr) sample before and after immersion into substrate 2 (liquid) and subsequent evacuation under vacuum to remove residual unbound substrate 2. The adsorbed substrate 2 species in the MOF structure is typified by their characteristic bands associated with imine,^68^ vinyl,^69^ phenyl,^70^ and methyl vibrations,^70^ as shown in Figure 4. Careful examination of these modes reveals that most of them remain in the same position as in the liquid form except for ν(C=N),^68,70^ which shows a red shift of 10 cm^–1^ (1679 to 1669 cm^–1^), suggesting a weakened C=N bond due to its interaction with the MOF structure. It is also worth noting that the ν(C–N) mode manifests as an intense feature around 976 cm^–1^, evidencing the linkage of nitrogen to open metal sites on the Cr trimers and thus forming an imine-metal complex, as we have formerly established in studying a number of amine molecules binding within MIL-101(Cr) and MOF-74(Ni) compounds.^71−73^ Interestingly, pronounced changes were observed in the carboxylate stretching mode of Cr trimers after adsorbing imine; i.e., the ν_s_(COO) band shows a red shift of 5 cm^–1^ compared to the value (1395 cm^–1^) in CFLP4-0.75@MIL-101(Cr). In contrast, the nearby phenyl ring mode δ(CH) on the benzene-1,4-dicarboxylic acid linker at 1018 cm^–1^, whose frequency displays high sensitivity to its chemical environment change,^74−76^ remains unaffected after adsorbing imine. These observations clearly suggest that imine interacts primarily with Cr trimers. The ν(OH) mode on Cr trimers is unfortunately too weak to detect in the spectrum of CFLP4-0.75@MIL-101(Cr). However, in imine-loaded CFLP4-0.75@MIL-101(Cr), we observed the appearance of a broad feature at ∼3350 cm^–1^. It is well-known that the ν(OH) band exhibits an intensity enhancement and line broadening upon forming hydrogen bonds.^77^ Therefore, it is reasonable to suggest that there is also interaction of some substrate 2 species with a hydroxyl group through H-bonding, giving rise to such a broad feature. As such, the preferable interaction of C=N over C=C in α,β-unsaturated imine with Cr trimers has been observed experimentally. To further understand this process from the viewpoint of energy, substrate adsorption energies over a hydroxyl group and an open Cr(III) site of Cr trimers with an imine bond were calculated to be −7.3 and −6.8 kcal/mol (Figure S24), respectively. Besides, contrast tests have also indicated the presence of a hydroxyl group and an open Cr(III) site of Cr trimers for attaining target chemoselectivity (Table S4). Collectively, adequate support from experiments and computations together confirms the selective activation of C=N over C=C in α,β-unsaturated imines by the remaining open Cr(III) site and hydroxyl group of Cr trimers, promoting the selective reduction of imine bonds during subsequent hydrogenation to achieve chemoselectivity.

As for the hydrogenation activity with CFLP4-0.75@MIL-101(Cr), we used XPS to discern the H_2_ activation process. The B 1s binding energy of CFLP4-0.75@MIL-101(Cr)-H_2_ showed a 0.4 eV decrease compared with CFLP4-0.75@MIL-101(Cr) because of the increased electron density of the B element (Figure S25), suggesting the heterolytic H_2_ fission analogous to that in classic FLPs.^55^ Accordingly, the calculated Gibbs energy profile of the reaction process also confirms its reasonable occurrence (Figure S26).

Last but not least, it is crucial to comprehend how the incorporation of a chiral frustrated Lewis pair into a MOF can mimic the intrinsic chiral environment in an enzyme system to impart enantioselectivity. The chirality transfer from the chiral base of CFLP to the product is the origin of enantioselectivity. Although the majority of the reports of enantioselective FLP catalysis have focused on using a chiral Lewis acid,^62−64^ there is promising potential to explore chiral Lewis base based CFLP catalysts due to the abundance and synthetic ease of chiral Lewis bases. However, achieving successful asymmetric induction of a Lewis base can be affected by two key factors. First, there is a competition between the chiral Lewis base and the achiral substrate (imine, ketone, etc.). Second, the order and position of the hydride (delivered by a Lewis acid) and the proton (delivered by a Lewis base) conversion to the substrate play a significant role. In the case of imine hydrogenation, the achiral imine substrate itself can act as a Lewis base in FLP chemistry or the initial unbound protonation of the imine nitrogen by the chiral Lewis base can lead to unsatisfactory asymmetric induction. From previous reports on enantioselective FLP catalysis using a chiral Lewis base,^78^ it is vital to avoid achiral Lewis base competition and carefully consider all the possible noncovalent interactions between the FLP catalyst and the substrate, as well as the reaction mechanism, to prevent detachment of the chiral Lewis base from the prochiral system before having performed the asymmetric induction. Specifically, successful examples have demonstrated that the proton and hydride transfer to the substrate occurs in a concerted fashion under the regulation of noncovalent interactions (hydrogen bonding and π–π). To avoid achiral Lewis base competition, alternative approaches has also been developed based upon relay catalysis by using an achiral borane and a chiral phosphoric acid for asymmetric hydrogenation.^78a−78c^ In our system, we used a stepwise strategy to anchor a chiral diamine and an achiral Lewis acid in turn; this strategy can ensure the interaction of the achiral Lewis acid with the chiral Lewis base rather than the achiral imine substrate. Subsequently, the imine substrate added to the catalyst preferably interacted with Cr(III) open sites/hydroxyl groups inside pores as demonstrated by FT-IR results (Figure 4). In these two ways, we circumvent the competition of achiral imine with a chiral Lewis base. More importantly, unlike most of the unsatisfactory examples failing to convey chiral induction with P/B frustrated Lewis pairs, we think that the N/B system has an advantage of forming hydrogen bonding during the protonation step, which is analogous to the mechanism of ketone reduction by an FLP catalyst^79^ and thus builds the connection between the chiral amine and the subsequent hydride delivery step, thereby facilitating the chirality transfer from the chiral center to the target site (Scheme S2). In particular, the hydrogen-splitting chiral FLP4(R)-H2 intermediate interacts with the substrate (2*E*,3*E*)-*N*,4-diphenylbut-3-en-2-imine through hydrogen bonding and π–π stacking interactions under the chiral induction of individual chiral centers and enforced chiral environment inside nanopores of MIL-101(Cr), supporting the generation of *S* product due to a lower energy of 1.5 kcal/mol over the *R* product, which is consistent with the experimental results. Conclusively, the creation of an enzyme-mimic region within a confined nanospace via the incorporation of a chiral frustrated Lewis pair into the MOF with a tailored microenvironment promotes the reaction efficiency by reducing solvation and dissociation of the catalyst and substrates, imparts substrate activation through second-sphere interactions, and enhances the hydrogen bonding interactions for chirality transfer, thus collectively contributing to the observed performance.

## Conclusion

In summary, enantio- and chemoselectivity in the heterogeneous system has been achieved via the creation of an enzyme-mimic region (EMR) within the nanospace of a MOF (EMR@MOF), as illustrated in the context of grafting a newly designed chiral frustrated Lewis pair (CFLP) onto the pore wall of MIL-101(Cr) with auxiliary sites of a hydroxyl group and an open Cr(III) site within the vicinity of CFLP for heterogeneous enantio- and chemoselective hydrogenation of α,β-unsaturated imines. Guided by computational studies on a series of newly designed CFLPs, CFLP4-0.75@MIL-101(Cr) that features a high density of EMRs demonstrated excellent catalysis performance in transforming α,β-unsaturated imines to chiral β-unsaturated amines under mild conditions with high enantio- and chemoselectivity in addition to good recyclability/regenerability. This work lays a solid foundation to develop a MOF as an efficient multiselective heterogeneous catalysis system. In addition, the creation of a reaction region with the tailored environment of the subtle difference in binding/interaction with the substrate to afford specific selectivity reminiscent of enzymes as showcased herein can be extended to other heterogeneous systems for multiselective catalysis.

## Methods

### Materials

Unless otherwise stated, all of the chemicals were purchased from international chemical suppliers and used without further purification.

### Characterization

Nuclear magnetic resonance (NMR) spectra were recorded on a Varian 500 spectrometer. Powder X-ray diffraction (PXRD) data of solid samples were recorded on a Bruker AXS D8 Advance A25 powder X-ray diffractometer (40 kV, 40 mA) with Cu Kα (λ = 1.5406 Å) radiation. Fourier transform infrared spectroscopy (FTIR) was performed with a PerkinElmer Spectrum Two FT-IR spectrometer. Vibrational circular dichroism (VCD) spectra were obtained using a Bruker Optics PMA 50 FTIR spectrometer. Transmission electron microscopy images and energy dispersive spectrometry (EDS) were attained using an FEI Tecnai F30 microscope. X-ray photoelectron spectra (XPS) were conducted on a PHI 5000 Versaprobe Scanning XPS Microprobe instrument with UPS. High-performance liquid chromatography (HPLC) was recorded with an Agilent 1220 Infinity II LC System. Thermogravimetric analysis (TGA) was measured by a TA Instruments Q50 Thermogravimetric Analyzer. N_2_ sorption isotherm measurements were performed at 77 K on a Micromeritics ASAP 2020 instrument. The elemental data were collected on a Thermo Flash Smart elemental analyzer.

### General Method for the Preparation of CFLP@MIL-101(Cr)

In a glovebox, 100 mg of activated MIL-101(Cr), 4 mL of dry toluene, and chiral diamine were mixed in a 20 mL vial with stirring for 24 h. The solid was separated from the suspension by centrifugation and then washed three times with dry toluene (2 × 3 mL). Following that, the obtained solid sample was dispersed in 4 mL of dry toluene in a 20 mL vial to which MesB(C_6_F_5_)_2_ was added and stirred for 1 day. Finally, the target sample was obtained after separation from the mixture and washing three times with dry toluene (2 × 3 mL).

### Preparation of CFLP4-0.5@MIL-101(Cr)

In a glovebox, 100 mg of activated MIL-101(Cr), 4 mL of dry toluene, and 2,5-dihydro-3,6-dimethoxy-2-isopropylpyrazine (10.0 mg) were mixed in a 20 mL vial with stirring for 24 h. The solid was separated from the suspension by centrifugation and then washed three times with dry toluene (2 × 3 mL). Following that, the obtained solid sample was dispersed in 4 mL of dry toluene in a 20 mL vial to which MesB(C_6_F_5_)_2_ (24.3 mg) was added and stirred for 1 day. Finally, the target sample was obtained after separating from the mixture and washing three times with dry toluene (2 × 3 mL).

### Preparation of CFLP4-0.75@MIL-101(Cr)

In a glovebox, 100 mg of activated MIL-101(Cr), 4 mL of dry toluene, and 2,5-dihydro-3,6-dimethoxy-2-isopropylpyrazine (14 mg) were mixed in a 20 mL vial with stirring for 24 h. The solid was separated from the suspension by centrifugation and then washed three times with dry toluene (2 × 3 mL). Following that, the obtained solid sample was dispersed in 4 mL of dry toluene in a 20 mL vial to which MesB(C_6_F_5_)_2_ (36 mg) was added and stirred for 1 day. Finally, the target sample was obtained after separating from the mixture and washing three times with dry toluene (2 × 3 mL). The calculated density of CFLP4 per MOF unit and Cr_3_O cluster was 0.51.

### Preparation of CFLP4-1.0@MIL-101(Cr)

In a glovebox, 100 mg of activated MIL-101(Cr), 4 mL of dry toluene, and 2,5-dihydro-3,6-dimethoxy-2-isopropylpyrazine (20 mg) were mixed in a 20 mL vial with stirring for 24 h. The solid was separated from the suspension by centrifugation and then washed three times with dry toluene (2 × 3 mL). Following that, the obtained solid sample was dispersed in 4 mL of dry toluene in a 20 mL vial to which MesB(C_6_F_5_)_2_ (48 mg) was added and stirred for 1 day. Finally, the target sample was obtained after separating from the mixture and washing three times with dry toluene (2 × 3 mL).

### General Procedure for the Enantio- and Chemoselective Hydrogenation

Typically, except for the H_2_ exchange process, all the procedures were finished in the glovebox. 20 mg of catalyst and 3 mL of dry toluene were stirred in a 25 mL autoclave at room temperature and 40 bar pressure of H_2_ for 12 h. After that, 0.2 mmol of substrate was placed in the autoclave and the mixture was stirred at room temperature and 40 bar pressure of H_2_ for 48 h. When the reaction was completed, centrifugation was conducted to separate the solid and liquid. The target product was obtained after disposal of the liquid by column chromatography. The corresponding yield was determined by weight percentage, and the enantiomeric excess (ee) value was evaluated by high-performance liquid chromatography (HPLC). The solid was washed three times with dry toluene (2 × 3 mL) for further use.

### Recycling Tests for CFLP4-0.75@MIL-101(Cr)

A 20 mg portion of CFLP4-0.75@MIL-101(Cr) and 3 mL of dry toluene were stirred in a 25 mL autoclave at room temperature and 40 bar pressure of H_2_ for 12 h. Subsequently, 0.2 mmol (substrate 1) was placed in the autoclave, and the mixture was stirred at room temperature and 40 bar pressure of H_2_ for 48 h. When the reaction was completed, the solid and liquid were separated by centrifugation. The target product was obtained after disposal of liquid by column chromatography. The corresponding yield was determined by weight percentage, and the ee value was evaluated by HPLC. CFLP4-0.75@MIL-101(Cr) was washed three times with dry toluene (2 × 3 mL) for reuse. The above procedure was repeated five times.

## Supporting Information Available

Additional figures, tables, and schemes as described in the text and NMR spectra, HPLC results, and coordination in the theoretical calculation to explicitly reflect catalyst characterization, catalytic performance, and mechanistic investigations (PDF)
