# Supplementary material for: Incorporation of Chiral Frustrated Lewis Pair into Metal–Organic Framework with Tailored Microenvironment for Heterogeneous Enantio- and Chemoselective Hydrogenation
Source: ACS Cent Sci. 2023 Jul 27;9(8):1692–701. doi: 10.1021/acscentsci.3c00637 (PMC10451035; doi:10.1021/acscentsci.3c00637)
Supplement: Supplementary file 1 — oc3c00637_si_001.pdf [file oc3c00637_si_001.pdf]

Supporting Information

Incorporation of Chiral Frustrated Lewis Pair into Metal-Organic Framework with Tailored Microenvironment for Heterogeneous Enantio- and Chemo-Selective Hydrogenation

Yin Zhang†, Yao Jiang‡, Ayman Nafady#, Zhiyong Tang*, ₸, Abdullah M. Al-Enizi#, Kui Tan, † and Shengqian Ma*, †

†Department of chemistry, University of North Texas, Denton, TX 76201, USA

‡School of Chemistry and Chemical Engineering, Hefei University of Technology, Hefei 230009, China

#Department of Chemistry, College of Science, King Saud University, Riyadh, 11451, Saudi Arabia

₸National Center for Nanoscience and Nanotechnology, No.11 ZhongGuanCun BeiYiTiao, 100190 Beijing, P.R. China

Email: shengqian.ma@unt.edu (Shengqian Ma); zytang@nanoctr.cn (Zhiyong Tang)

Chemicals and reagents

All the chemicals were purchased from commercial resources and used without further purification, unless otherwise stated. Corresponding information of representative chemicals were given below: magnesium turnings (Alfa, 99.5%), iodine (Alfa, 99.5%), magnesium sulfate (Fisher Chemical, 99%), chromic nitrate nonahydrate (Sigma, 99.5%), terephthalic acid (Sigma, 99.5%), S-piperazine (J&K, 99%), R-piperazine (J&K, 99%), (1S, 2S)-1,2-cyclohexanediamine (J&K, 98%), (1R, 2R)-1,2-cyclohexanediamine (J&K, 98%), (1S, 2S)-1,2-diphenyl-1,2-ethanediamine (J&K, 98%), (1R, 2R)-1,2-diphenyl-1,2-ethanediamine (J&K, 98%), (S)-2,5-dihydro-3,6-dimethoxy-2-isopropylpyrazine (Ark, 98%), (R)-2,5-dihydro-3,6-dimethoxy-2-isopropylpyrazine (Ark, 98%), bromopentafluorobenzene (TCI, 98%), boron (tri) fluoride etherate (TCI, 99%), 2-bromomesitylene (Sigma, 99.5%), benzylideneacetone (Thermo scientific, 98%), (E)-5-methyl-1-phenylhex-1-en-3-one (TCI, 98%), (2E)-1,3-diphenylprop-2-en-1-one (TCI, 98%), 4-(4-bromophenyl)but-3-en-2-one (Ambeed, 95%), 1-(P-methoxyphenyl)-1-buten-3-one (Ambeed, 95%), 4-methoxyaniline (TCI, 98%,), aniline (Alfa, 99%), 4-bromoaniline (TCI, 99%), *tert*-butylamine (TCI, 98%), cyclohexanamine (TCI, 99%), 1-phenylethylamine (Thermo scientific, 98%), anhydrous ether (Sigma, 99.5%), anhydrous toluene (Sigma, 99.5%), anhydrous dichloromethane (Sigma, 99.5%), anhydrous acetonitrile (Sigma, 99.5%), sodium deuteroxide (Thermo Scientific, 30 wt%).

Computational method

All data in this study was calculated with the Gaussian 16 software package[1] and was optimized at the M06 level of density functional theory (DFT).[2] The basis set 6-31G (d) was selected for all atoms. Vibrational frequency analysis was computed to ensure the points that the minimum have no imaginary frequency and the transition states have only one imaginary frequency. In order to consider the solvent effects, the solvation corrected single-point energy calculations (based on the gas-phase optimized geometries) were calculated by using the M06 method in conjunction with the SMD solvation model[3] in solvent (toluene). In the solvation-corrected calculations, 6-311++G (d, p) basis set was chosen for all nonmetal atoms. The single-point energy corrected relative free energies are used for discussion throughout the text.

Synthesis of $MesB(C_6F_5)_2$[4]

The preparation of C_6F_5MgBr ether solution: Magnesium turnings (1 g, 41.2 mmol) and 2 mg iodine were suspended in a 100 mL two-neck flask with 20 mL ether, in which bromopentafluorobenzene (5 mL, 40.1 mmol) was added dropwise to avoid a quick reflux under N_2. And the mixture was heated to start the reaction, following that, the color of solution changed from light brown to white and finally it turned black. After the complete addition of bromopentafluorobenzene, the reaction mixture was stirred at room temperature for 30 minutes.

The preparation of MesMgBr THF solution: 1,2-dibromoethane (1.0 mL) was added dropwise into a mixture of Mg turnings (0.577 g, 23.7 mmol) and anhydrous THF (20 mL) under N_2 and stirring. Then MesBr (3 mL, 19.6 mmol) was slowly added into the mixture in 30 minutes. After the addition was completed, the reaction was refluxed for a further 1 hour. Finally, the mixture was cooled down to room temperature and stirred for 3 hours.

The above C_6F_5MgBr ether solution was added dropwise to an Et_2O solution (20 mL) of $BF_3{\times}OEt_2$ (2.5 mL, 2.84 g, 20.0 mmol) at 0 °C. The reaction mixture was stirred at 0 °C for an additional 3 hours and then the THF solution of MesMgBr was slowly added into. The reaction mixture was stirred at 0 °C for two more hours, after that, warmed up to room temperature and stirred overnight. Subsequently, solvents were removed in vacuo and the residue was pulverized in the glovebox. The resulting brown powder was put into sublimation (130 °C and $1{\times}10^{-3}$ mbar) twice whereupon the pure $MesB(C_6F_5)_2$ was collected as the white needles. ^{1}H-NMR (500 MHz, C_7D_8): δ = 6.70 (s, 2H), 2.13 (s, 3H), 1.99 (s, 6H). ^{19}F-NMR (500 MHz, C_6D_8): δ = -129.8, -145.5, -161.5.

The preparation of MIL-101(Cr)[5]

MIL-101(Cr) was prepared based on the previous procedure. In detail, $Cr(NO_3)_3.9H_2O$ (5 g, 12.5 mmol), terephthalic acid (2.1 g, 12.5 mmol) and 50 mL H_2O were mixed together in a 100 mL PTFE lining which was transferred to the autoclave. Then the autoclave was heated to 220°C and kept for 24 hours. After cooling down to room temperature, the green solid was separated from

the suspension by centrifugation at 10000 r/min and it was washed with DMF three times. After that, the solid was immersed in 200 mL DMF and heated under 100^oC while stirring for 24 hours. Subsequently, the sample was conducted Soxhlet extraction with ethanol for two days. Finally, MIL-101(Cr) was activated by degassing under 150^oC to reach 5×10^{-2} mbar pressure before use.

Synthesis of α, β-unsaturated imines[6]

Ketone (30 mmol), amine (30 mmol), magnesium sulfate (5 g), and 40 mL CH_2Cl_2 were mixed in a 100 mL flask. After stirring under room temperature for 24 hours, the mixture was filtered to obtain the solution. The solvent of the solution was removed under vacuum and the residue was purified by ethanol/ethyl acetate recrystallization.

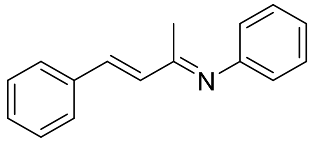

Synthesized from benzylideneacetone and aniline. 1H NMR (DMSO, 400 HZ): δ = 2.25 (s, 3H), 6.85 (d, 1H), 7.15 (m, 3H), 7.28-7.36 (m, 5H), 7.40-7.53 (m, 3H).

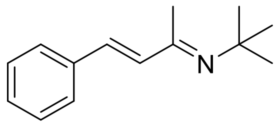

Synthesized from benzylideneacetone and tert-butylamine. 1H NMR (DMSO, 400 HZ): δ = 1.23 (s, 9H), 2.11 (s, 3H), 6.85 (d, 1H), 7.23-7.30 (m, 4H), 7.45 (m, 2H).

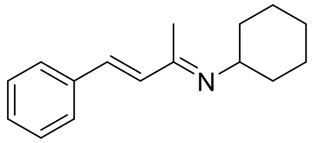

Synthesized from benzylideneacetone and cyclohexanamine. 1H NMR (DMSO, 400 HZ): δ = 1.32-1.47 (m, 10H), 2.18 (s, 3H), 3.36 (m, 1H), 6.78 (d, 1H), 7.35-7.42 (m, 4H), 7.54 (m, 2H).

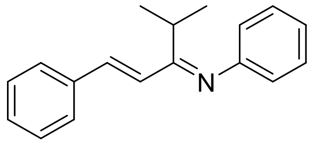

Synthesized from (E)-5-methyl-1-phenylhex-1-en-3-one and aniline. 1H NMR (DMSO, 400 HZ): δ = 1.10 (d, 6H), 2.56 (m, 1H), 6.75 (d, 1H), 7.18 (m, 3H), 7.27-7.32 (m, 5H), 7.62 (m, 3H).

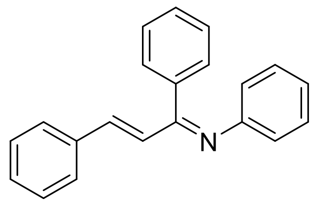

Synthesized from (2E)-1,3-diphenylprop-2-en-1-one and aniline. 1H NMR (DMSO, 400 HZ): δ = 7.19 (m, 3H), 7.31 (m, 1H), 7.38-7.43 (m, 9H), 7.57-7.60 (m, 4H).

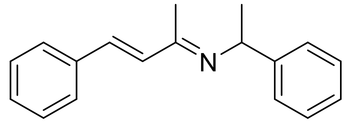

Synthesized from benzylideneacetone and 1-phenylethylamine. 1H NMR (DMSO, 400 HZ): δ = 1.43 (d, 3H), 2.18 (s, 3H), 4.68 (m, 1H), 7.28-7.41 (m, 8H), 7.58 (m, 2H).

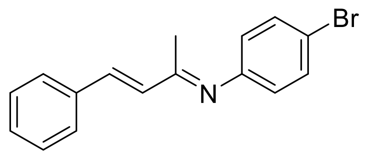

Synthesized from benzylideneacetone and 4-bromoaniline. 1H NMR (DMSO, 400 HZ): δ = 2.23 (s, 3H), 6.88-7.04 (m, 3H), 7.30-7.39 (m, 3H), 7.50 (m, 1H), 7.57 (m, 4H).

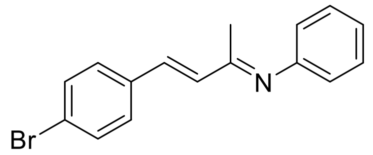

Synthesized from 4-(4-bromophenyl)but-3-en-2-one and aniline. 1H NMR (DMSO, 400 HZ): δ = 2.19 (s, 3H), 6.92 (d, 1H), 7.05-7.17 (m, 3H), 7.35 (m, 2H), 7.52-7.64 (m, 5H).

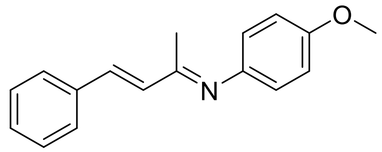

Synthesized from benzylideneacetone and 4-methoxyaniline. 1H NMR (DMSO, 400 HZ): δ = 2.20 (s, 3H), 3.68 (s, 3H), 6.89-7.00 (m, 3H), 7.21 (m, 2H), 7.33-7.38 (m, 3H), 7.44-7.55 (m, 3H).

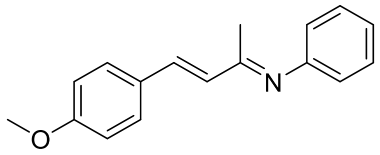

Synthesized from 1-(P-methoxyphenyl)-1-buten-3-one and aniline. 1H NMR (DMSO, 400 HZ): δ = 2.23 (s, 3H), 3.80 (s, 3H), 6.88-6.96 (m, 3H), 7.16 (m, 3H), 7.43 (m, 2H), 7.52-7.63 (m, 3H).

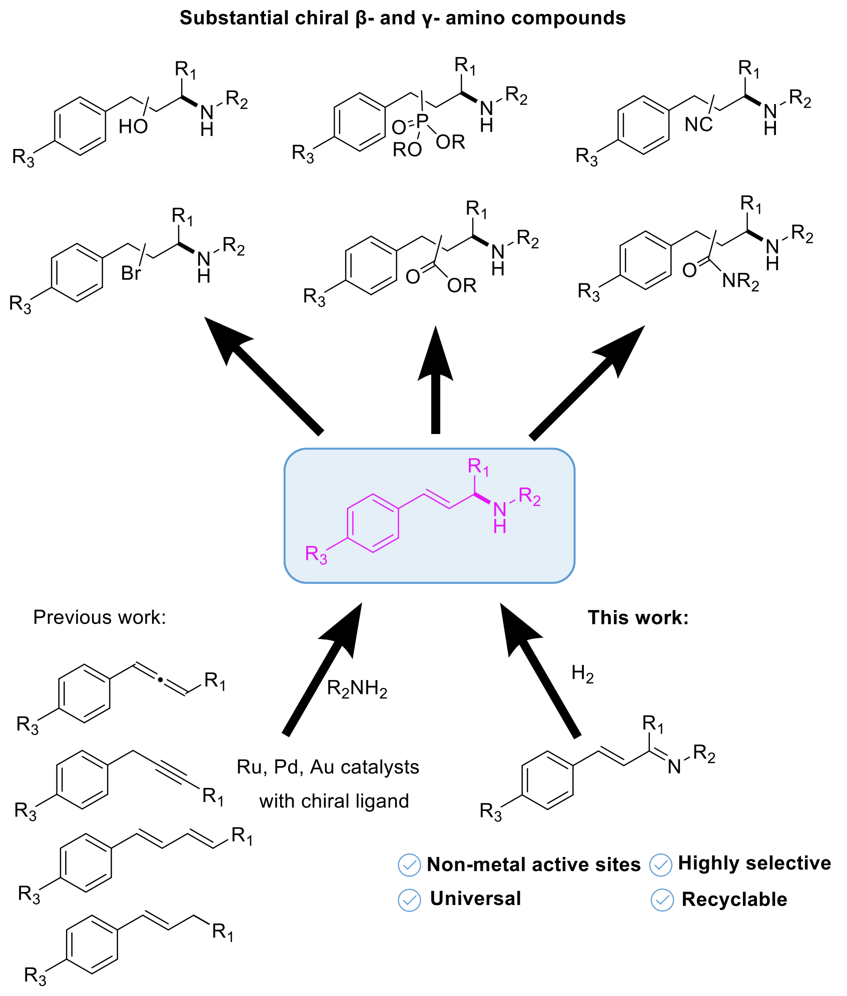

Scheme S1. The rational synthesis of chiral β-unsaturated amines as substantial intermediates to produce chiral β- and γ- amino compounds.

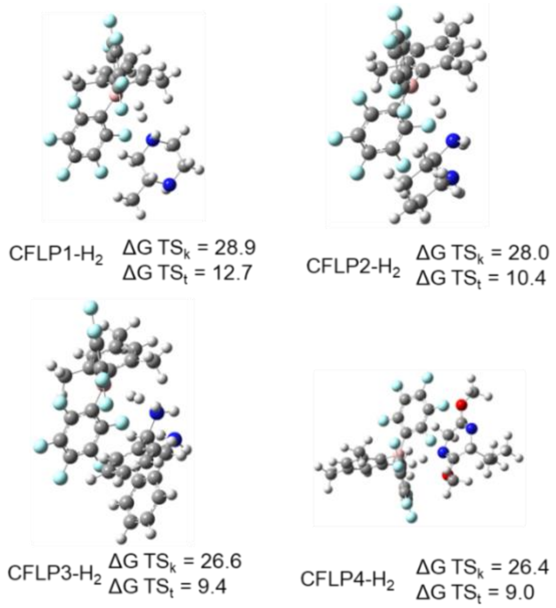

Figure S1. The calculated kinetic (k) and thermodynamic (t) Gibbs energy of H_2 activation with different kinds of CFLPs. The color balls in the structures stand for pink = B, gray = C, blue = N, red = O, light blue = F, and white = H.

Table S1. The elemental analysis of CFLPx-0.75@MIL-101(Cr)

CFLP4-0.75@ MIL-101(Cr)	Element	Measured weight (%)	Calculated weight (%)
	C	46.8	46.6
	H	2.3	2.6
	O	21.9	22.2
	N	1.6	1.4
CFLP3-0.75@ MIL-101(Cr)	Element	Measured weight (%)	Calculated weight (%)
	C	49.1	48.9
	H	2.8	2.6
	O	21.5	21.8
	N	1.8	1.4
CFLP2-0.75@ MIL-101(Cr)	Element	Measured weight (%)	Calculated weight (%)
	C	47.4	46.5
	H	2.7	2.6
	O	22.1	22.9
	N	1.8	1.5
CFLP1-0.75@ MIL-101(Cr)	Element	Measured weight (%)	Calculated weight (%)
	C	46.9	46.2
	H	2.6	2.6
	O	22.4	23.1
	N	1.7	1.5

The MOF catalyst was solvent exchanged with CH_2Cl_2 and then activated under dynamic vacuum at 60°C for 12 hours before the elemental analysis.

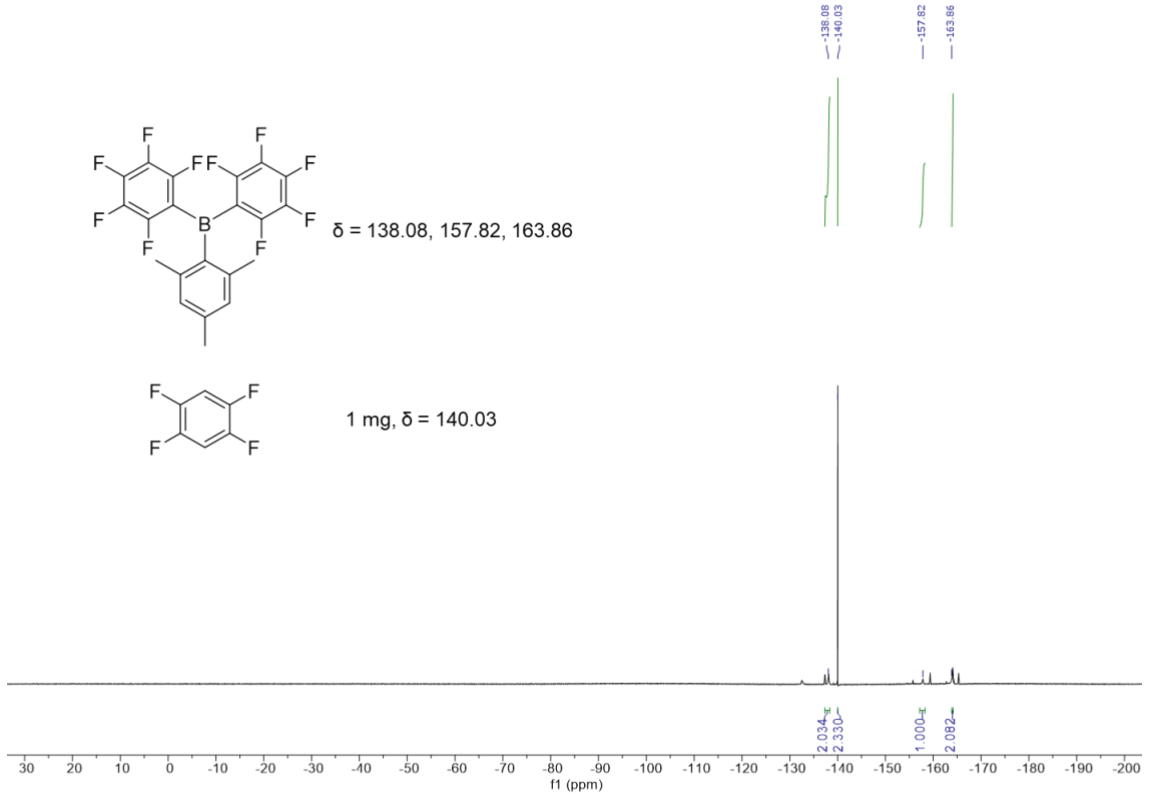

Figure S2. Determination of $MesB(C_6F_5)_2$ in CFLP4-0.75@MIL-101(Cr) with ^{19}F NMR spectrum.

4 mg CFLP4-0.75@MIL-101(Cr) was digested by 0.2 mL NaOD (30 wt%) under sonication and mixed with 0.8 mL DMSO-d6 in a 3 mL tube, where was further added with 2 mg 1,2,4,5-tetrafluorobenzene as an external standard. In subsequence, 0.5 mL solution was used for the ^{19}F NMR test. As a result, the calculated amount of $MesB(C_6F_5)_2$ is 0.71 mmol per gram MIL-101(Cr).

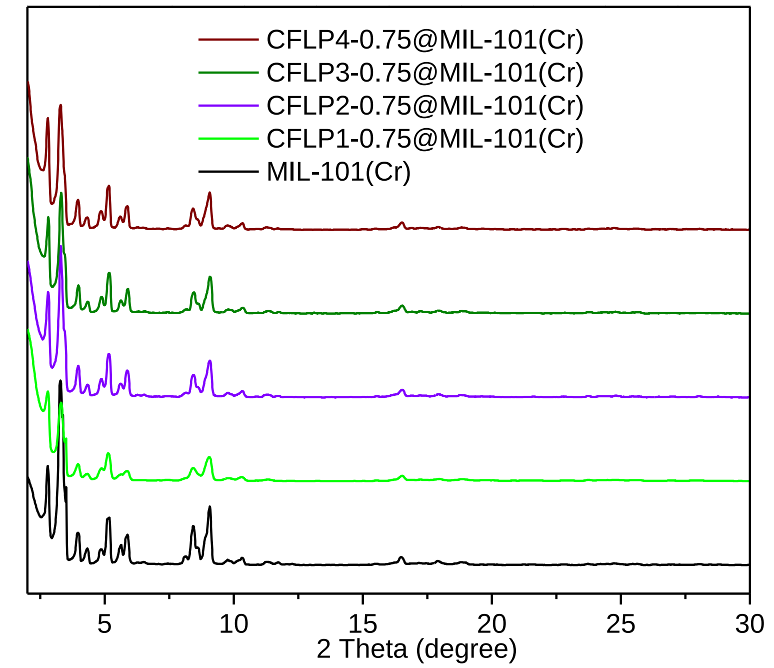

Figure S3. PXRD patterns of porous CFLPx-0.75@MIL-101(Cr) and MIL-101(Cr).

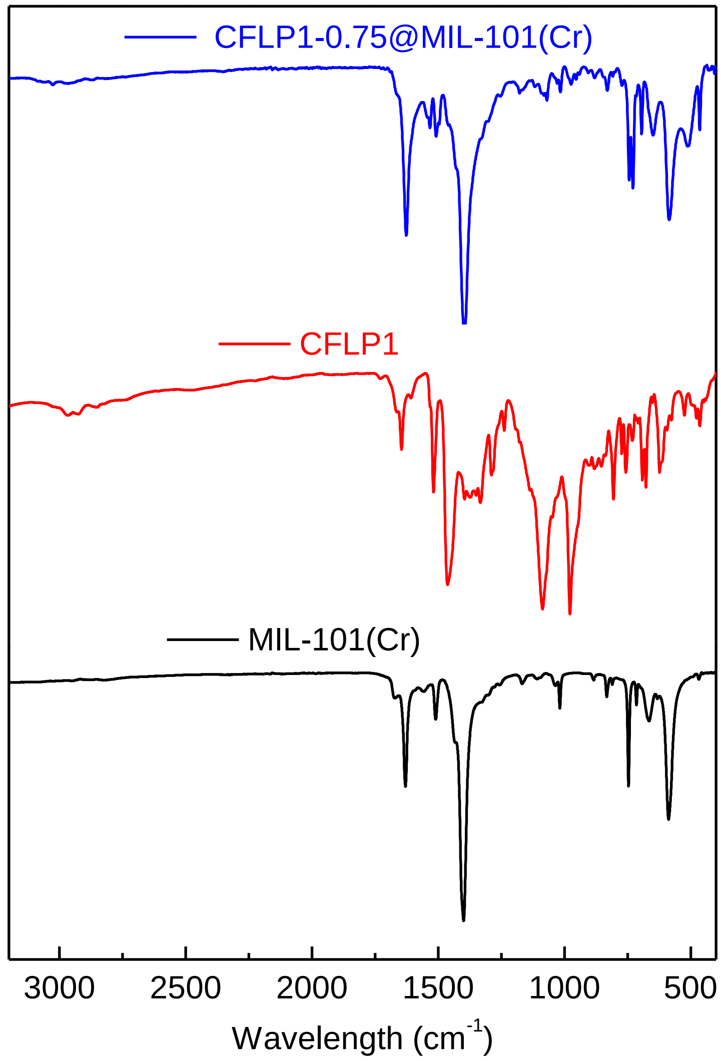

Figure S4. FTIR spectra of CFLP1-0.75@MIL-101(Cr), CFLP1, and MIL-101(Cr).

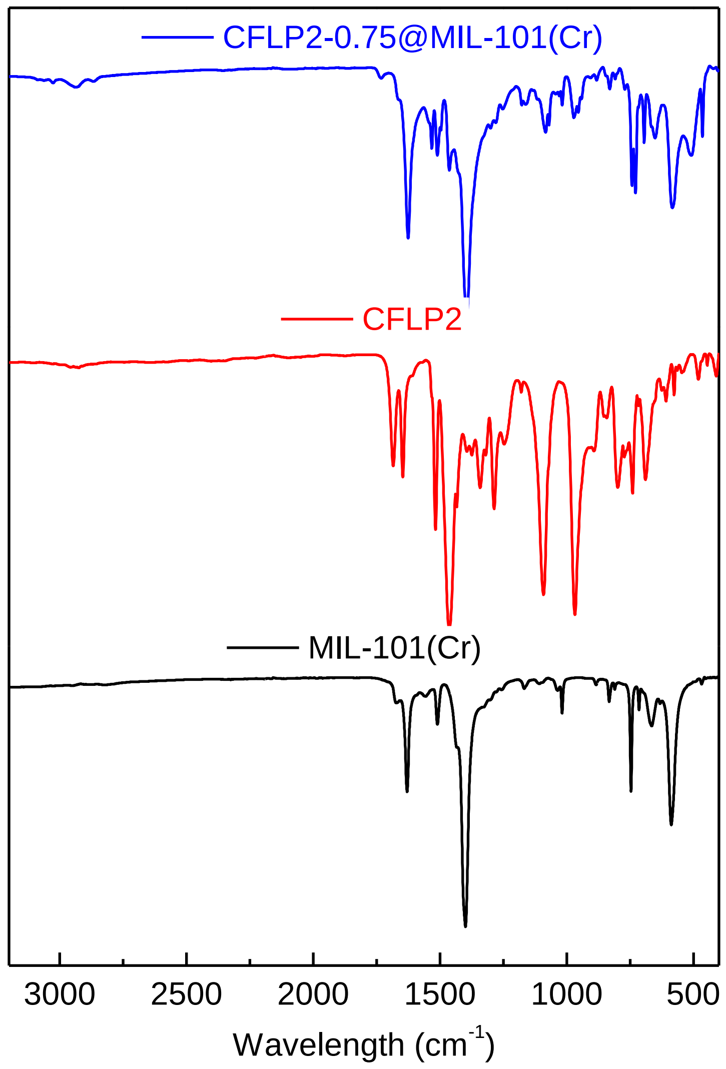

Figure S5. FTIR spectra of CFLP2-0.75@MIL-101(Cr), CFLP2, and MIL-101(Cr).

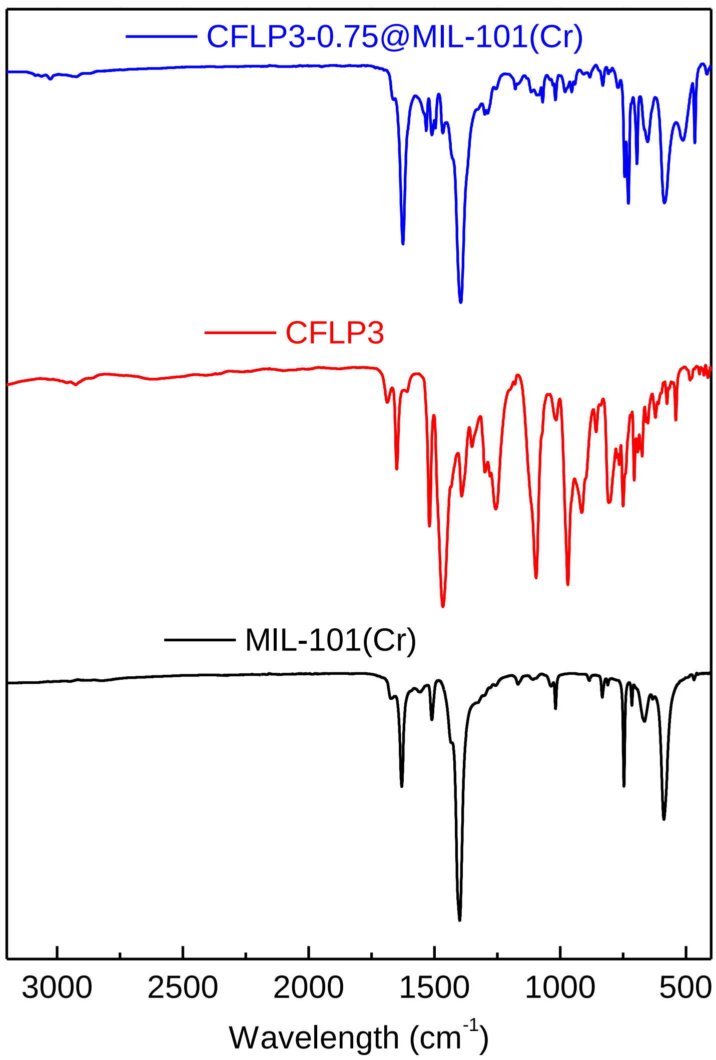

Figure S6. FTIR spectra of CFLP3-0.75@MIL-101(Cr), CFLP3, and MIL-101(Cr).

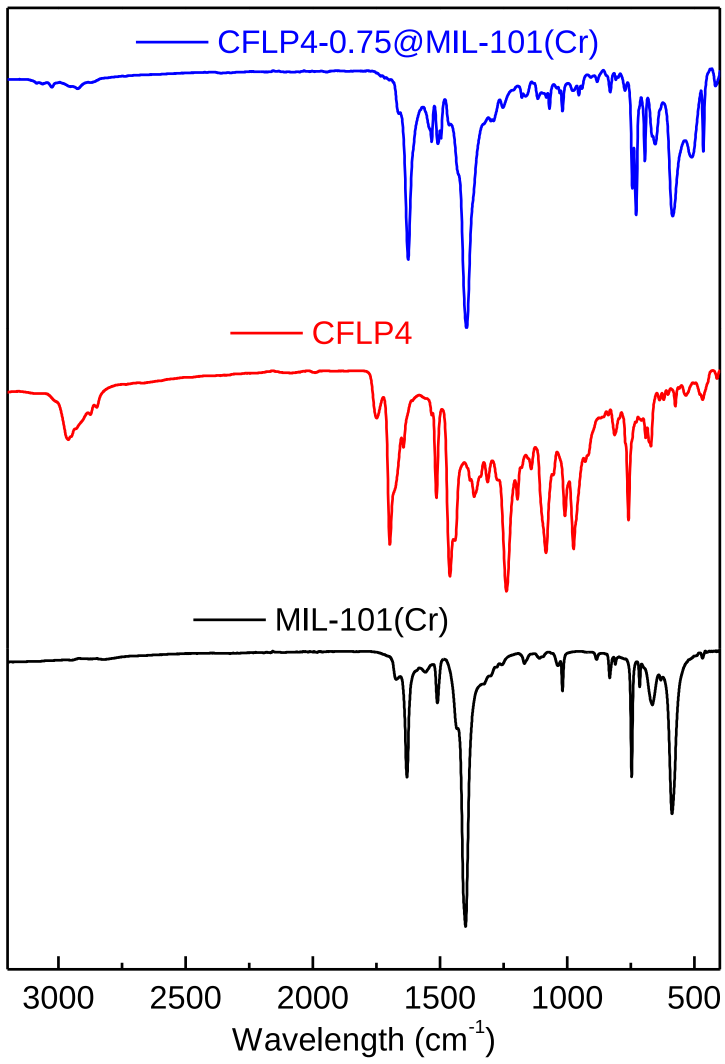

Figure S7. FTIR spectra of CFLP4-0.75@MIL-101(Cr), CFLP4, and MIL-101(Cr).

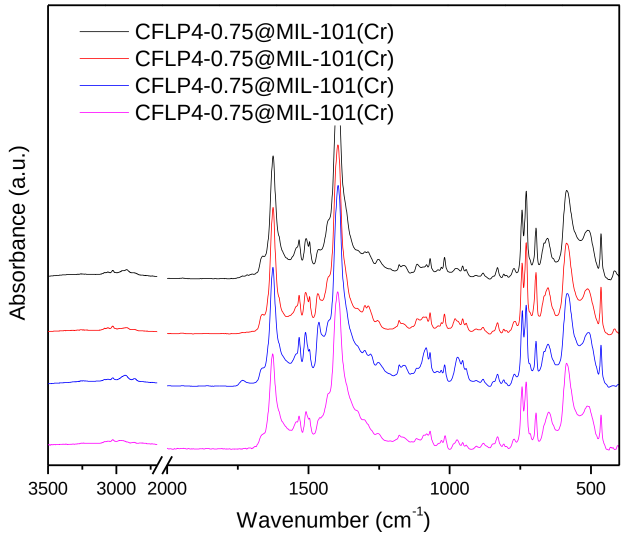

Figure S8. FTIR spectra of CFLP@MIL-101(Cr) in absorbance mode.

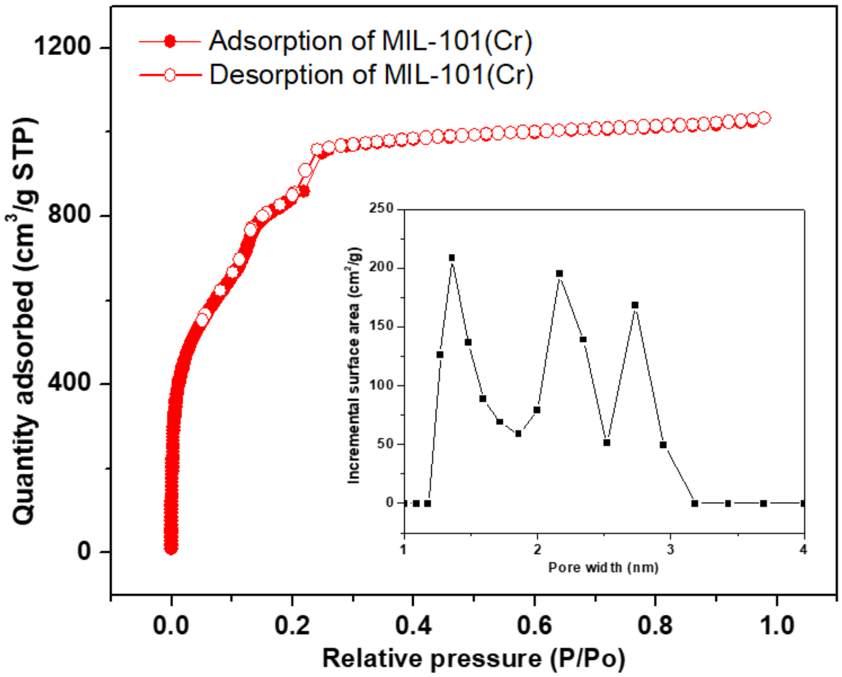

Figure S9. N_2 sorption test and pore size distribution of MIL-101(Cr).

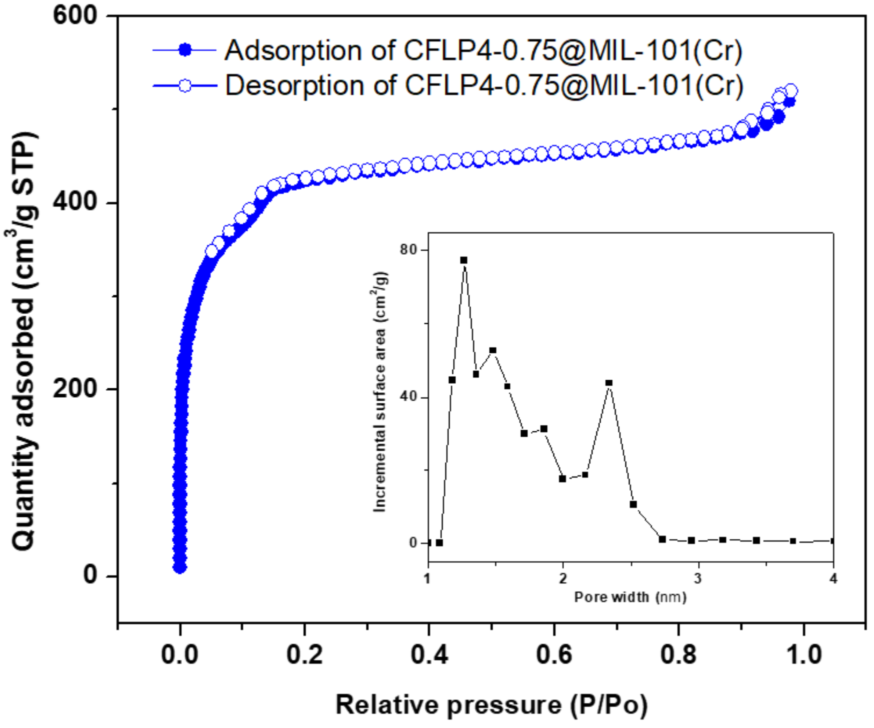

Figure S10. N_2 sorption test and pore size distribution of prepared CFLP4-0.75@MIL-101(Cr).

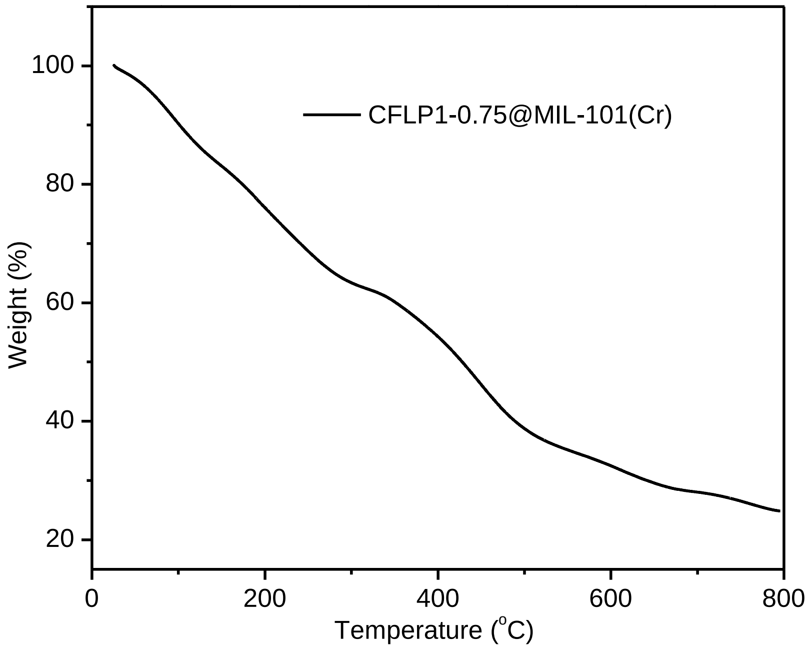

Figure S11. TGA curve of CFLP1-0.75@MIL-101(Cr).

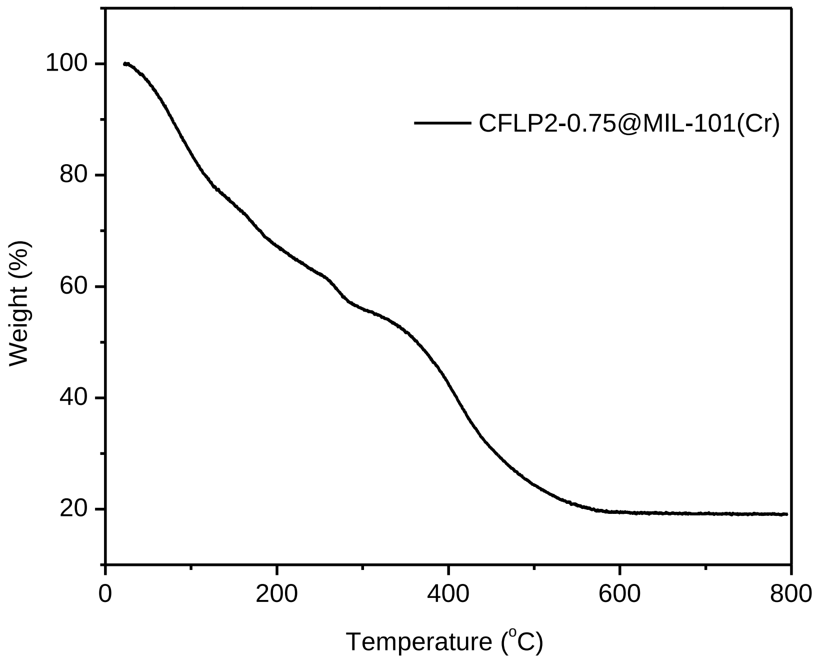

Figure S12. TGA curve of CFLP2-0.75@MIL-101(Cr).

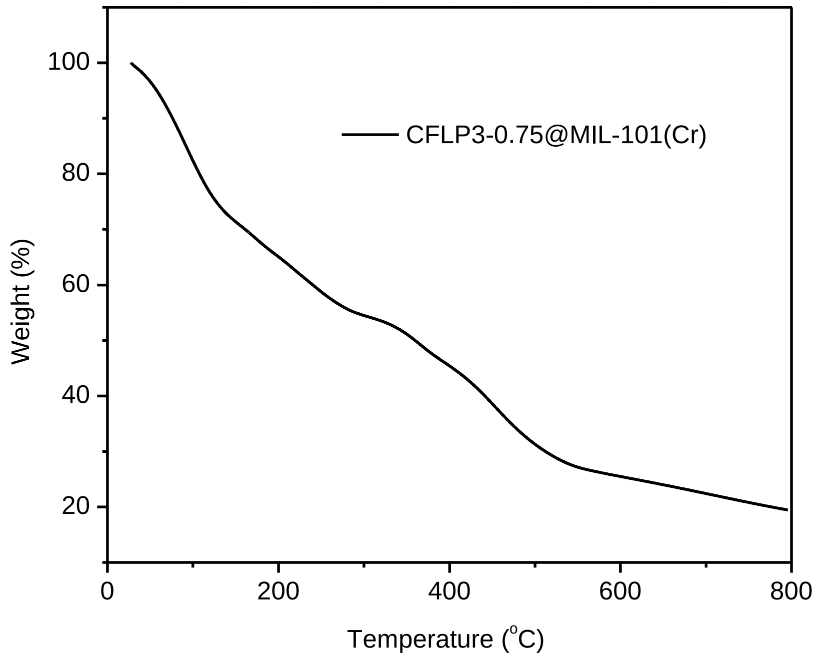

Figure S13. TGA curve of CFLP3-0.75@MIL-101(Cr).

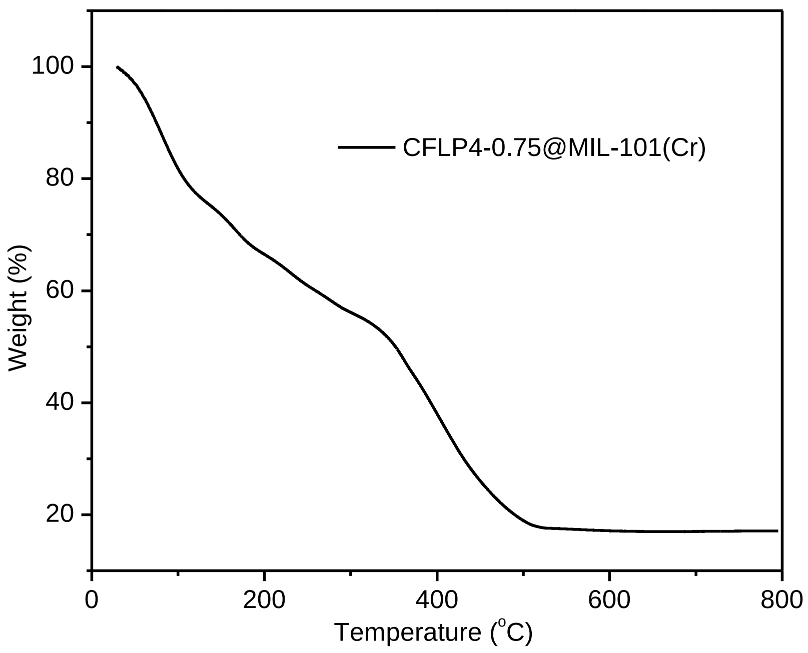

Figure S14. TGA curve of CFLP4-0.75@MIL-101(Cr).

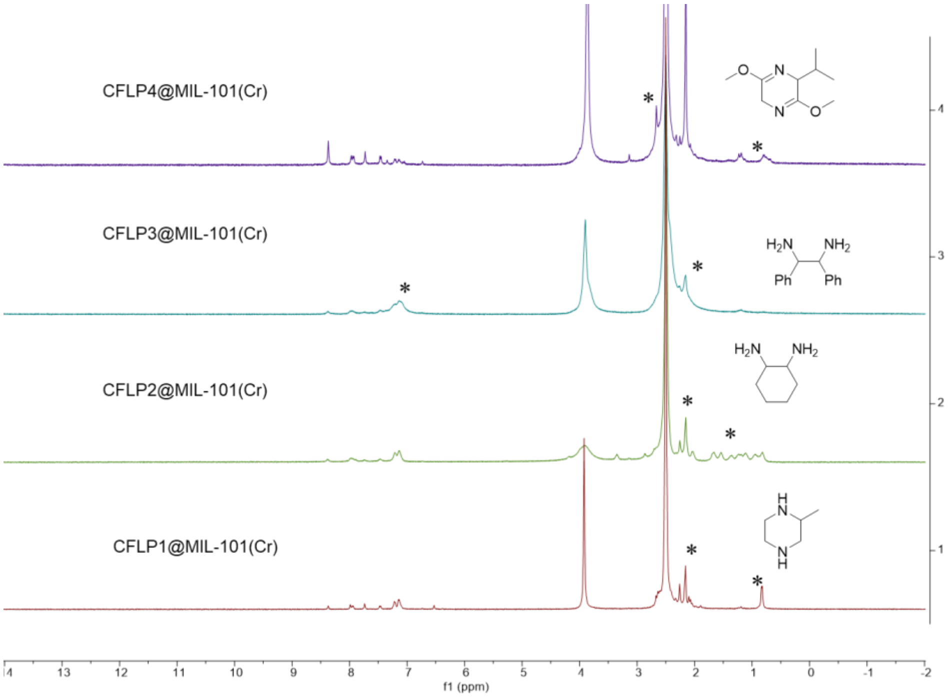

Figure S15. 1H NMR spectrum of digested CFLP@MIL-101(Cr) catalysts. Digestion conditions: 4 mg solid was dispersed in 0.2 mL NaOD (30 wt% in D_2O) solution. This mixture was sonicated for 30 minutes before adding 0.4 mL DMSO-d6. The resulting suspension was further sonicated for 30 minutes before the NMR test. The framework of MIL-101(Cr) remained partially insoluble even after sonicating for a long time.

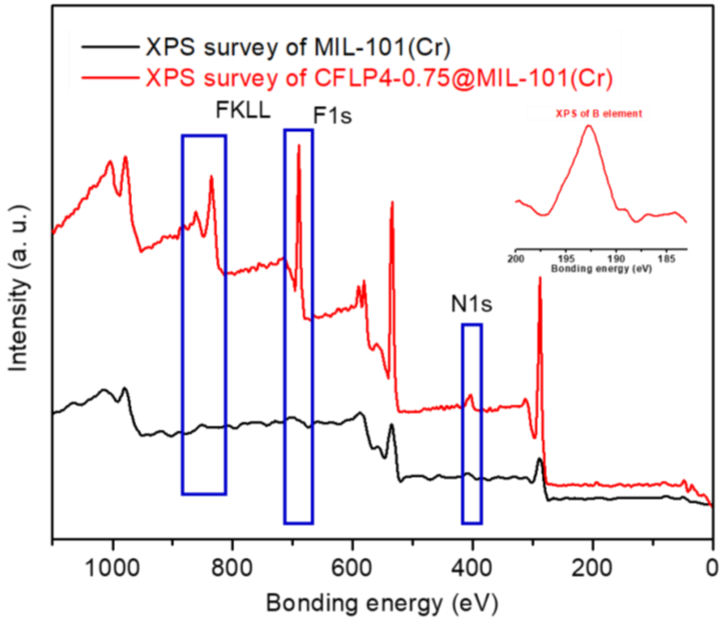

Figure S16. XPS survey of CFLP4-0.75@MIL-101(Cr) and MIL-101(Cr), inset is the B signal of CFLP4-0.75@MIL-101(Cr).

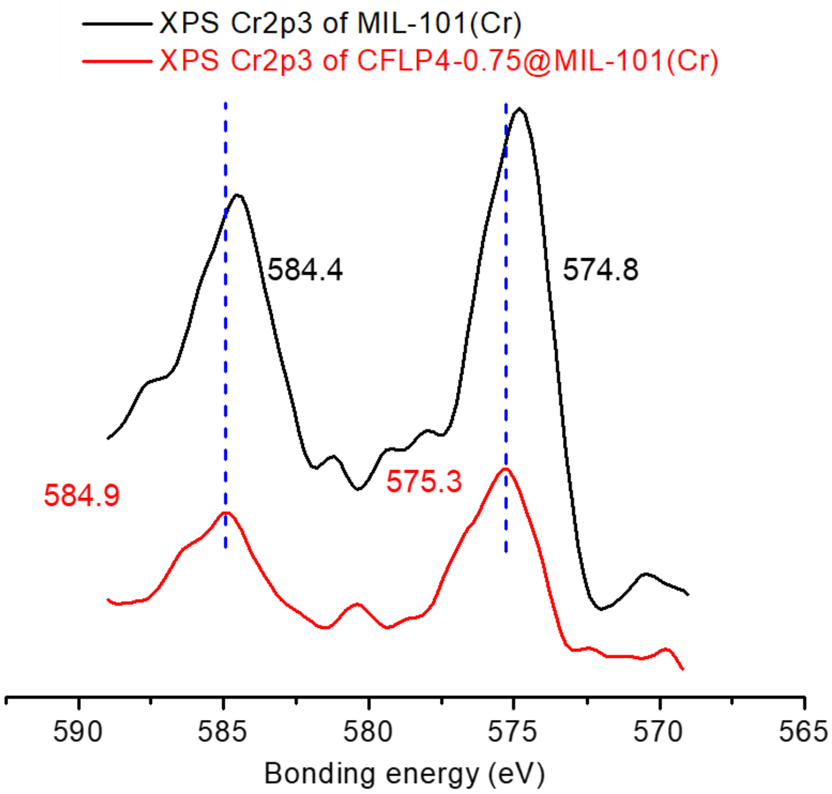

Figure S17. XPS Cr2p spectra of CFLP4-0.75@MIL-101(Cr) and MIL-101(Cr).

Table S2. Product distribution in catalytic tests.

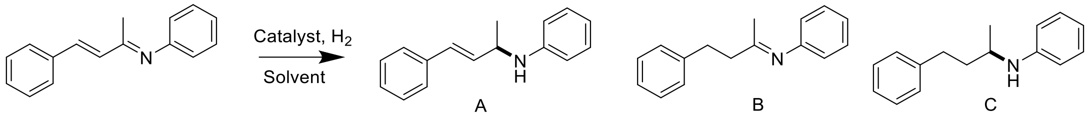

Entry	Catalyst	Solvent	A (%)	B (%)	C (%)
1[a]	MIL-101(Cr)	Toluene	0	0	0
2	CFLP4	Toluene	50	46	nt
3	CFLP4-0.75@MIL-101(Cr)	Toluene	95	nt	nt
4	MIL-101(Cr) + CFLP4	Toluene	57	27	16
5[b]	CFLP4-0.75@MIL-101(Cr)	Toluene	94	nd	nt
6[c]	CFLP4-0.75(BCF)@MIL-101(Cr)	Toluene	50	14	36 (76% ee)
7	CFLP4-0.5@MIL-101(Cr)	Toluene	81	nt	nt
8	CFLP4-1.0@MIL-101(Cr)	Toluene	76	16	8
9	CFLP4-0.75@MIL-101(Cr)	CH_2Cl_2	62	9	14
10	CFLP4-0.75@MIL-101(Cr)	CH_3CN	97	nt	nt
11	CFLP1-0.75@MIL-101(Cr)	Toluene	76	nt	18
12	CFLP2-0.75@MIL-101(Cr)	Toluene	81	nt	16
13	CFLP3-0.75@MIL-101(Cr)	Toluene	87	nt	12

nt = not tested, because of low amount.

Table S3. Substrate selectivity investigation.

Catalyst	Substrate	Product	Yield (%)
CFLP4-0.75@MIL-101(Cr)	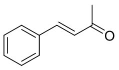	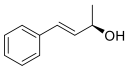	0
CFLP4-0.75@MIL-101(Cr)	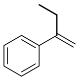	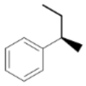	0
CFLP4-0.75@MIL-101(Cr)	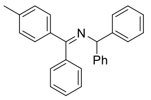	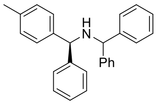	0

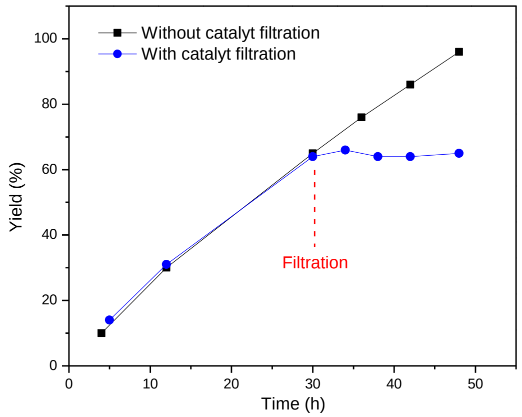

Figure S18. Leaching test for the asymmetric hydrogenation with CFLP4-0.75@MIL-101(Cr), namely yield versus reaction time with and without catalyst filtration.

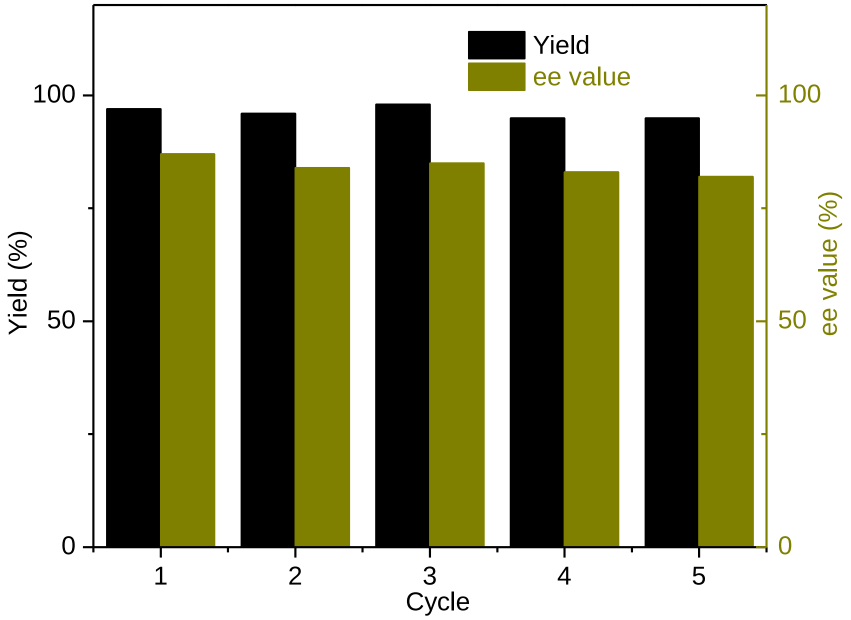

Figure S19. Catalytic performance of CFLP4-0.75@MIL-101(Cr) in 5-run recycle tests.

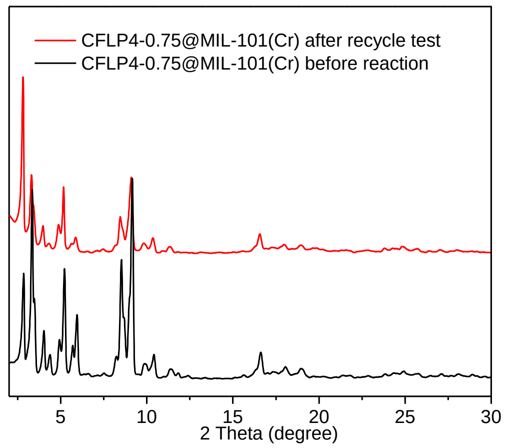

Figure S20. PXRD patterns of CFLP4-0.75@MIL-101(Cr) before (black curve) and after (red curve) the recycle test.

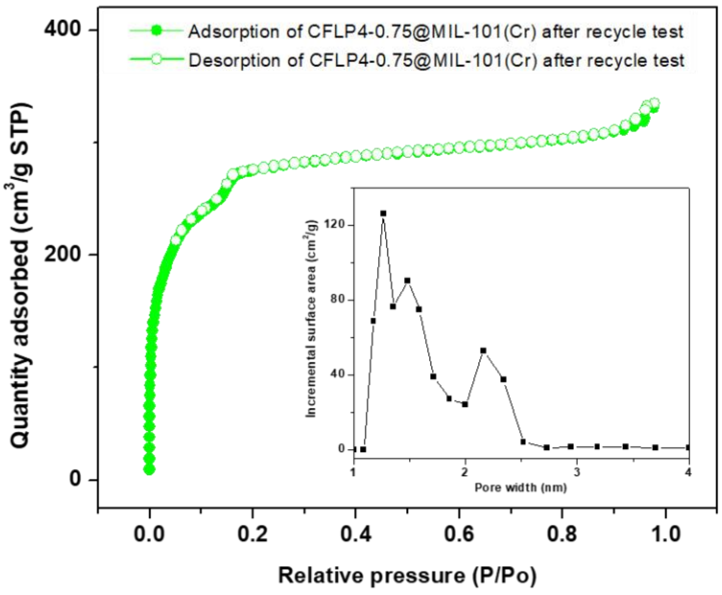

Figure S21. N_2 sorption test and pore size distribution of CFLP4-0.75@MIL-101(Cr) after the recycle test.

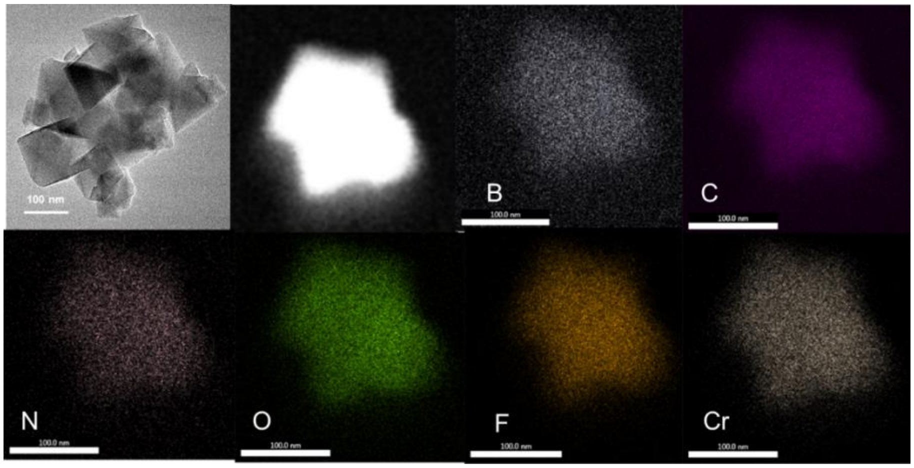

Figure S22. Transmission electron microscopy image and elemental mapping of CFLP4-0.75@MIL-101(Cr) after the recycle test, scale bar 100 nm.

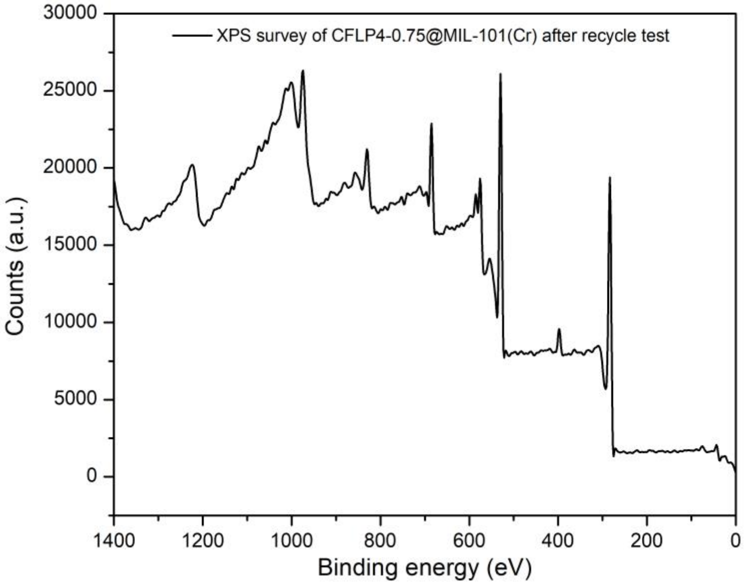

Figure S23. XPS survey of CFLP4-0.75@MIL-101(Cr) after the recycle test.

Table S4. Contrast of CFLP4-0.75@MIL-101(Cr) with variation of open Cr(III) site and hydroxyl group.

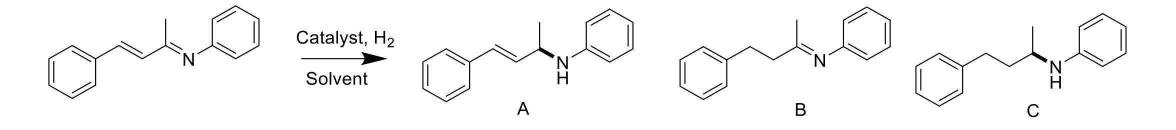

Entry	Catalyst	Solvent	A (%)	B (%)	C (%)
1	CFLP4-0.75@MIL-101(Cr)	Toluene	95	nt	nt
2	CFLP4-0.75@MIL-101(Cr)-OH	Toluene	94	nt	nt
3	CFLP4-0.75@MIL-101(Cr)-Cr	Toluene	95	nt	nt
4	CFLP4-0.75@MIL-101(Cr)-None	Toluene	40	42	18

CFLP4-0.75@MIL-101(Cr)-OH denotes CFLP4-0.75@MIL-101(Cr) with only hydroxyl group and it was prepared by dispersing CFLP4-0.75@MIL-101(Cr) in CH_3CN that blocks open Cr sites. CFLP4-0.75@MIL-101(Cr)-Cr refers to CFLP4-0.75@MIL-101(Cr) with only open Cr sites and it was prepared by exchanging hydroxyl groups with Br (PBr_3/THF). CFLP4-0.75@MIL-101(Cr)-None means CFLP4-0.75@MIL-101(Cr) without open Cr sites and hydroxyl groups and it was prepared by a sequential blocking of open Cr sites and exchange of hydroxyls groups with Br. nt = not tested, because of low amount.

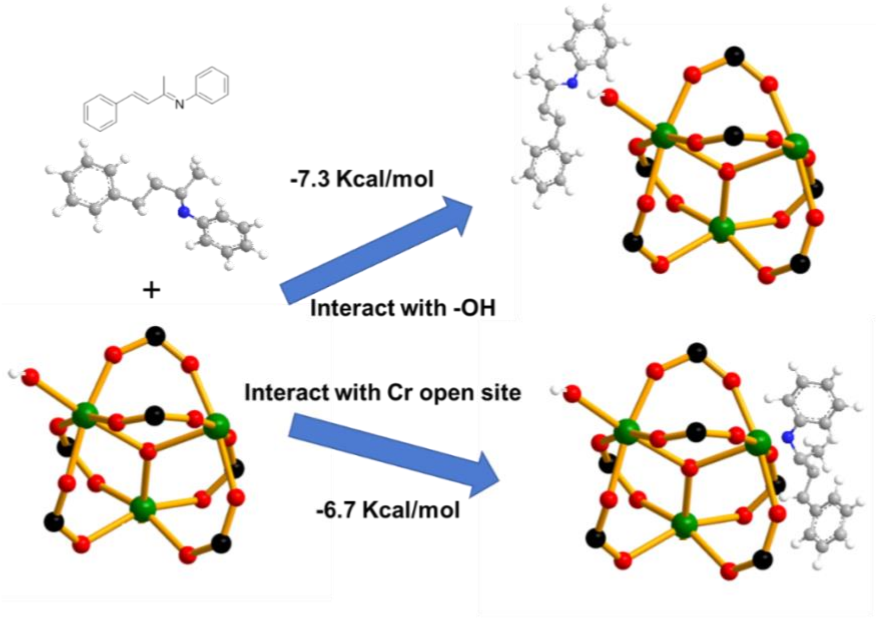

Figure S24. The adsorption energy of substrate over hydroxyl group and open Cr site of Cr trimer with C=N. The color balls in the structures stand for black/gray = C, blue = N, red = O, white = H, and green = Cr.

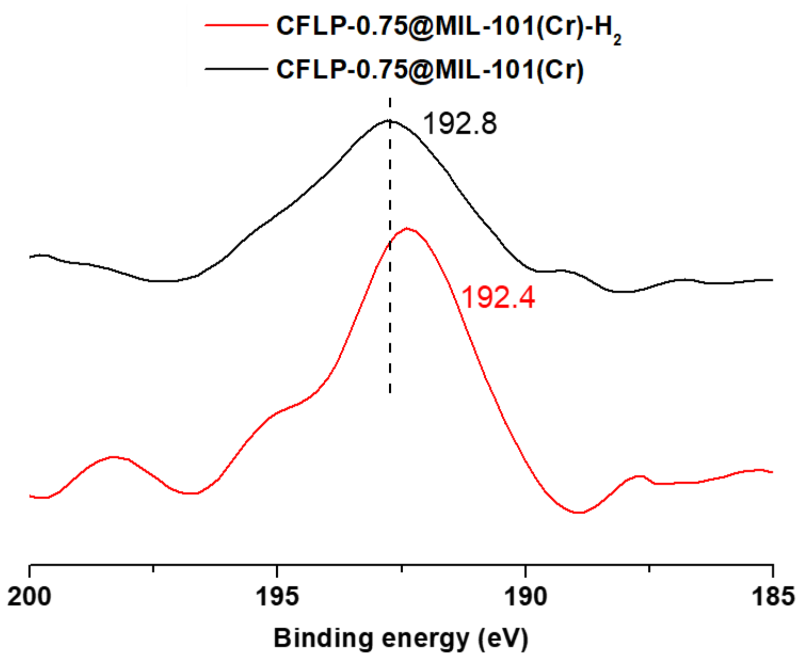

Figure S25. XPS B1s spectra of CFLP4-0.75@MIL-101(Cr) and CFLP4-0.75@MIL-101(Cr)-H_2.

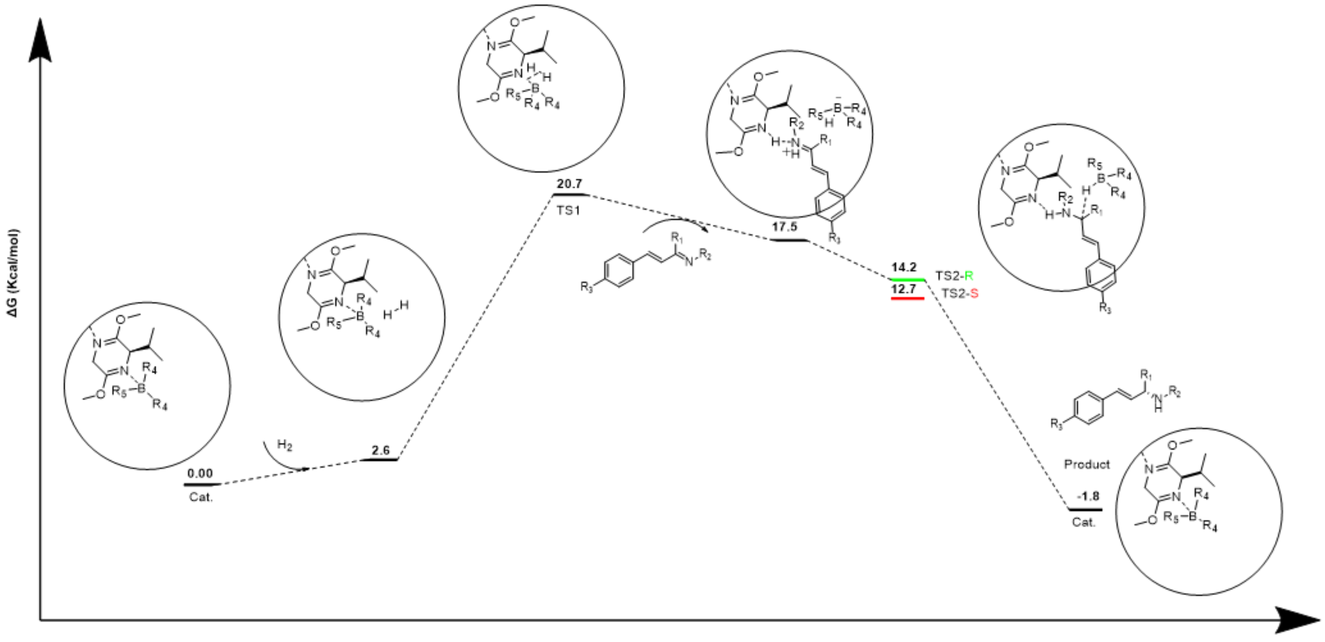

Figure S26. The energy profile of hydrogenation with CFLP4-0.75R@MIL-101(Cr).

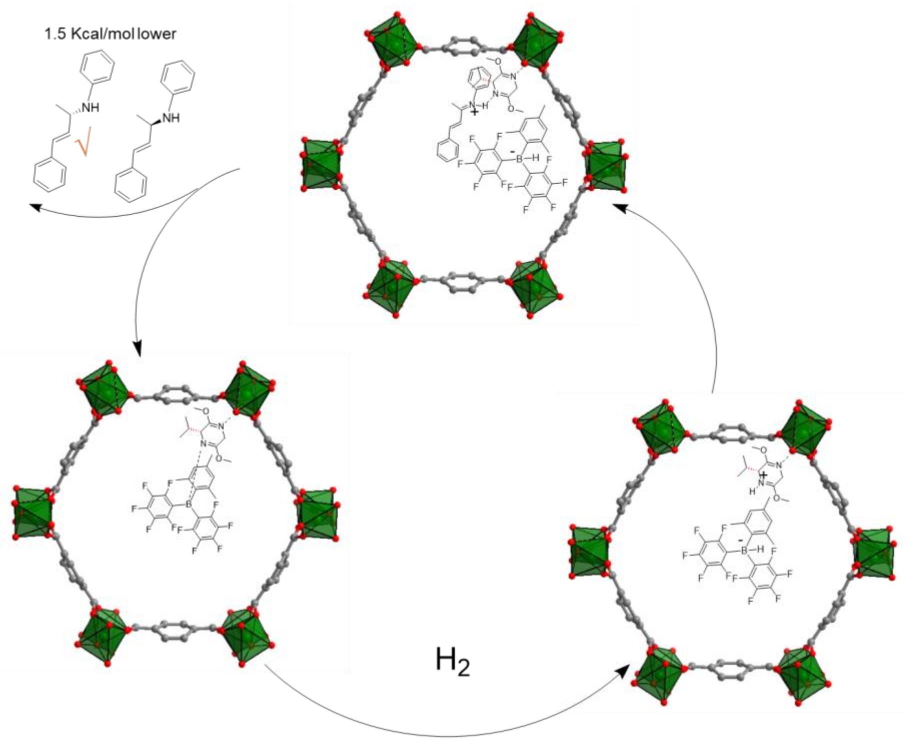

Scheme S2. Proposed mechanism diagram for asymmetric hydrogenation with FLP4@MIL-101(Cr).

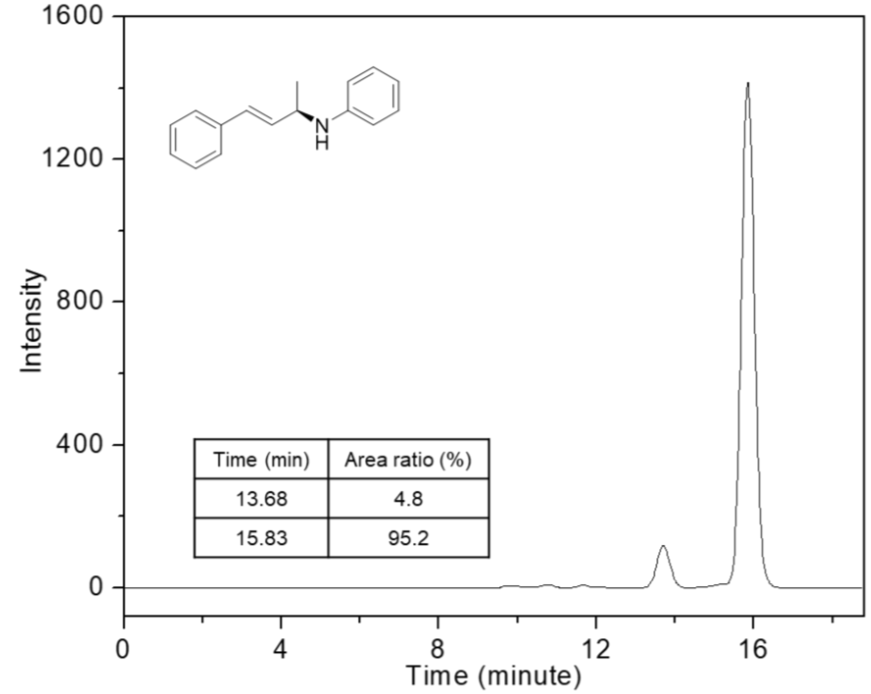

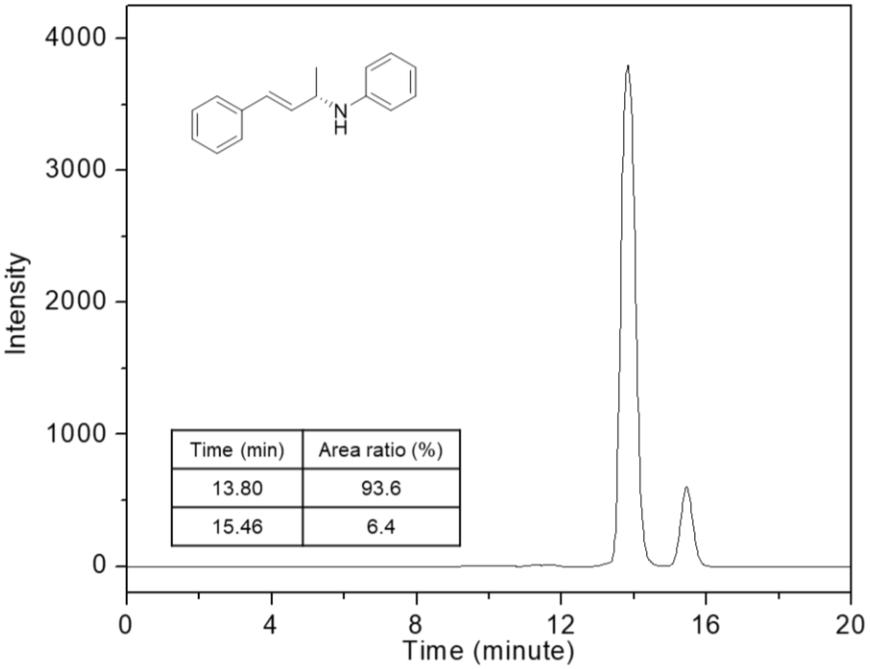

Time (min)	Area ratio (%)
13.80	93.6
15.46	6.4

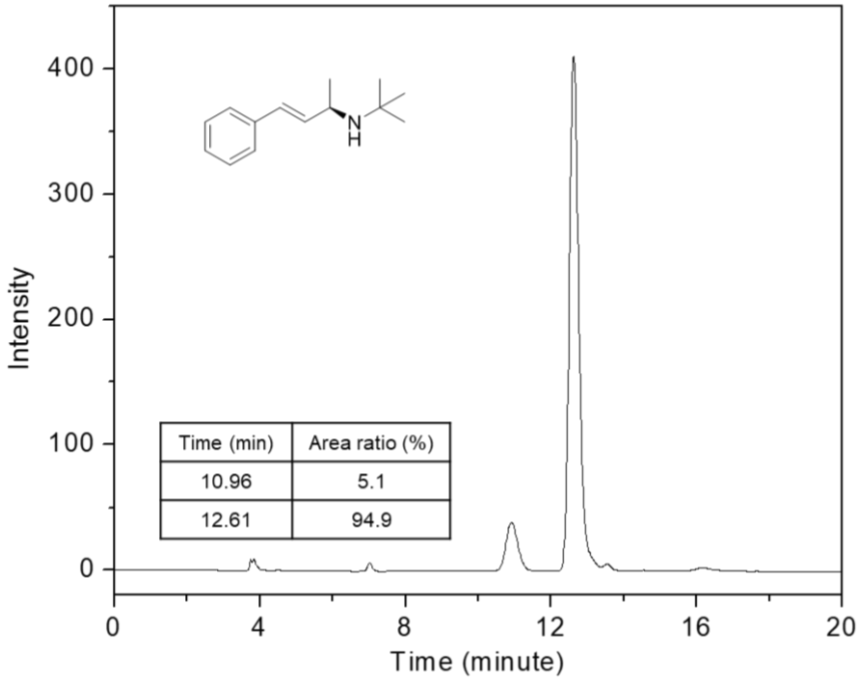

Time (min)	Area ratio (%)
10.96	5.1
12.61	94.9

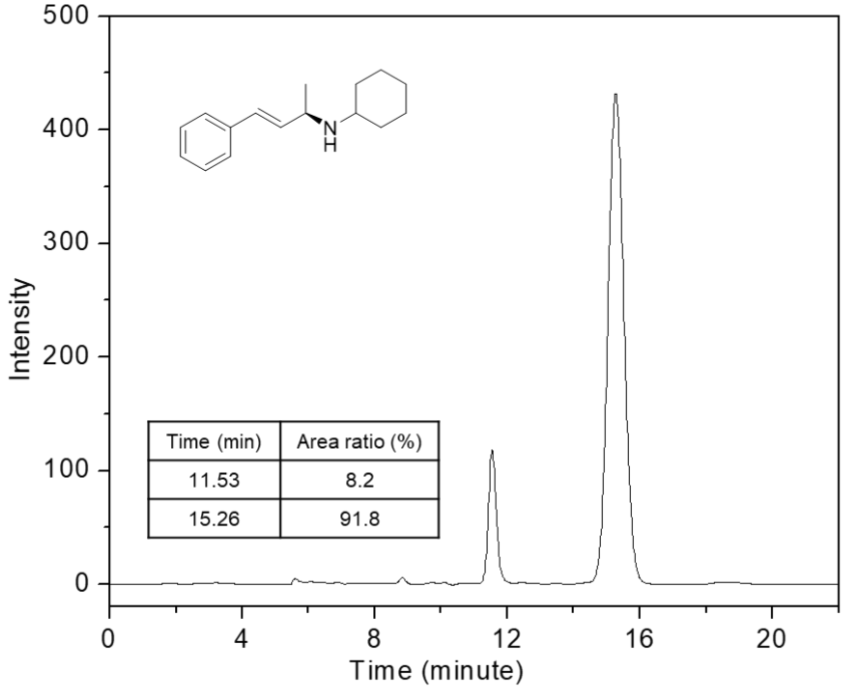
N
H
Time (min)
Area ratio (%)
11.53
8.2
15.26
91.8
Intensity
Time (minute)
500
400
300
200
100
0
0
4
8
12
16
20

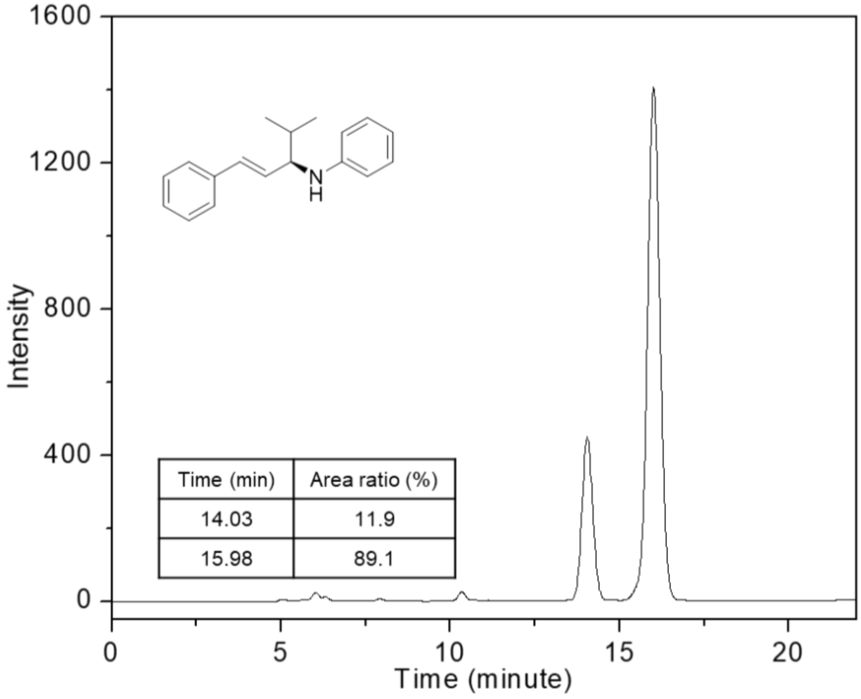
N
H
Time (min)
Area ratio (%)
14.03
11.9
15.98
89.1
Intensity
Time (minute)
1600
1200
800
400
0
0
5
10
15
20

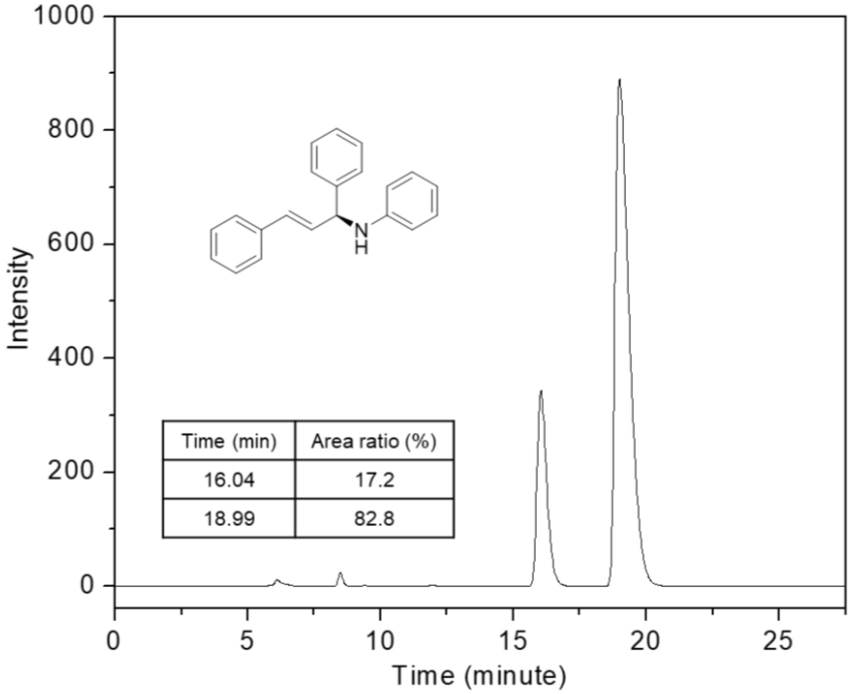
1000
800
600
400
200
0
Intensity
0
5
10
15
20
25
Time (minute)
N
H
Time (min)
Area ratio (%)
16.04
17.2
18.99
82.8

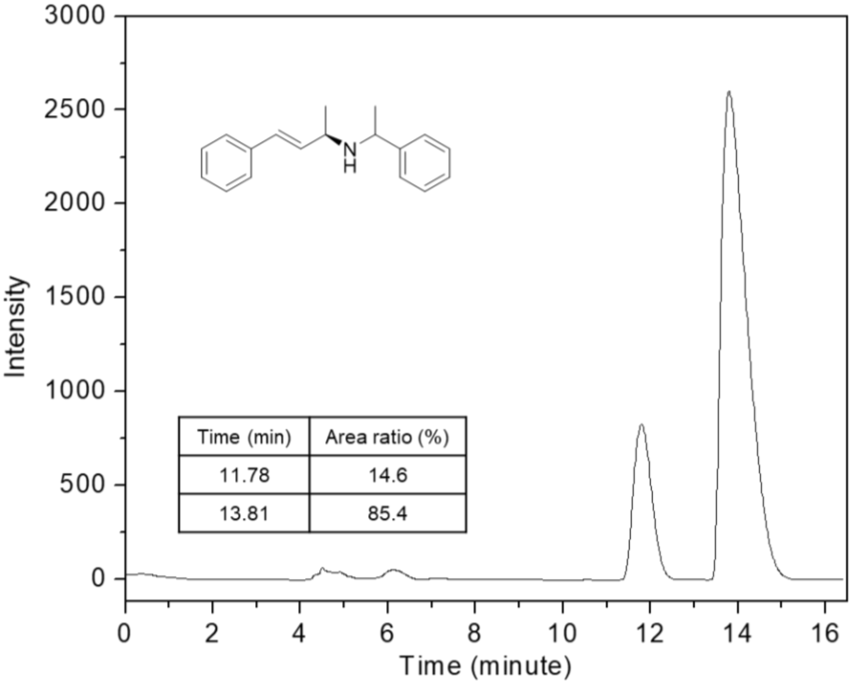
3000
2500
2000
1500
1000
500
0
Intensity
0
2
4
6
8
10
12
14
16
Time (minute)
N
H
Time (min)
Area ratio (%)
11.78
14.6
13.81
85.4

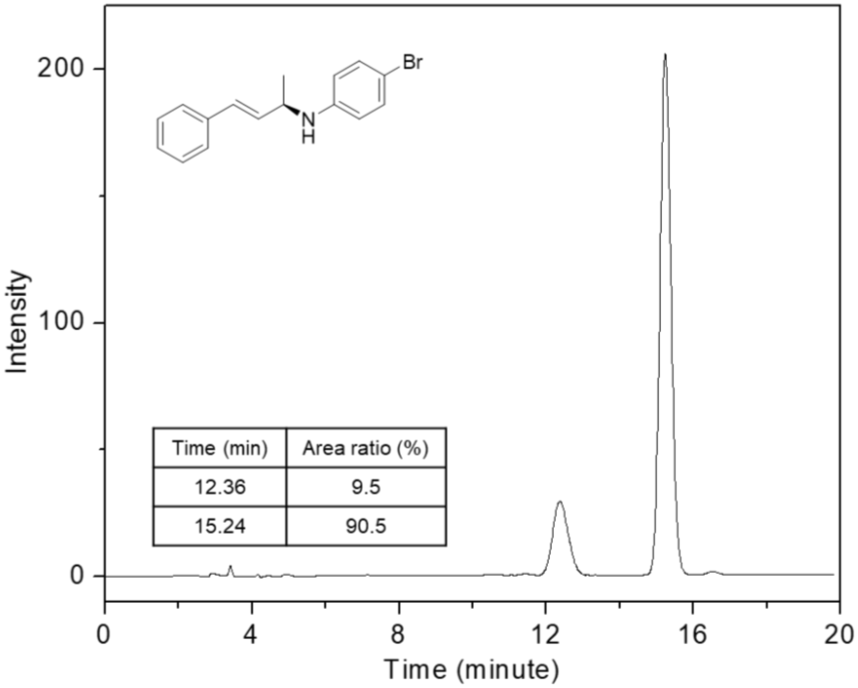
Br
N
H
Intensity
200
100
0
Time (min)
Area ratio (%)
12.36
9.5
15.24
90.5
0
4
8
12
16
20
Time (minute)

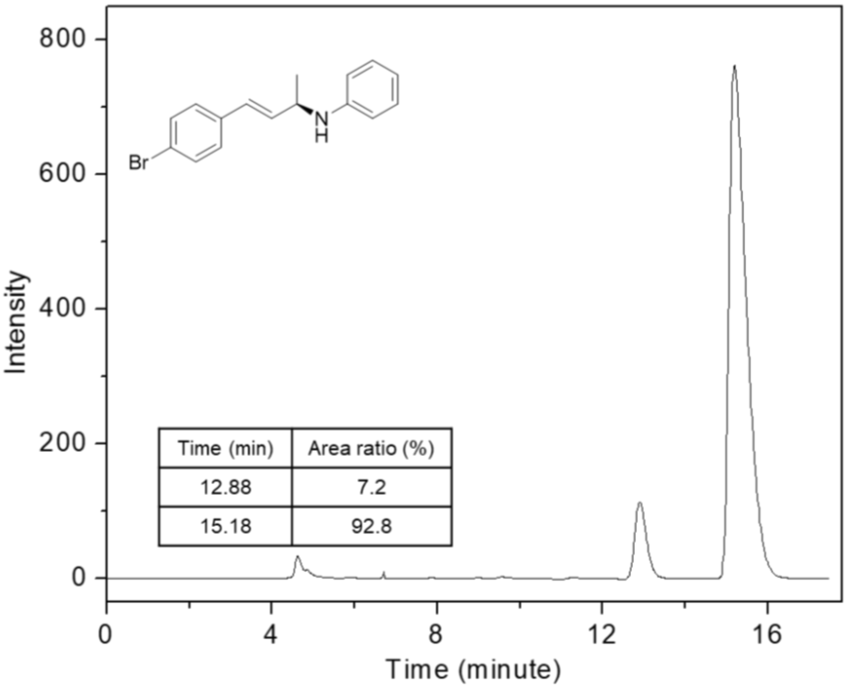
Br
N
H
Intensity
800
600
400
200
0
Time (min)
Area ratio (%)
12.88
7.2
15.18
92.8
0
4
8
12
16
Time (minute)

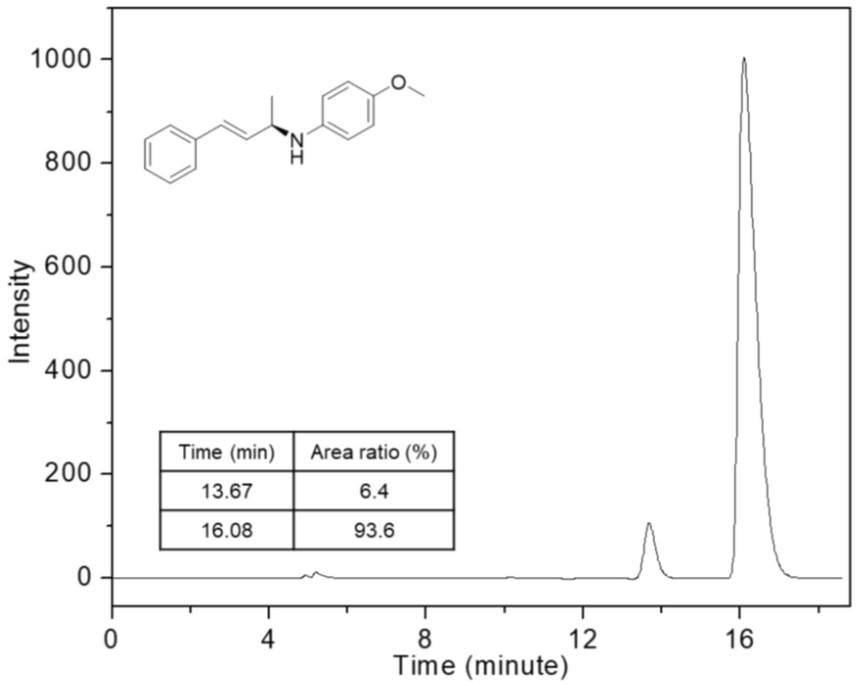

1000
800
600
400
200
0
Intensity
N
H
O
Time (min)
Area ratio (%)
13.67
6.4
16.08
93.6
0
4
8
12
16
Time (minute)

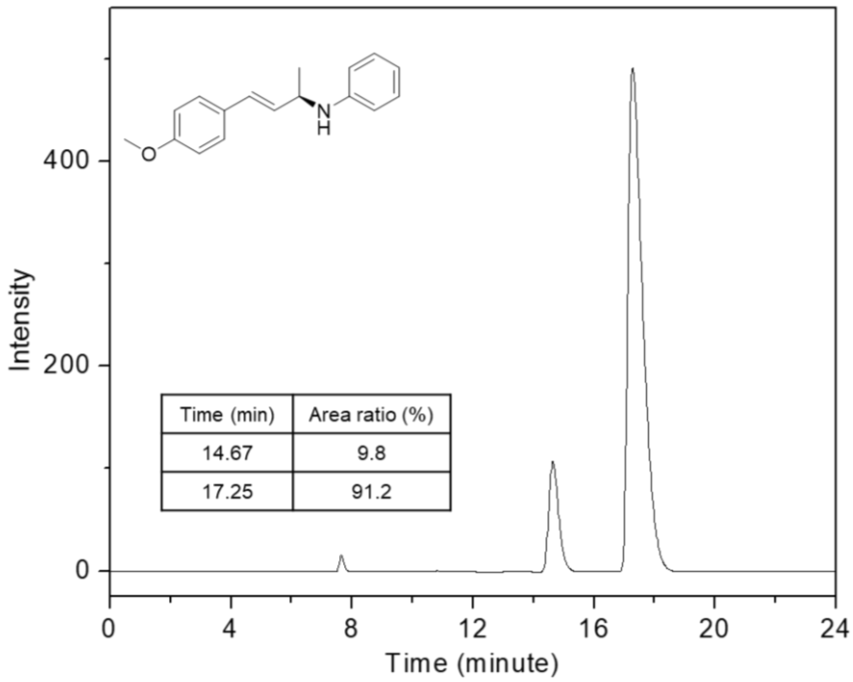

400
200
0
Intensity
O
N
H
Time (min)
Area ratio (%)
14.67
9.8
17.25
91.2
0
4
8
12
16
20
24
Time (minute)

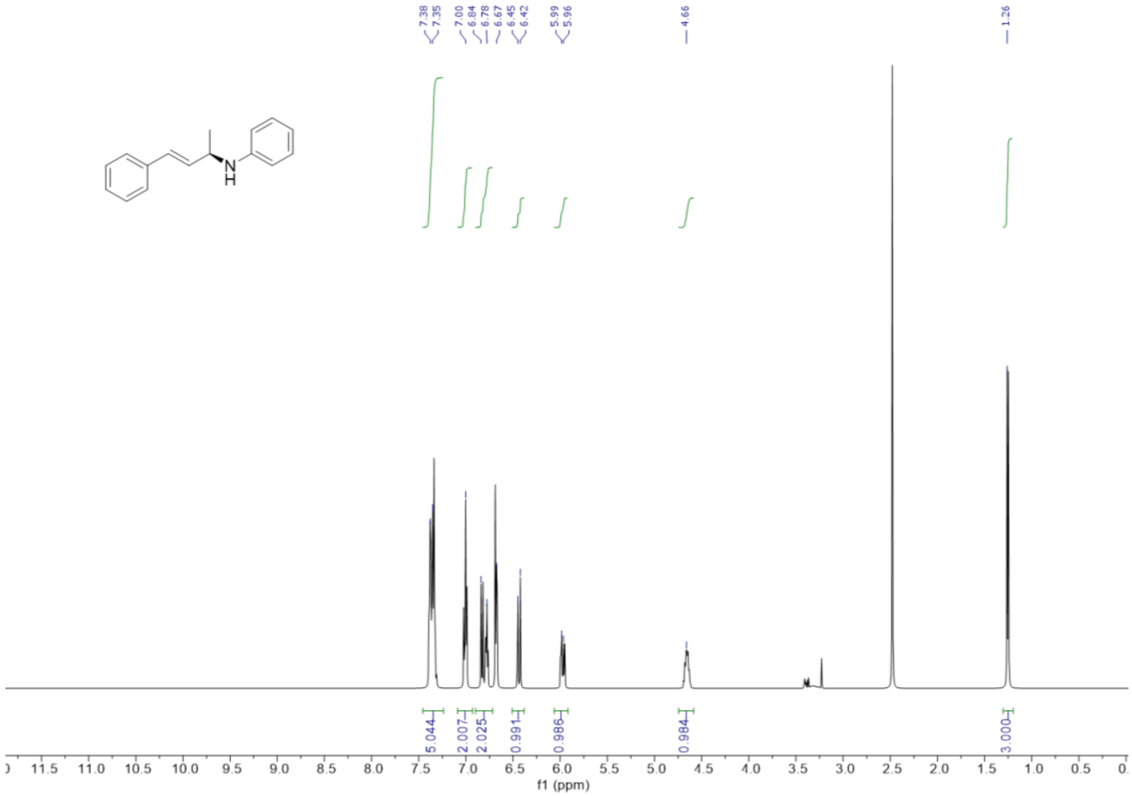
7.38
7.35
7.00
6.84
6.78
6.67
6.45
6.42
5.99
5.96
4.66
1.26
N
H
5.044
2.007
2.025
0.991
0.986
0.984
3.000
f1 (ppm)

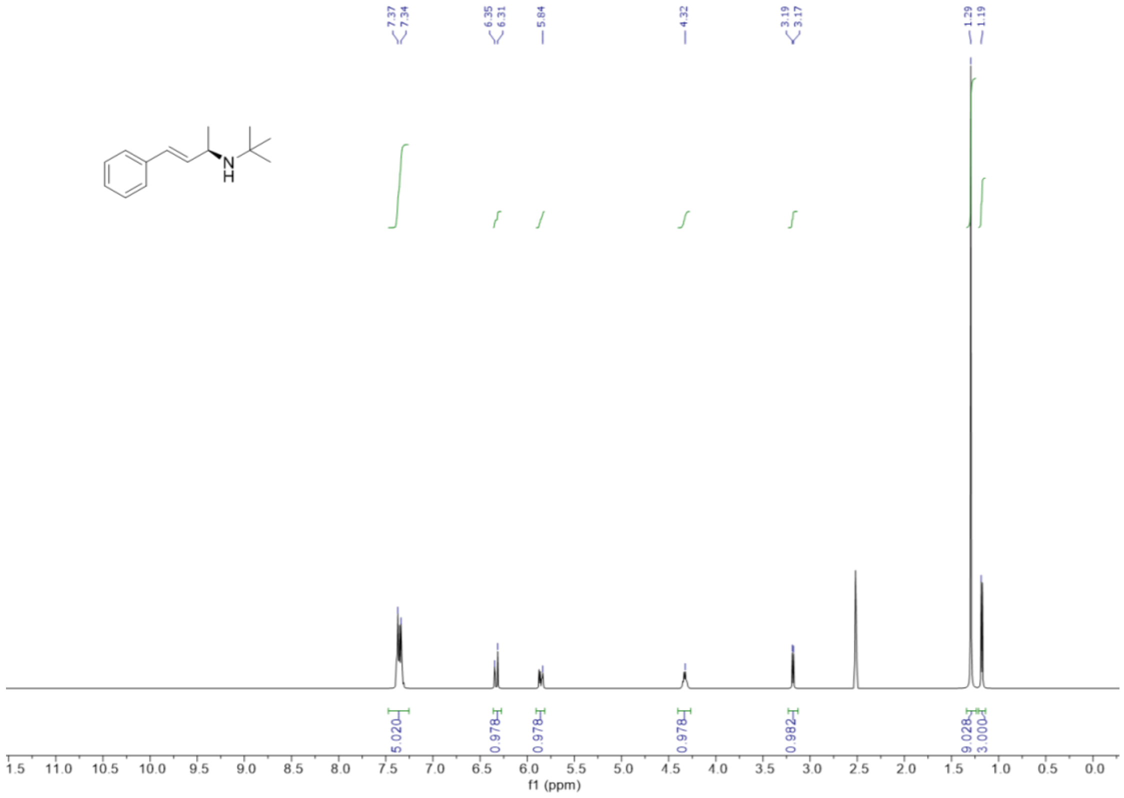
7.37
7.34
6.35
6.31
5.84
4.32
3.19
3.17
1.29
1.19
N
H
5.020
0.978
0.978
0.978
0.982
9.028
3.000
f1 (ppm)

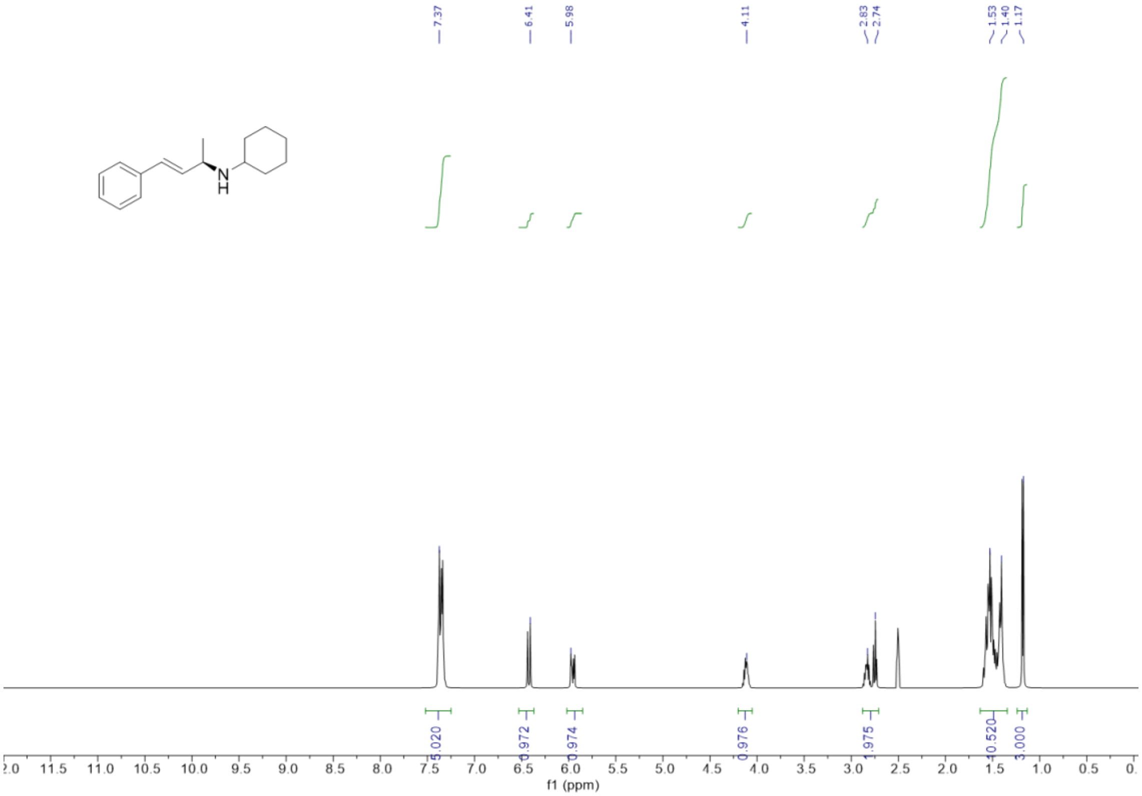
N
H
7.37
6.41
5.98
4.11
2.83
2.74
1.53
1.40
1.17
5.020
0.972
0.974
0.976
1.975
10.520
3.000
f1 (ppm)

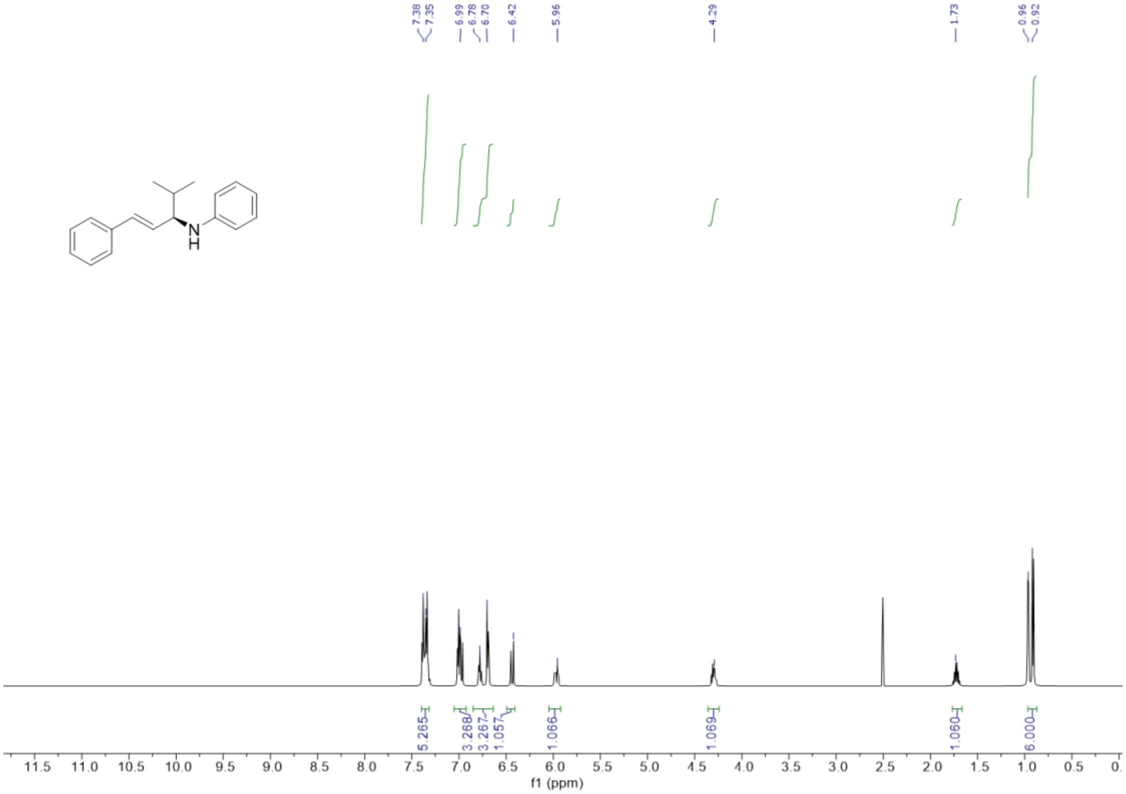
N
H
7.38
7.35
6.99
6.78
6.70
6.42
5.96
4.29
1.73
0.96
0.92
5.265
3.268
3.267
1.057
1.066
1.069
1.060
6.000
f1 (ppm)

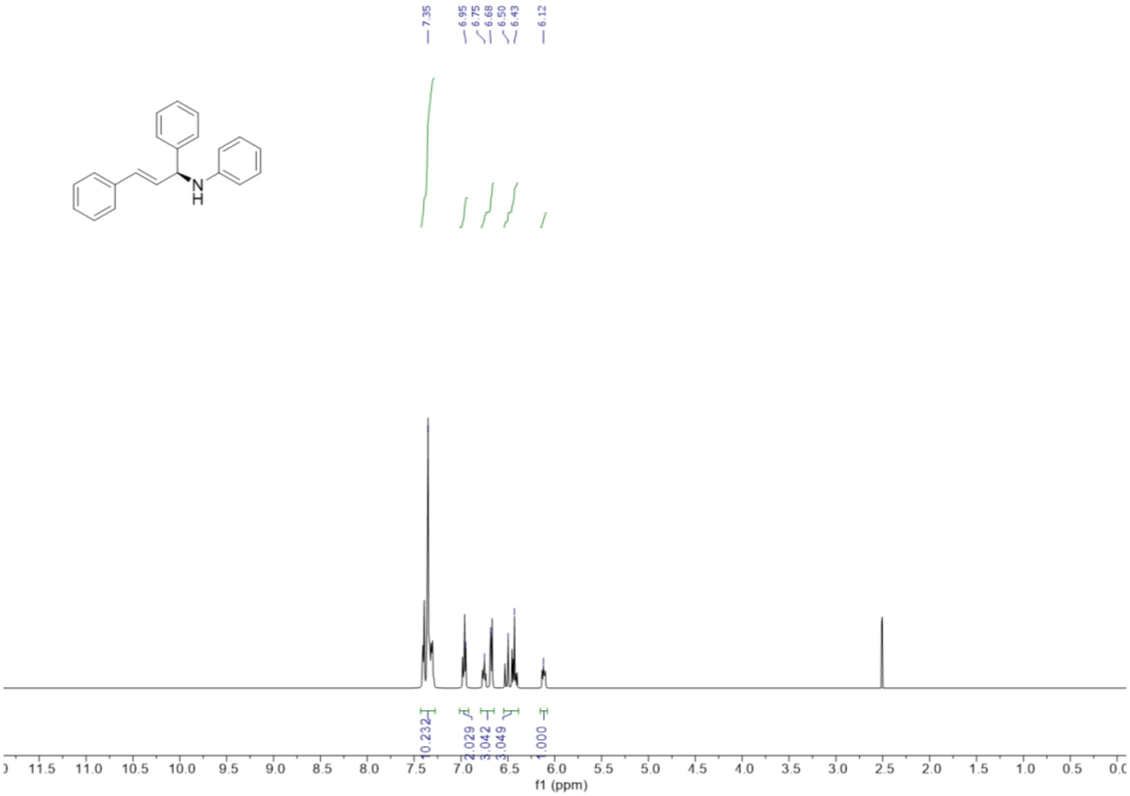
7.35
6.95
6.75
6.68
6.50
6.43
6.12
N
H
10.232
2.029
3.042
3.049
1.000
f1 (ppm)

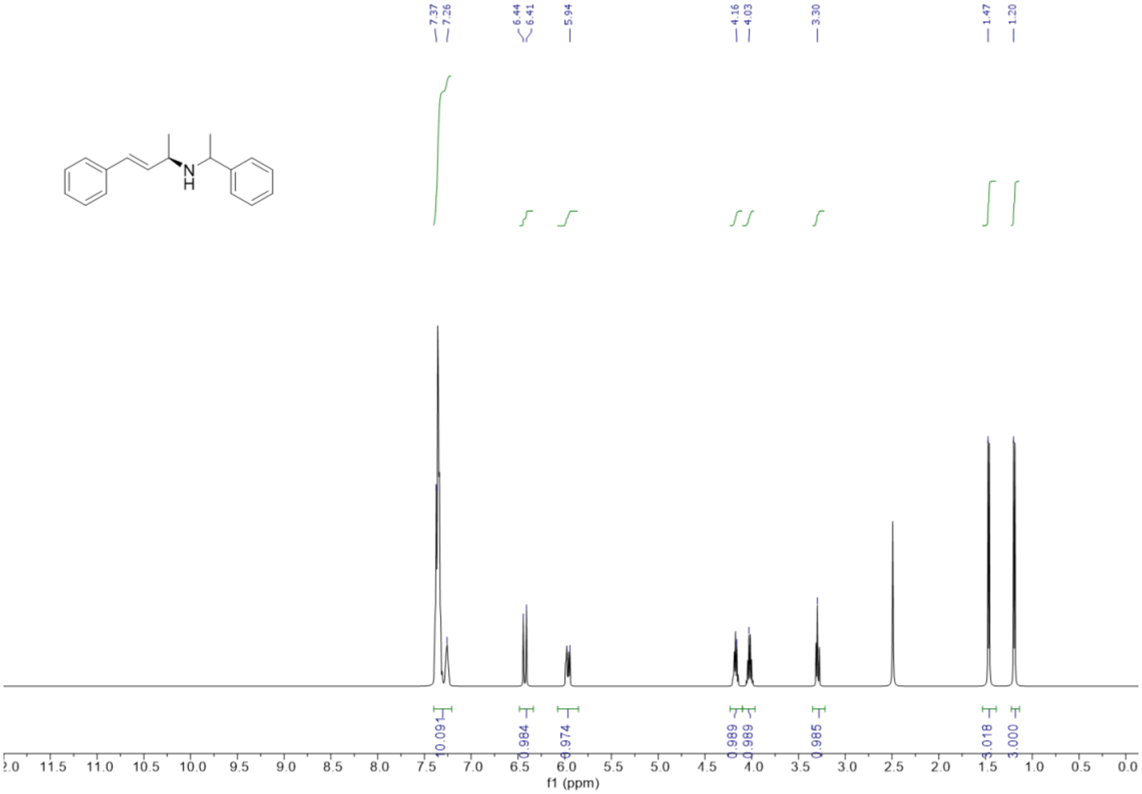
7.37
7.26
6.44
6.41
5.94
4.16
4.03
3.30
1.47
1.20
N
H
10.091
0.984
0.974
0.989
0.989
0.985
3.018
3.000
f1 (ppm)

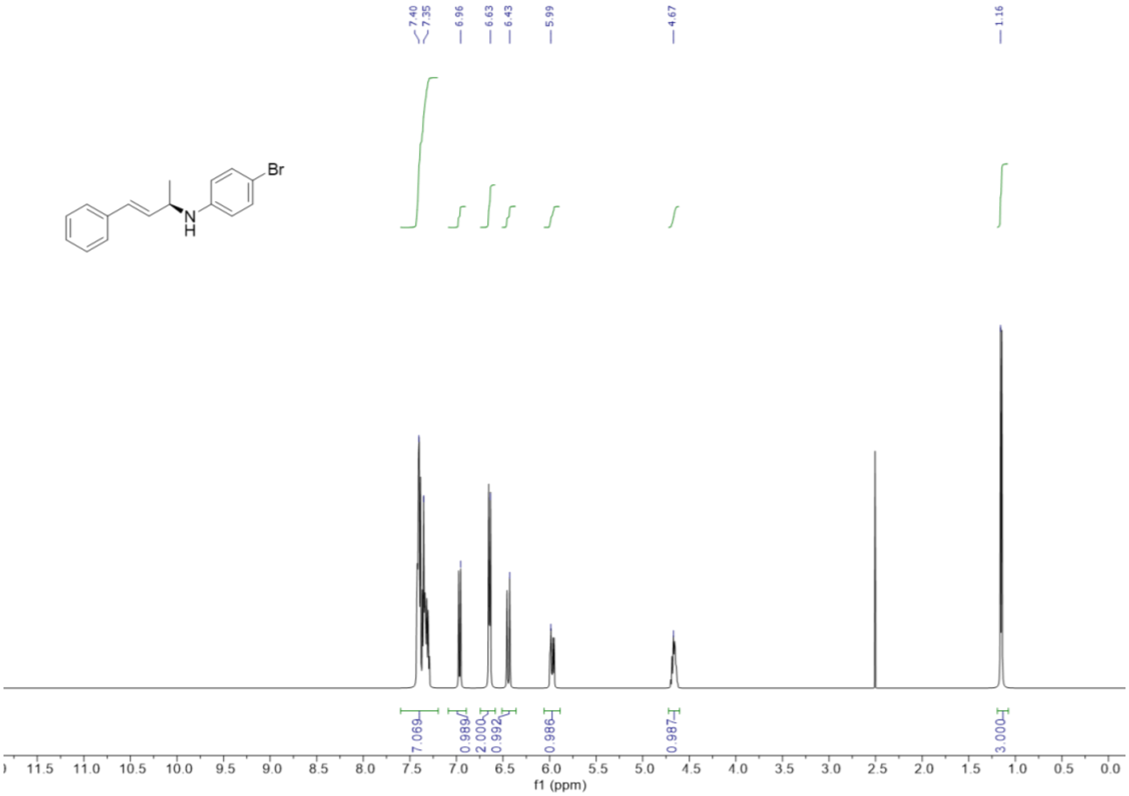
Br
N
H
7.40
7.35
6.96
6.63
6.43
5.99
4.67
1.16
7.069
0.989
2.000
0.992
0.986
0.987
3.000
f1 (ppm)

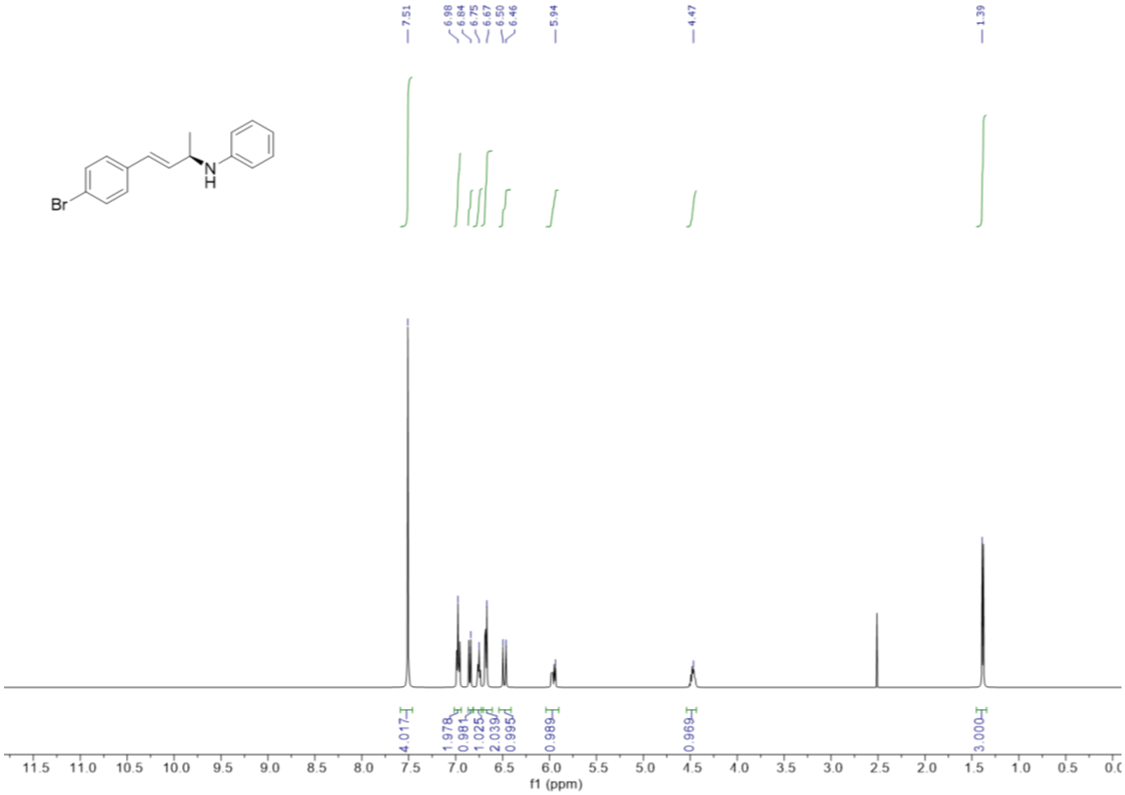
Br
N
H
7.51
6.98
6.84
6.75
6.67
6.50
6.46
5.94
4.47
1.39
4.017
1.978
0.981
1.025
2.039
0.995
0.989
0.969
3.000
f1 (ppm)

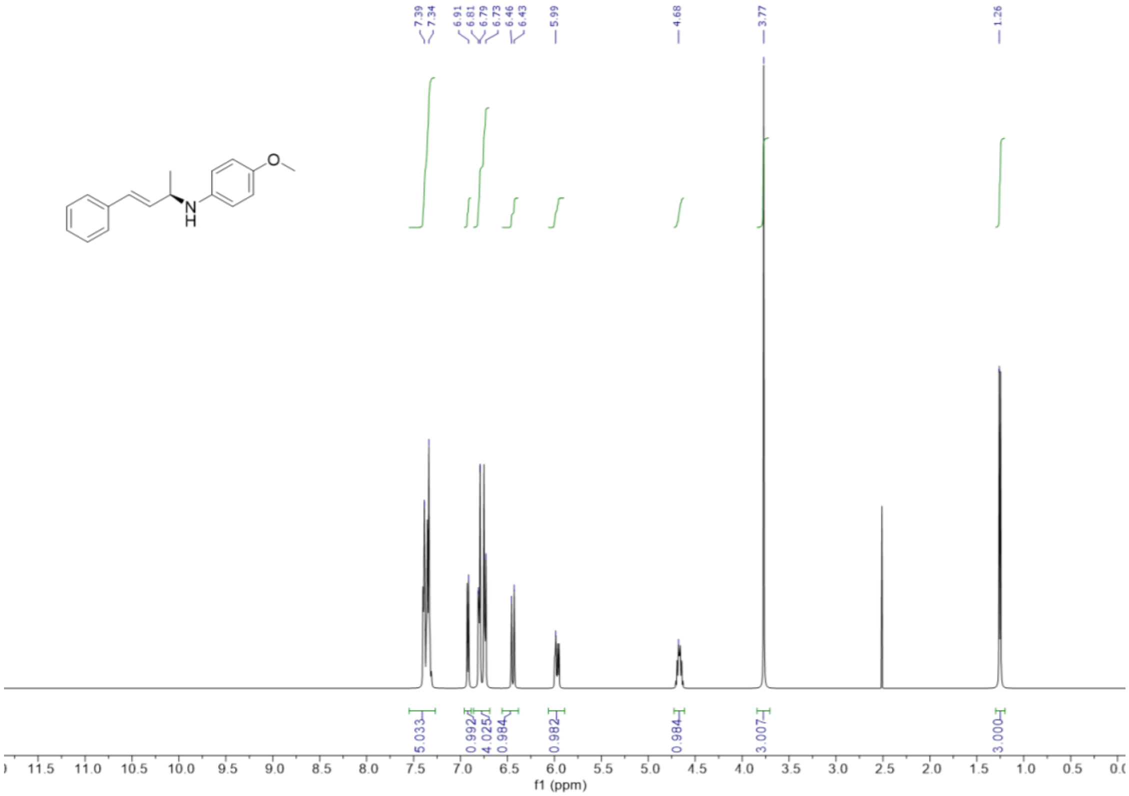
7.39
7.34
6.91
6.81
6.79
6.73
6.46
6.43
5.99
4.68
3.77
1.26
5.033
0.992
4.025
0.984
0.982
0.984
3.007
3.000
f1 (ppm)

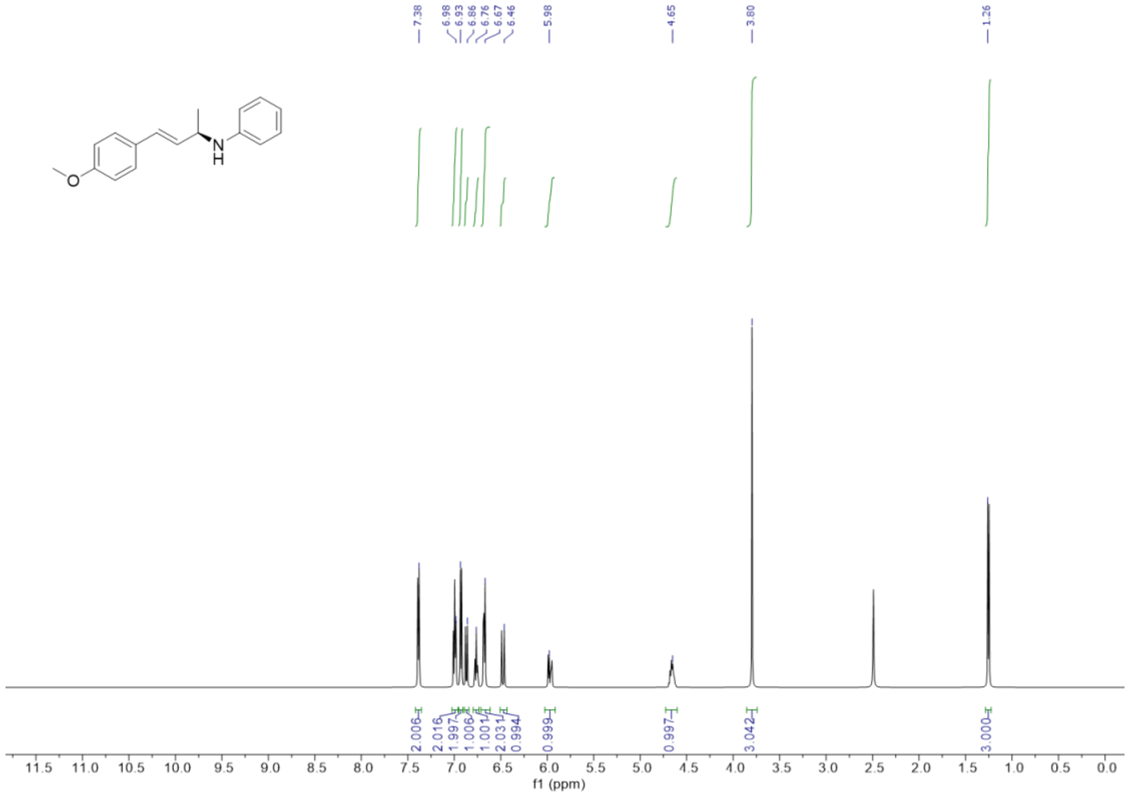
7.38
6.98
6.93
6.86
6.76
6.67
6.46
5.98
4.65
3.80
1.26
2.006
2.016
1.997
1.006
1.001
2.031
0.994
0.999
0.997
3.042
3.000
f1 (ppm)

References

(1) Frisch, M. J.; Trucks, G. W.; Schlegel, H. B.; Scuseria, G. E.; Robb, M. A.; Cheeseman, J. R.; Scalmani, G.; Barone, V.; Petersson, G. A.; Nakatsuji, H.; Li, X.; Caricato, M.; Marenich, A. V.; Bloino, J.; Janesko, B. G.; Gomperts, R.; Mennucci, B.; Hratchian, H. P.; Ortiz, J. V.; Izmaylov, A. F.; Sonnenberg, J. L.; Williams-Young, D.; Ding, F.; Lipparini, F.; Egidi, F.; Goings, J.; Peng, B.; Petrone, A.; Henderson, T.; Ranasinghe, D.; Zakrzewski, V. G.; Gao, J.; Rega, N.; Zheng, G.; Liang, W.; Hada, M.; Ehara, M.; Toyota, K.; Fukuda, R.; Hasegawa, J.; Ishida, M.; Nakajima, T.; Honda, Y.; Kitao, O.; Nakai, H.; Vreven, T.; Throssell, K.; Montgomery, J. A. Jr.; Peralta, J. E.; Ogliaro, F.; Bearpark, M. J.; Heyd, J. J.; Brothers, E. N.; Kudin, K. N.; Staroverov, V. N.; Keith, T. A.; Kobayashi, R.; Normand, J.; Raghavachari, K.; Rendell, A. P.; Burant, J. C.; Iyengar, S. S.; Tomasi, J.; Cossi, M.; Millam, J. M.; Klene, M.; Adamo, C.; Cammi, R.; Ochterski, J. W.; Martin, R. L.; Morokuma, K.; Farkas, O.; Foresman, J. B.; Fox, D. J. Gaussian, Inc., Wallingford CT, 2016.

(2) Zhao Y.; Truhlar D.-G. The M06 Suite of Density Functionals for Main Group Thermochemistry, Thermochemical Kinetics, Noncovalent Interactions, Excited States, and Transition Elements: Two New Functionals and Systematic Testing of Four M06-class Functionals and 12 Other Functionals. *Theor. Chem. Acc.* **2008**, *120*, 215-241.

(3) Marenich, A. V.; Cramer, C. J.; Truhlar, D.-G. Universal Solvation Model Based on Solute Electron Density and on a Continuum Model of the Solvent Defined by the Bulk Dielectric Constant and Atomic Surface Tensions. *J. Phys. Chem. B* **2009**, *113*, 6378-6396.

(4) Cummings, S. A.; Limura, M. C.; Harlan, C. J.; Kwaan, R. J.; Trieu, I. V.; Norton, J. R.; Bridgewater, B. M.; Jäkle, F.; Sundararaman, A.; Tilset, M. An Estimate of the Reduction Potential of $B(C_6F_5)_3$ from Electrochemical Measurements on Related Mesityl Boranes. *Organometallics* **2006**, *25*, 1565-1568.

(5) Aguila, B.; Sun, Q.; Wang, X.; O'Rouke, E.; Al-Enizi, A. M.; Nafady, A.; Ma, S. Lower Activation Energy for Catalytic Reactions through Host-Guest Cooperation within Metal-Organic Frameworks. *Angew. Chem. Int. Ed.* **2018**, *57*, 10107-10111.

(6) Niu, Z.; Zhang, W.; Lan, P. C.; Aguila, B.; Ma, S. Promoting Frustrated Lewis Pairs for Heterogeneous Chemoselective Hydrogenation via the Tailored Pore Environment within Metal-Organic Frameworks. *Angew. Chem. Int. Ed.* **2019**, *58*, 7420-7424.

Coordinate in the theoretical calculation

N1

6	0.251592000	1.236495000	0.155852000
6	-1.825254000	0.033817000	0.298565000
6	0.969390000	-0.017412000	-0.344720000
1	0.364359000	1.286248000	1.255053000
1	0.719296000	2.145987000	-0.251588000
6	-1.126842000	-1.223044000	-0.208490000
1	-1.815663000	0.033998000	1.402555000
1	-2.880336000	0.057857000	-0.010095000
1	0.917127000	-0.018272000	-1.450199000
1	-1.590829000	-2.131809000	0.201720000
1	-1.233147000	-1.279679000	-1.305754000
7	-1.174999000	1.262970000	-0.144089000

1	-1.298543000	1.356799000	-1.152419000
7	0.300717000	-1.244325000	0.094565000
1	0.423186000	-1.328201000	1.106161000
6	2.420625000	-0.045643000	0.086980000
1	2.915008000	-0.955983000	-0.271502000
1	2.967771000	0.827271000	-0.291969000
1	2.494680000	-0.030005000	1.185588000

N2

7	1.762653000	-1.545502000	-0.072637000
1	1.696133000	-1.768104000	-1.066923000
1	2.592511000	-0.958228000	0.020097000
6	0.612643000	-0.731747000	0.291743000
6	0.642381000	0.687245000	-0.289412000
6	-0.673144000	-1.448762000	-0.095310000
1	0.623383000	-0.621788000	1.395843000
6	-0.612272000	1.466376000	0.102569000
1	0.666544000	0.584107000	-1.391429000
6	-1.916969000	-0.663346000	0.298946000
1	-0.669722000	-1.595210000	-1.192000000
1	-0.668687000	-2.452008000	0.352416000
6	-1.888281000	0.737837000	-0.301021000
1	-0.576409000	2.475484000	-0.337491000
1	-0.601640000	1.604736000	1.199854000
1	-2.823706000	-1.200254000	-0.013407000
1	-1.964042000	-0.585174000	1.398462000
1	-2.774531000	1.311701000	0.004572000
1	-1.929587000	0.661659000	-1.401215000
7	1.894844000	1.330176000	0.106571000
1	1.985357000	2.231856000	-0.358146000
1	1.855762000	1.532882000	1.106743000

N3

7	1.371708000	2.815912000	0.417302000

1	2.057240000	2.738035000	-0.330324000
1	0.743684000	3.580620000	0.167376000
6	0.576254000	1.600427000	0.499951000
6	-0.608427000	1.576068000	-0.502511000
1	0.117238000	1.568276000	1.507372000
1	-0.174172000	1.583380000	-1.517191000
7	-1.344363000	2.831322000	-0.353607000
1	-2.108749000	2.865541000	-1.025053000
1	-1.775027000	2.871882000	0.570045000
6	1.427115000	0.364059000	0.345628000
6	2.217056000	0.181567000	-0.792213000
6	1.395817000	-0.650511000	1.301018000
6	2.959294000	-0.980761000	-0.967906000
1	2.246846000	0.953141000	-1.564461000
6	2.132778000	-1.817481000	1.128874000
1	0.772540000	-0.524876000	2.187045000
6	2.918556000	-1.985930000	-0.006334000
1	3.569892000	-1.103487000	-1.861414000
1	2.092792000	-2.598607000	1.886550000
1	3.497617000	-2.897920000	-0.142544000
6	-1.430371000	0.316109000	-0.344164000
6	-1.302971000	-0.737509000	-1.250395000
6	-2.287713000	0.152315000	0.746583000
6	-2.010843000	-1.921574000	-1.075594000
1	-0.625226000	-0.629015000	-2.098567000
6	-2.996788000	-1.029912000	0.927875000
1	-2.405715000	0.959385000	1.472256000
6	-2.860992000	-2.071897000	0.015505000
1	-1.896969000	-2.731113000	-1.795110000
1	-3.659122000	-1.137976000	1.785747000
1	-3.416886000	-2.997723000	0.154512000

N4

6	-1.099633000	-0.999869000	0.048235000
6	1.548848000	-0.609239000	-0.055116000
7	-0.439587000	-1.915194000	0.622253000
7	0.894985000	0.297369000	-0.654131000
6	-0.545823000	0.294604000	-0.503355000
8	-2.446918000	-1.170885000	0.009314000
6	-3.182039000	-0.655807000	-1.079267000
1	-3.226400000	0.443529000	-1.084648000
1	-2.780321000	-1.004421000	-2.041602000
1	-4.198543000	-1.042202000	-0.964412000
6	0.982573000	-1.736135000	0.755063000
1	1.496150000	-2.668566000	0.476777000
1	1.242334000	-1.574323000	1.814701000
8	2.889251000	-0.654521000	-0.087492000
6	3.530194000	0.398376000	-0.789435000
1	3.265922000	1.371548000	-0.356296000
1	4.602896000	0.214797000	-0.692889000
1	3.233892000	0.401055000	-1.844964000
6	-0.996314000	1.488106000	0.375444000
1	-2.095132000	1.431709000	0.448033000
6	-0.619053000	2.805207000	-0.285888000
1	-0.963538000	3.657177000	0.314897000
1	0.470431000	2.880569000	-0.396849000
1	-1.063665000	2.894982000	-1.287162000
6	-0.432601000	1.398381000	1.786282000
1	-0.713711000	0.459252000	2.282601000
1	0.664431000	1.469076000	1.774069000
1	-0.808635000	2.225388000	2.402186000
1	-0.969475000	0.466706000	-1.507639000

H_2

1	0.000000000	0.000000000	0.371413000
1	0.000000000	0.000000000	-0.371413000

B

6	2.454122000	-0.115832000	0.727990000
6	1.339320000	-0.518989000	-0.009195000
6	1.511240000	-1.642924000	-0.818746000
6	2.712852000	-2.327851000	-0.910501000
6	3.795085000	-1.897786000	-0.150690000
6	3.666986000	-0.790710000	0.678581000
5	-0.006627000	0.289058000	0.020178000
6	-1.375701000	-0.475495000	0.043681000
6	-2.506835000	0.018629000	-0.614171000
6	-3.735969000	-0.626744000	-0.578400000
6	-3.867147000	-1.800607000	0.152560000
6	-2.771370000	-2.325311000	0.828080000
6	-1.554012000	-1.666129000	0.753998000
9	-2.434060000	1.123308000	-1.346616000
9	-4.779308000	-0.138241000	-1.231531000
9	-5.031350000	-2.420028000	0.204270000
9	-2.902482000	-3.440345000	1.530584000
9	-0.538932000	-2.201012000	1.428587000
9	0.503482000	-2.074980000	-1.575508000
9	2.842514000	-3.377466000	-1.707896000
9	4.944708000	-2.543807000	-0.216833000
9	4.697062000	-0.393314000	1.410061000
9	2.375068000	0.923934000	1.551990000
6	0.034215000	1.847335000	0.008139000
6	0.491414000	2.524524000	-1.135690000
6	0.510144000	3.917015000	-1.150173000
6	0.111698000	4.664058000	-0.043502000
6	-0.333684000	3.980279000	1.086828000
6	-0.388956000	2.589893000	1.124782000
1	0.844946000	4.435256000	-2.051694000
1	-0.650502000	4.549335000	1.963927000

6	0.927618000	1.758108000	-2.354891000
1	1.913850000	1.292072000	-2.212479000
1	0.223156000	0.951856000	-2.611606000
1	1.002025000	2.412448000	-3.231882000
6	-0.870038000	1.891863000	2.366971000
1	-1.833799000	1.387017000	2.206235000
1	-0.155077000	1.127376000	2.706612000
1	-1.007158000	2.597977000	3.194753000
6	0.180728000	6.161718000	-0.057809000
1	-0.611148000	6.608063000	0.556879000
1	1.139874000	6.520034000	0.342804000
1	0.086387000	6.560576000	-1.075451000

TS-N1B

6	1.462263000	-1.243237000	0.790319000
6	0.395323000	-0.381688000	1.031183000
6	0.494954000	0.374365000	2.196490000
6	1.577237000	0.294812000	3.064956000
6	2.630639000	-0.560428000	2.767792000
6	2.572243000	-1.335856000	1.618748000
5	-0.841340000	-0.334823000	0.010888000
6	-1.700136000	1.013664000	-0.064078000
6	-3.093164000	1.034522000	0.001982000
6	-3.835461000	2.209501000	-0.083035000
6	-3.184839000	3.423164000	-0.242426000
6	-1.797017000	3.452036000	-0.306303000
6	-1.095332000	2.261538000	-0.209182000
9	-3.790508000	-0.083701000	0.175531000
9	-5.159077000	2.177671000	-0.011372000
9	-3.877461000	4.547144000	-0.329648000
9	-1.164067000	4.609153000	-0.457324000
9	0.242124000	2.350006000	-0.269526000
9	-0.488063000	1.204781000	2.549069000

9	1.617604000	1.028971000	4.168457000
9	3.681592000	-0.629883000	3.569450000
9	3.601791000	-2.107536000	1.282567000
9	1.497661000	-1.973351000	-0.330103000
6	-1.535263000	-1.729302000	-0.316935000
6	-1.929771000	-2.556199000	0.759239000
6	-2.524198000	-3.792298000	0.516083000
6	-2.751433000	-4.260185000	-0.774631000
6	-2.404700000	-3.424825000	-1.829721000
6	-1.817545000	-2.175186000	-1.624514000
1	-0.064449000	-0.131245000	-1.314874000
1	0.673190000	0.206225000	-1.473147000
6	3.205281000	0.711808000	-1.006986000
6	2.467169000	0.072705000	-3.222799000
6	4.565623000	1.132665000	-1.553695000
1	3.276356000	-0.346022000	-0.708570000
1	2.918619000	1.294029000	-0.117998000
6	3.827092000	0.491567000	-3.770061000
1	2.471670000	-0.998533000	-2.960337000
1	1.665396000	0.226818000	-3.958669000
1	4.523672000	2.206069000	-1.816218000
1	4.098040000	-0.118523000	-4.642211000
1	3.767449000	1.535986000	-4.120553000
7	2.125344000	0.801414000	-1.995799000
1	1.934035000	1.782510000	-2.205853000
7	4.896225000	0.416485000	-2.784830000
1	5.084638000	-0.560973000	-2.555380000
6	5.642349000	0.904347000	-0.512801000
1	6.630843000	1.150022000	-0.916837000
1	5.465851000	1.513566000	0.382796000
1	5.651661000	-0.150664000	-0.196162000
1	-2.834277000	-4.405266000	1.365947000

1	-2.609759000	-3.749715000	-2.852563000
6	-1.792353000	-2.125149000	2.196573000
1	-0.773578000	-2.270273000	2.585196000
1	-2.048736000	-1.066822000	2.343747000
1	-2.465403000	-2.709295000	2.836345000
6	-1.513038000	-1.352365000	-2.847168000
1	-1.800148000	-0.298761000	-2.727948000
1	-0.440443000	-1.367943000	-3.095722000
1	-2.051898000	-1.743608000	-3.718808000
6	-3.355115000	-5.611328000	-1.014545000
1	-3.862672000	-5.660179000	-1.986193000
1	-2.588085000	-6.399196000	-1.009558000
1	-4.085472000	-5.870585000	-0.237264000

TS-N2B

6	1.135493000	-1.556049000	0.501934000
6	0.237504000	-0.550894000	0.857183000
6	0.582709000	0.181152000	1.990747000
6	1.749162000	-0.034973000	2.715877000
6	2.627850000	-1.028365000	2.307489000
6	2.306716000	-1.804988000	1.203962000
5	-1.090952000	-0.305770000	-0.008210000
6	-1.710609000	1.164480000	-0.019862000
6	-3.068935000	1.428398000	0.155251000
6	-3.595514000	2.716837000	0.120597000
6	-2.754962000	3.797179000	-0.101767000
6	-1.393288000	3.581713000	-0.278069000
6	-0.907629000	2.286083000	-0.224091000
9	-3.935775000	0.447854000	0.386742000
9	-4.893953000	2.919949000	0.298241000
9	-3.243540000	5.026076000	-0.142768000
9	-0.580604000	4.609381000	-0.490549000
9	0.412306000	2.132155000	-0.391674000

9	-0.231817000	1.129151000	2.455986000
9	2.030472000	0.696413000	3.784998000
9	3.761358000	-1.235924000	2.958371000
9	3.162578000	-2.742935000	0.791467000
9	0.933167000	-2.289974000	-0.593251000
6	-2.014792000	-1.567471000	-0.270526000
6	-2.379272000	-2.378014000	0.828362000
6	-3.170837000	-3.508291000	0.636281000
6	-3.626717000	-3.882852000	-0.623831000
6	-3.307706000	-3.055576000	-1.694527000
6	-2.529373000	-1.907886000	-1.539367000
1	-0.376515000	-0.164235000	-1.430080000
1	0.310981000	-0.009277000	-1.852203000
7	1.775041000	0.067875000	-2.686479000
1	1.807188000	0.667547000	-3.510418000
1	1.907171000	-0.903011000	-2.990250000
6	2.871189000	0.380935000	-1.762823000
6	4.116486000	-0.469076000	-2.036448000
6	3.229933000	1.857618000	-1.762576000
1	2.516478000	0.129560000	-0.745271000
6	5.156112000	-0.204324000	-0.948711000
1	4.528061000	-0.136672000	-3.008473000
6	4.265043000	2.142908000	-0.679943000
1	3.641074000	2.124600000	-2.753637000
1	2.327721000	2.463796000	-1.612449000
6	5.509251000	1.277830000	-0.860586000
1	6.055572000	-0.808721000	-1.140727000
1	4.747219000	-0.557835000	0.016593000
1	4.533829000	3.207806000	-0.678007000
1	3.810112000	1.935302000	0.304121000
1	6.223664000	1.454716000	-0.044949000
1	6.020617000	1.577312000	-1.791435000

7	3.723856000	-1.864090000	-2.205766000
1	4.505999000	-2.410131000	-2.559135000
1	3.469746000	-2.271069000	-1.305605000
6	-1.997549000	-2.033963000	2.245677000
1	-0.970830000	-2.341543000	2.493520000
1	-2.071668000	-0.957118000	2.453103000
1	-2.663516000	-2.542384000	2.953907000
6	-4.439238000	-5.127887000	-0.816574000
1	-3.795348000	-6.004548000	-0.978019000
1	-5.061262000	-5.343839000	0.061525000
1	-5.100014000	-5.047885000	-1.689015000
6	-2.281064000	-1.073844000	-2.766553000
1	-2.385881000	0.001624000	-2.570785000
1	-1.270266000	-1.226410000	-3.173425000
1	-2.992431000	-1.336227000	-3.559200000
1	-3.452845000	-4.109460000	1.504136000
1	-3.689876000	-3.302945000	-2.687758000

TS-N3B

6	0.073497000	-2.008333000	0.580119000
6	-0.606037000	-0.857523000	0.979473000
6	-0.252980000	-0.368437000	2.235201000
6	0.711813000	-0.962177000	3.042027000
6	1.358502000	-2.109455000	2.601174000
6	1.020765000	-2.646856000	1.367082000
5	-1.774762000	-0.247614000	0.068614000
6	-2.130421000	1.298979000	0.179320000
6	-3.442459000	1.776753000	0.143617000
6	-3.753557000	3.131978000	0.208161000
6	-2.733919000	4.066134000	0.305420000
6	-1.412597000	3.637611000	0.349785000
6	-1.143632000	2.279571000	0.298825000
9	-4.476111000	0.946609000	0.063037000

9	-5.015831000	3.536525000	0.174689000
9	-3.014754000	5.357258000	0.358885000
9	-0.431865000	4.525920000	0.443500000
9	0.143095000	1.925905000	0.361130000
9	-0.878662000	0.692673000	2.744652000
9	1.004737000	-0.456982000	4.232034000
9	2.275832000	-2.695368000	3.352451000
9	1.622129000	-3.753148000	0.939042000
9	-0.140783000	-2.539151000	-0.630133000
6	-2.850711000	-1.279101000	-0.469785000
6	-3.507593000	-2.090014000	0.482620000
6	-4.452243000	-3.028931000	0.075177000
6	-4.778096000	-3.208373000	-1.265604000
6	-4.161917000	-2.376993000	-2.194245000
6	-3.222933000	-1.413299000	-1.824023000
1	-0.797554000	-0.246558000	-1.279658000
1	-0.362574000	0.324315000	-1.634385000
7	0.937347000	1.227977000	-2.364869000
1	0.937321000	2.241466000	-2.296571000
1	0.948973000	0.918307000	-3.335212000
6	2.027913000	0.612353000	-1.600957000
6	2.823076000	-0.361630000	-2.495782000
1	1.577407000	-0.012637000	-0.803917000
1	3.338180000	0.243708000	-3.262905000
7	1.854979000	-1.203526000	-3.194159000
1	2.338291000	-1.899336000	-3.757986000
1	1.280368000	-1.716203000	-2.523927000
6	2.929249000	1.613170000	-0.926933000
6	3.516437000	2.651502000	-1.652170000
6	3.245081000	1.470801000	0.423714000
6	4.410606000	3.524094000	-1.041254000
1	3.280402000	2.771052000	-2.711693000

6	4.141963000	2.337501000	1.036566000
1	2.786707000	0.658589000	0.992825000
6	4.728226000	3.365734000	0.304447000
1	4.863360000	4.328749000	-1.618621000
1	4.381459000	2.212009000	2.091053000
1	5.429221000	4.047091000	0.783673000
6	3.871541000	-1.068159000	-1.664172000
6	5.184773000	-0.597476000	-1.632592000
6	3.519868000	-2.127066000	-0.827311000
6	6.120052000	-1.157597000	-0.769623000
1	5.468823000	0.237968000	-2.274560000
6	4.447681000	-2.682693000	0.047858000
1	2.506612000	-2.528453000	-0.853723000
6	5.751007000	-2.196130000	0.080132000
1	7.139674000	-0.775598000	-0.755055000
1	4.145203000	-3.493925000	0.708373000
1	6.479172000	-2.628313000	0.764554000
1	-4.960449000	-3.630860000	0.832486000
1	-4.432790000	-2.469408000	-3.248610000
6	-3.271696000	-1.936427000	1.963900000
1	-2.377654000	-2.478736000	2.306844000
1	-3.149211000	-0.886519000	2.267090000
1	-4.123153000	-2.334443000	2.529741000
6	-2.656965000	-0.550842000	-2.920685000
1	-1.644525000	-0.863965000	-3.220077000
1	-3.290389000	-0.608279000	-3.814284000
1	-2.589727000	0.506990000	-2.632018000
6	-5.764472000	-4.254366000	-1.690344000
1	-6.547594000	-4.400661000	-0.935389000
1	-6.251469000	-3.989443000	-2.637284000
1	-5.275562000	-5.227977000	-1.839023000

TS-N4B

6	1.980802000	2.171860000	-0.458900000
6	1.659580000	1.163236000	0.447066000
6	1.609942000	1.560898000	1.779810000
6	1.846846000	2.864521000	2.199209000
6	2.138749000	3.840716000	1.256190000
6	2.199796000	3.492262000	-0.085633000
5	1.385624000	-0.336369000	-0.091969000
6	0.273258000	-1.209743000	0.698750000
6	0.332845000	-2.601803000	0.803792000
6	-0.660136000	-3.367204000	1.414882000
6	-1.772332000	-2.744729000	1.958817000
6	-1.869186000	-1.361708000	1.897925000
6	-0.865334000	-0.639005000	1.271583000
9	1.355346000	-3.288380000	0.306523000
9	-0.550907000	-4.687089000	1.474641000
9	-2.735773000	-3.454868000	2.524980000
9	-2.929342000	-0.748139000	2.414004000
9	-1.070344000	0.689248000	1.216892000
6	2.672464000	-1.070794000	-0.709408000
6	3.820210000	-1.230039000	0.095757000
6	4.973477000	-1.820345000	-0.420698000
6	5.044159000	-2.271803000	-1.733093000
6	3.899221000	-2.151263000	-2.513535000
6	2.726369000	-1.573005000	-2.027970000
1	0.784750000	0.054310000	-1.306502000
1	-0.053431000	-0.028384000	-1.414687000
6	-2.011551000	1.121732000	-1.290501000
6	-3.582600000	-0.996049000	-0.754536000
7	-1.538993000	-0.007255000	-1.664796000
7	-4.056698000	0.110238000	-0.353711000
6	-3.415891000	1.331565000	-0.790359000
8	-1.159048000	2.144368000	-1.383775000

6	-1.320382000	3.326696000	-0.611382000
1	-2.277645000	3.824994000	-0.808076000
1	-1.220874000	3.105471000	0.458830000
1	-0.505560000	3.985985000	-0.922988000
6	-2.380261000	-1.180465000	-1.634493000
1	-1.792605000	-2.037862000	-1.276606000
1	-2.708340000	-1.442810000	-2.652831000
8	-4.119349000	-2.172443000	-0.421687000
6	-5.258684000	-2.128780000	0.432378000
1	-6.080967000	-1.586341000	-0.048920000
1	-5.533960000	-3.170332000	0.609784000
1	-5.006451000	-1.630265000	1.373959000
6	-4.275911000	2.067155000	-1.847473000
1	-3.728922000	2.985808000	-2.120022000
6	-5.617418000	2.460777000	-1.247374000
1	-6.220402000	3.015251000	-1.977611000
1	-6.179282000	1.568451000	-0.943927000
1	-5.492276000	3.094661000	-0.358907000
6	-4.457879000	1.241275000	-3.112915000
1	-3.498478000	0.974156000	-3.577648000
1	-5.012356000	0.316488000	-2.900848000
1	-5.035897000	1.805812000	-3.855057000
1	-3.356260000	1.988912000	0.093511000
6	3.847899000	-0.807314000	1.539128000
1	4.015396000	0.274197000	1.649148000
1	2.915453000	-1.041384000	2.068190000
1	4.661504000	-1.313166000	2.073652000
6	6.304366000	-2.866077000	-2.287045000
1	6.903928000	-3.344191000	-1.501877000
1	6.091437000	-3.619200000	-3.056633000
1	6.938302000	-2.098629000	-2.754443000
6	1.542004000	-1.548809000	-2.958266000

1	0.643051000	-1.983333000	-2.494559000
1	1.279078000	-0.531766000	-3.284960000
1	1.751693000	-2.135014000	-3.861199000
1	5.842862000	-1.938277000	0.230918000
1	3.914758000	-2.533044000	-3.537236000
9	1.320352000	0.676338000	2.739410000
9	1.789229000	3.186479000	3.485902000
9	2.348927000	5.094237000	1.632772000
9	2.437992000	4.426446000	-1.001352000
9	2.048282000	1.903785000	-1.764839000

TS-N1B-Sol

6	-1.298063000	1.579954000	0.558073000
6	-0.336220000	0.618395000	0.849544000
6	-0.452984000	0.070229000	2.125203000
6	-1.429268000	0.448815000	3.042496000
6	-2.367383000	1.411306000	2.692224000
6	-2.302457000	1.980408000	1.429302000
5	0.792555000	0.260306000	-0.295597000
6	1.481612000	-1.214135000	-0.093232000
6	2.846908000	-1.482707000	-0.023065000
6	3.370822000	-2.771998000	0.064009000
6	2.521514000	-3.867090000	0.085041000
6	1.151417000	-3.655086000	0.018230000
6	0.686137000	-2.353767000	-0.068354000
9	3.746808000	-0.501040000	-0.031725000
9	4.683245000	-2.962896000	0.126920000
9	3.007134000	-5.099267000	0.165250000
9	0.311884000	-4.688264000	0.030898000
9	-0.671852000	-2.228994000	-0.131333000
9	0.404925000	-0.862912000	2.546237000
9	-1.481964000	-0.106738000	4.246943000
9	-3.322378000	1.764640000	3.542910000

9	-3.239919000	2.841515000	1.032932000
9	-1.357721000	2.113886000	-0.682449000
6	1.776168000	1.524925000	-0.546328000
6	2.435112000	2.190241000	0.510664000
6	3.235015000	3.306191000	0.266512000
6	3.420322000	3.814638000	-1.014796000
6	2.792647000	3.149781000	-2.061479000
6	1.986849000	2.029212000	-1.847452000
1	0.150261000	0.112072000	-1.340819000
1	-1.310011000	-0.148868000	-1.296158000
6	-3.343270000	-0.441846000	-0.725380000
6	-2.477211000	-0.059858000	-3.024502000
6	-4.582400000	-1.102202000	-1.318944000
1	-3.499229000	0.638632000	-0.596871000
1	-3.037953000	-0.872036000	0.237146000
6	-3.738650000	-0.718211000	-3.561284000
1	-2.594931000	1.024424000	-2.900053000
1	-1.590833000	-0.241872000	-3.644320000
1	-4.384145000	-2.186375000	-1.408455000
1	-3.997885000	-0.279439000	-4.531876000
1	-3.539755000	-1.787283000	-3.745017000
7	-2.189162000	-0.597672000	-1.662573000
1	-1.931775000	-1.591522000	-1.708682000
7	-4.873067000	-0.619385000	-2.663118000
1	-5.225352000	0.337009000	-2.622544000
6	-5.776927000	-0.886613000	-0.413532000
1	-6.665394000	-1.359753000	-0.844659000
1	-5.603823000	-1.305706000	0.584718000
1	-5.983612000	0.187040000	-0.294134000
1	3.739544000	3.789815000	1.107532000
1	2.940759000	3.511165000	-3.082649000
6	2.326406000	1.726021000	1.937773000

1	1.413799000	2.103807000	2.424494000
1	2.310503000	0.633689000	2.027633000
1	3.175881000	2.091233000	2.529151000
6	1.369877000	1.393232000	-3.066185000
1	1.552435000	0.310324000	-3.109090000
1	0.277692000	1.538202000	-3.087408000
1	1.776024000	1.836351000	-3.984810000
6	4.259409000	5.034994000	-1.252855000
1	4.635576000	5.071530000	-2.283440000
1	3.687026000	5.959085000	-1.084188000
1	5.124252000	5.068015000	-0.577023000

TS-N2B-Sol

6	1.037137000	-1.686528000	0.293377000
6	0.246262000	-0.572952000	0.577673000
6	0.715126000	0.210006000	1.630122000
6	1.881699000	-0.065481000	2.341363000
6	2.632167000	-1.186247000	2.017049000
6	2.194308000	-2.011089000	0.990769000
5	-1.064085000	-0.288396000	-0.380074000
6	-1.733835000	1.194899000	-0.183994000
6	-3.026661000	1.477616000	0.250862000
6	-3.551212000	2.768295000	0.305389000
6	-2.775445000	3.850403000	-0.079634000
6	-1.477151000	3.624635000	-0.515443000
6	-1.010481000	2.321847000	-0.553162000
9	-3.849107000	0.511804000	0.658292000
9	-4.793072000	2.973684000	0.729429000
9	-3.262603000	5.084094000	-0.030019000
9	-0.706884000	4.645157000	-0.888185000
9	0.273428000	2.175624000	-0.982240000
9	0.047880000	1.296813000	2.023580000
9	2.300008000	0.744230000	3.307932000

9	3.775127000	-1.444284000	2.643990000
9	2.955083000	-3.048266000	0.612729000
9	0.752792000	-2.464100000	-0.760194000
6	-2.085481000	-1.544820000	-0.354682000
6	-2.511696000	-2.142733000	0.850161000
6	-3.361395000	-3.249060000	0.837339000
6	-3.822357000	-3.809477000	-0.349370000
6	-3.421710000	-3.209580000	-1.538185000
6	-2.572122000	-2.101875000	-1.555585000
1	-0.576692000	-0.227740000	-1.514682000
1	0.903730000	0.309280000	-1.790506000
7	1.846193000	0.261275000	-2.246839000
1	1.858111000	0.932035000	-3.020531000
1	1.982836000	-0.707617000	-2.612688000
6	2.993600000	0.466297000	-1.298372000
6	4.162659000	-0.382832000	-1.799678000
6	3.377026000	1.915704000	-1.089904000
1	2.647322000	0.047522000	-0.347331000
6	5.276697000	-0.330739000	-0.758727000
1	4.532316000	0.075573000	-2.736356000
6	4.505682000	1.972592000	-0.060383000
1	3.717940000	2.349434000	-2.047269000
1	2.509625000	2.495263000	-0.751955000
6	5.694291000	1.111246000	-0.478424000
1	6.131639000	-0.927186000	-1.107418000
1	4.915967000	-0.811255000	0.168077000
1	4.817340000	3.013852000	0.089787000
1	4.118036000	1.622622000	0.911319000
1	6.473149000	1.136058000	0.294496000
1	6.147213000	1.537013000	-1.389838000
7	3.648676000	-1.702871000	-2.150531000
1	4.303068000	-2.211915000	-2.738272000

1	3.483153000	-2.271916000	-1.318139000
6	-2.098365000	-1.609316000	2.196667000
1	-1.110755000	-1.984699000	2.508046000
1	-2.049339000	-0.513729000	2.217487000
1	-2.813219000	-1.917886000	2.970595000
6	-4.711595000	-5.017709000	-0.345645000
1	-4.128681000	-5.950537000	-0.328421000
1	-5.366607000	-5.033701000	0.535461000
1	-5.348251000	-5.052870000	-1.239378000
6	-2.210521000	-1.529342000	-2.900518000
1	-2.402954000	-0.448844000	-2.955766000
1	-1.142999000	-1.673126000	-3.130400000
1	-2.787265000	-2.012851000	-3.699806000
1	-3.683650000	-3.681109000	1.788863000
1	-3.788314000	-3.613895000	-2.485643000

TS-N3B-Sol

6	0.499099000	-1.511323000	0.996343000
6	-0.505139000	-0.548846000	1.055776000
6	-0.562960000	0.139770000	2.264012000
6	0.290169000	-0.109878000	3.336112000
6	1.261246000	-1.095933000	3.227700000
6	1.363912000	-1.810041000	2.041490000
5	-1.502017000	-0.377786000	-0.237095000
6	-2.358773000	1.020226000	-0.228581000
6	-3.739624000	1.148745000	-0.097457000
6	-4.403966000	2.372427000	-0.150646000
6	-3.686036000	3.543472000	-0.337902000
6	-2.306556000	3.472425000	-0.470160000
6	-1.696731000	2.229455000	-0.408139000
9	-4.518126000	0.084926000	0.099684000
9	-5.724667000	2.429913000	-0.020602000
9	-4.306863000	4.715455000	-0.393605000

9	-1.594531000	4.581825000	-0.657670000
9	-0.344414000	2.235812000	-0.547213000
9	-1.498850000	1.070309000	2.474462000
9	0.177037000	0.574153000	4.469093000
9	2.075372000	-1.357353000	4.242391000
9	2.287791000	-2.765324000	1.920562000
9	0.690075000	-2.213755000	-0.144549000
6	-2.342035000	-1.733121000	-0.538264000
6	-3.001132000	-2.468739000	0.471194000
6	-3.669115000	-3.655317000	0.170157000
6	-3.724668000	-4.164663000	-1.123519000
6	-3.087497000	-3.439014000	-2.122604000
6	-2.409345000	-2.248623000	-1.849638000
1	-0.713966000	-0.253444000	-1.171594000
1	0.110852000	0.754546000	-1.972830000
7	1.006147000	0.835081000	-2.505934000
1	1.101545000	1.780883000	-2.884604000
1	1.011282000	0.106819000	-3.249737000
6	2.138595000	0.469268000	-1.570911000
6	3.156184000	-0.337887000	-2.389342000
1	1.671479000	-0.212270000	-0.847123000
1	3.590291000	0.326683000	-3.156259000
7	2.383828000	-1.356345000	-3.107020000
1	2.969034000	-1.846036000	-3.779723000
1	2.033333000	-2.056924000	-2.450824000
6	2.678733000	1.674561000	-0.863476000
6	3.311236000	2.700697000	-1.568938000
6	2.549841000	1.769776000	0.520522000
6	3.801459000	3.813679000	-0.896254000
1	3.434371000	2.633468000	-2.653096000
6	3.046788000	2.879112000	1.194271000
1	2.048239000	0.968871000	1.065632000

6	3.669808000	3.902109000	0.486893000
1	4.290600000	4.611732000	-1.451532000
1	2.938303000	2.946449000	2.274833000
1	4.053363000	4.773544000	1.014064000
6	4.272094000	-0.828468000	-1.498799000
6	5.571146000	-0.354053000	-1.671497000
6	4.008494000	-1.732559000	-0.467892000
6	6.595917000	-0.779197000	-0.832001000
1	5.780581000	0.362496000	-2.467610000
6	5.028896000	-2.150892000	0.377590000
1	3.000964000	-2.119075000	-0.312028000
6	6.325361000	-1.677109000	0.195105000
1	7.607093000	-0.403500000	-0.978634000
1	4.800335000	-2.842785000	1.185695000
1	7.124425000	-2.005644000	0.857238000
1	-4.172030000	-4.198281000	0.975319000
1	-3.121831000	-3.809481000	-3.150817000
6	-3.026877000	-2.014820000	1.906737000
1	-2.110161000	-2.298649000	2.447331000
1	-3.136082000	-0.928178000	2.002581000
1	-3.867219000	-2.475746000	2.441888000
6	-1.753876000	-1.556368000	-3.017508000
1	-0.653397000	-1.620709000	-2.960450000
1	-2.056489000	-2.019456000	-3.965870000
1	-2.017000000	-0.489461000	-3.071103000
6	-4.474820000	-5.427766000	-1.427638000
1	-5.559023000	-5.251337000	-1.482069000
1	-4.167607000	-5.857902000	-2.389708000
1	-4.315402000	-6.189121000	-0.652192000

TS-N4B-Sol

6	1.918491000	2.259297000	-0.465998000
6	1.639681000	1.210635000	0.408485000

6	1.563460000	1.585868000	1.746704000
6	1.743278000	2.888887000	2.200416000
6	2.005105000	3.898937000	1.285675000
6	2.087999000	3.578830000	-0.061536000
5	1.377498000	-0.278036000	-0.225891000
6	0.307095000	-1.193026000	0.637045000
6	0.383084000	-2.582248000	0.766351000
6	-0.595488000	-3.358739000	1.390721000
6	-1.715439000	-2.752163000	1.938141000
6	-1.834312000	-1.373306000	1.852290000
6	-0.848232000	-0.651258000	1.195974000
9	1.413565000	-3.264603000	0.273286000
9	-0.465338000	-4.677567000	1.464171000
9	-2.670356000	-3.471599000	2.517498000
9	-2.904033000	-0.763799000	2.365420000
9	-1.104548000	0.678289000	1.091304000
6	2.744959000	-1.009471000	-0.701062000
6	3.851996000	-1.188855000	0.154177000
6	5.031494000	-1.773104000	-0.309945000
6	5.170800000	-2.207840000	-1.622926000
6	4.068527000	-2.071449000	-2.459938000
6	2.876389000	-1.493059000	-2.021625000
1	0.767579000	-0.007911000	-1.255561000
1	-0.599805000	-0.131211000	-1.920498000
6	-2.048432000	1.076703000	-1.333936000
6	-3.566902000	-1.113287000	-0.769312000
7	-1.629274000	-0.071692000	-1.783240000
7	-4.034768000	-0.009959000	-0.354050000
6	-3.422142000	1.230391000	-0.757343000
8	-1.209820000	2.059462000	-1.482421000
6	-1.276534000	3.264099000	-0.702479000
1	-2.249482000	3.755621000	-0.806778000

1	-1.058537000	3.024490000	0.343597000
1	-0.488491000	3.900223000	-1.110577000
6	-2.385324000	-1.305667000	-1.676036000
1	-1.723077000	-2.083153000	-1.272015000
1	-2.721920000	-1.637831000	-2.668429000
8	-4.090264000	-2.291953000	-0.452796000
6	-5.176817000	-2.284617000	0.474848000
1	-6.019210000	-1.709658000	0.074491000
1	-5.450749000	-3.332113000	0.609973000
1	-4.852945000	-1.844469000	1.423456000
6	-4.331245000	2.010872000	-1.744866000
1	-3.800784000	2.942614000	-2.004088000
6	-5.639663000	2.372773000	-1.058306000
1	-6.276569000	2.956877000	-1.733910000
1	-6.185937000	1.467225000	-0.767330000
1	-5.470141000	2.968205000	-0.151366000
6	-4.574256000	1.233109000	-3.030353000
1	-3.642772000	0.993190000	-3.562876000
1	-5.110785000	0.296514000	-2.826045000
1	-5.195326000	1.822167000	-3.715760000
1	-3.309293000	1.847727000	0.149679000
6	3.802048000	-0.789308000	1.602370000
1	3.913627000	0.296915000	1.735081000
1	2.854673000	-1.069509000	2.079192000
1	4.612617000	-1.267421000	2.167167000
6	6.457619000	-2.798764000	-2.117718000
1	7.004104000	-3.306729000	-1.312256000
1	6.285202000	-3.527856000	-2.920454000
1	7.128132000	-2.026822000	-2.523409000
6	1.737411000	-1.456112000	-3.008114000
1	0.841606000	-1.961128000	-2.608544000
1	1.449231000	-0.428549000	-3.275512000

1	2.008716000	-1.975011000	-3.936821000
1	5.867099000	-1.901458000	0.383547000
1	4.136390000	-2.438534000	-3.487633000
9	1.278153000	0.679896000	2.691484000
9	1.654487000	3.179916000	3.495047000
9	2.159284000	5.154841000	1.691514000
9	2.274877000	4.546964000	-0.960456000
9	1.986179000	2.043272000	-1.785307000

TSN4B

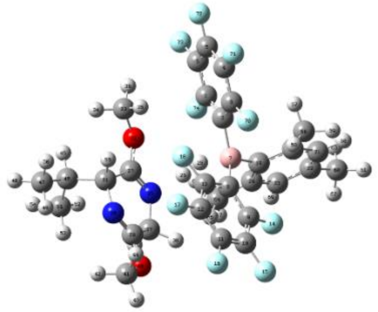

Atom	No	Charge	Core	Valence	Rydberg	Total
C	1	0.39974	1.99834	3.57330	0.02862	5.60026
C	2	-0.41119	1.99866	4.39330	0.01923	6.41119
C	3	0.39350	1.99834	3.57936	0.02880	5.60650
C	4	0.30687	1.99834	3.66635	0.02844	5.69313
C	5	0.32031	1.99840	3.65285	0.02844	5.67969
C	6	0.29777	1.99835	3.67493	0.02895	5.70223
B	7	-0.50096	1.99897	2.47519	0.02488	4.49904
C	8	-0.43188	1.99866	4.41386	0.01935	6.43188
C	9	0.40895	1.99839	3.56473	0.02793	5.59105
C	10	0.30959	1.99837	3.66327	0.02877	5.69041
C	11	0.33385	1.99838	3.63867	0.02910	5.66615
C	12	0.30203	1.99832	3.66999	0.02967	5.69797

C	13	0.38714	1.99835	3.58508	0.02943	5.61286
F	14	-0.32074	1.99993	7.31352	0.00730	9.32074
F	15	-0.32258	1.99994	7.31491	0.00773	9.32258
F	16	-0.32102	1.99993	7.31338	0.00771	9.32102
F	17	-0.32530	1.99993	7.31769	0.00768	9.32530
F	18	-0.34612	1.99993	7.33937	0.00682	9.34612
C	19	-0.31756	1.99875	4.30091	0.01790	6.31756
C	20	0.01082	1.99902	3.97624	0.01392	5.98918
C	21	-0.23730	1.99896	4.22675	0.01158	6.23730
C	22	-0.01745	1.99902	4.00419	0.01424	6.01745
C	23	-0.23793	1.99896	4.22756	0.01140	6.23793
C	24	0.00645	1.99901	3.98011	0.01443	5.99355
H	25	-0.04400	0.00000	1.04171	0.00229	1.04400
H	26	0.31746	0.00000	0.67979	0.00276	0.68254
C	27	0.68963	1.99896	3.27552	0.03589	5.31037
C	28	0.62112	1.99907	3.34801	0.03180	5.37888
N	29	0.56876	1.99927	5.54881	0.02068	7.56876
N	30	0.56459	1.99931	5.54134	0.02394	7.56459
C	31	-0.15712	1.99902	4.13698	0.02112	6.15712
O	32	-0.53386	1.99970	6.52008	0.01407	8.53386
C	33	-0.32409	1.99938	4.31148	0.01323	6.32409
H	34	0.20976	0.00000	0.78900	0.00123	0.79024
H	35	0.22632	0.00000	0.77231	0.00137	0.77368
H	36	0.24582	0.00000	0.75309	0.00109	0.75418
C	37	-0.34244	1.99919	4.32688	0.01636	6.34244
H	38	0.26661	0.00000	0.73153	0.00186	0.73339
H	39	0.27057	0.00000	0.72790	0.00154	0.72943
O	40	-0.54397	1.99973	6.53100	0.01323	8.54397
C	41	-0.32845	1.99940	4.31481	0.01424	6.32845
H	42	0.21882	0.00000	0.77981	0.00136	0.78118
H	43	0.23188	0.00000	0.76759	0.00053	0.76812
H	44	0.23026	0.00000	0.76758	0.00216	0.76974

C	45	-0.24883	1.99919	4.23662	0.01302	6.24883
H	46	0.24191	0.00000	0.75617	0.00192	0.75809
C	47	-0.69394	1.99941	4.68501	0.00952	6.69394
H	48	0.24282	0.00000	0.75646	0.00072	0.75718
H	49	0.24841	0.00000	0.75037	0.00122	0.75159
H	50	0.23759	0.00000	0.76167	0.00074	0.76241
C	51	-0.70425	1.99941	4.69539	0.00946	6.70425
H	52	0.23842	0.00000	0.76063	0.00094	0.76158
H	53	0.23686	0.00000	0.76187	0.00127	0.76314
H	54	0.25089	0.00000	0.74843	0.00068	0.74911
H	55	0.27069	0.00000	0.72711	0.00221	0.72931
C	56	-0.70576	1.99939	4.69862	0.00775	6.70576
H	57	0.24257	0.00000	0.75640	0.00103	0.75743
H	58	0.24274	0.00000	0.75582	0.00144	0.75726
H	59	0.24432	0.00000	0.75487	0.00082	0.75568
C	60	-0.70387	1.99939	4.69780	0.00668	6.70387
H	61	0.24177	0.00000	0.75740	0.00083	0.75823
H	62	0.24149	0.00000	0.75766	0.00085	0.75851
H	63	0.24907	0.00000	0.74998	0.00095	0.75093
C	64	-0.70182	1.99939	4.69446	0.00797	6.70182
H	65	0.22670	0.00000	0.77183	0.00148	0.77330
H	66	0.24640	0.00000	0.75195	0.00165	0.75360
H	67	0.24349	0.00000	0.75574	0.00077	0.75651
H	68	0.22462	0.00000	0.77421	0.00117	0.77538
H	69	0.22347	0.00000	0.77539	0.00115	0.77653
F	70	-0.33618	1.99993	7.32934	0.00691	9.33618
F	71	-0.32597	1.99994	7.31845	0.00758	9.32597
F	72	-0.32345	1.99994	7.31585	0.00767	9.32345
F	73	-0.32842	1.99994	7.32087	0.00761	9.32842
F	74	-0.33161	1.99993	7.32471	0.00697	9.33161
